# Specification and survival of post-metamorphic branchiomeric neurons in a non-vertebrate chordate

**DOI:** 10.1242/dev.202719

**Published:** 2024-07-17

**Authors:** Eduardo D. Gigante, Katarzyna M. Piekarz, Alexandra Gurgis, Leslie Cohen, Florian Razy-Krajka, Sydney Popsuj, Christopher J. Johnson, Hussan S. Ali, Shruthi Mohana Sundaram, Alberto Stolfi

**Affiliations:** ^1^ School of Biological Sciences, College of Sciences, Georgia Institute of Technology, Atlanta, GA 30332, USA; ^2^ Department of Biology, Case Western Reserve University, Cleveland, OH 44106, USA

**Keywords:** Ciliomotor neurons, *Ciona*, Neck neurons, Pax2/5/8, Phox2

## Abstract

Tunicates are the sister group to the vertebrates, yet most species have a life cycle split between swimming larva and sedentary adult phases. During metamorphosis, larval neurons are replaced by adult-specific ones. The regulatory mechanisms underlying this replacement remain largely unknown. Using tissue-specific CRISPR/Cas9-mediated mutagenesis in the tunicate *Ciona*, we show that orthologs of conserved hindbrain and branchiomeric neuron regulatory factors Pax2/5/8 and Phox2 are required to specify the ‘neck’, a cellular compartment set aside in the larva to give rise to cranial motor neuron-like neurons post-metamorphosis. Using bulk and single-cell RNA-sequencing analyses, we characterize the transcriptome of the neck downstream of Pax2/5/8. We present evidence that neck-derived adult ciliomotor neurons begin to differentiate in the larva and persist through metamorphosis, contrary to the assumption that the adult nervous system is formed after settlement and the death of larval neurons during metamorphosis. Finally, we show that FGF signaling during the larval phase alters the patterning of the neck and its derivatives. Suppression of FGF converts neck cells into larval neurons that fail to survive metamorphosis, whereas prolonged FGF signaling promotes an adult neural stem cell-like fate.

## Teaser

**Summary:** Identification of neurons derived from the poorly understood neck region in *Ciona* that survive the near-complete overhaul of the body plan (metamorphosis) that non-vertebrate tunicate species undergo as part of their normal life cycle.

## INTRODUCTION

**Fig. 1.**
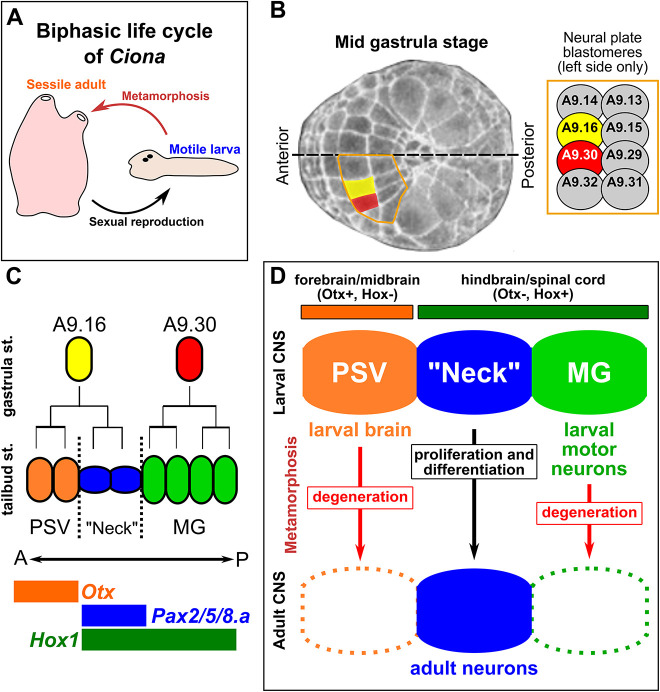
**Specification of the ‘neck’ during *Ciona* embryogenesis.** (A) A diagram of the biphasic life cycle of *Ciona* and many other tunicates, alternating between a motile, non-feeding larva (pre-metamorphic) and a sessile, filter-feeding juvenile/adult (post-metamorphic). (B) Image of a mid gastrula-stage *Ciona robusta* embryo (St. 12), adapted from the TUNICANATO database (Hotta et al., 2020), with the A9.16 (yellow) and A9.30 (red) blastomeres false-colored in the neural plate. The left half of the vegetal pole-derived neural plate is outlined in orange, and blastomere identities displayed in the inset on the right. The dorsal midline is indicated by the dashed line. (C) Simple diagram of the cell lineages derived from the A9.16 and A9.30 blastomeres giving rise to neurons, photoreceptors, and undifferentiated precursors of the posterior sensory vesicle region (PSV) in orange, the ‘neck’ in blue, and cells of the motor ganglion (MG) in green. The anterior (A)-posterior (P) axis is indicated. Colored bars indicate expression domains of conserved forebrain/midbrain regulatory gene *Otx* in orange, the rhombospinal (hindbrain/spinal cord) regulatory gene *Hox1* in green, and the neck/hindbrain marker *Pax2/5/8.a* in blue. (D) Diagram summarizing the ‘traditional’ view of the *Ciona* larval CNS, indicating proposed homology of the PSV, neck and MG compartments to vertebrate CNS partitions, based on *Otx* and *Hox* gene expression patterns. According to this view, differentiated larval neurons of the PSV/brain and MG are eliminated during metamorphosis, whereas the neck contributes neurons to the post-metamorphic, juvenile/adult nervous system.

The simple embryos of the non-vertebrate chordate *Ciona* and related tunicates constitute a highly tractable system in which to study the regulation of cellular processes in development (Farley et al., 2015; Bernadskaya and Christiaen, 2016; Cao et al., 2019; Wang et al., 2019; Guignard et al., 2020). Their classification as tunicates, the sister group to the vertebrates (Delsuc et al., 2006; Putnam et al., 2008), means they share with vertebrates many chordate-specific gene families, cell types, organs and anatomical structures (Christiaen et al., 2002; Dufour et al., 2006; Razy-Krajka et al., 2014; Abitua et al., 2015; Stolfi et al., 2015; Di Gregorio, 2020; Fodor et al., 2021; Lemaire et al., 2021; Papadogiannis et al., 2022). The simplicity of their embryos overshadows the fact that the larva is but one part of a biphasic life cycle alternating between a free-swimming larval phase and a sessile adult phase (Fig. 1A). During metamorphosis, most larval structures degenerate and are replaced by adult tissues (Chiba et al., 2004; Nakayama-Ishimura et al., 2009; Sasakura and Hozumi, 2018). This metamorphosis is not entirely catastrophic, as some adult structures begin to form during the larval phase (Nakazawa et al., 2013; Razy-Krajka et al., 2014), which is pronounced in colonial species (Berrill, 1935). Although the invariant cell lineages and gene regulatory networks specifying many larval cell types of *Ciona* have been investigated in detail (Imai et al., 2006; Cao et al., 2019), metamorphosis and adult development are still poorly understood. We know that various undifferentiated progenitor cells set aside in the larva as discrete compartments can give rise to adult structures (Dufour et al., 2006; Horie et al., 2011; Razy-Krajka et al., 2014; Sasakura and Hozumi, 2018). These adult progenitor cells are specified in an invariant manner alongside differentiated larval cells, but only differentiate after the larva settles and undergoes metamorphosis. The transition from invariant (stereotyped) embryogenesis to variable (plastic) post-metamorphic development is unique among the chordates, and thus of potential interest for revealing previously unknown mechanisms for precise spatiotemporal regulation of cellular quiescence, survival, proliferation and differentiation.

In this work, we focused on a compartment of *Ciona* adult neural precursor cells in the larva called the ‘neck’ (Fig. 1B,C). The neck has been proposed to be homologous to parts of the hindbrain of vertebrates based on shared expression of *Pax2/5/8.a*, *Phox2* and *Hox1* orthologs, specifically regions giving rise to branchiomeric motor neurons (Fig. 1C,D) (Dufour et al., 2006; Imai et al., 2009; Hudson and Yasuo, 2021). Although it is difficult to draw one-to-one correspondences between specific tunicate and vertebrate central nervous system (CNS) compartments (Ikuta and Saiga, 2007), most recent evolutionary models propose an anterior hindbrain-like identity for the neck (Hudson and Yasuo, 2021). Although the neck is specified during embryonic development and shares a close developmental lineage with differentiated larval brain neurons (Fig. 1B-D), most of it remains in an undifferentiated state until after metamorphosis (Dufour et al., 2006; Imai et al., 2009). With some rare exceptions, most differentiated neurons of the larval CNS degenerate during metamorphosis and undifferentiated neural progenitors in the neck and in other compartments survive to form the adult nervous system (Fig. 1D) (Horie et al., 2011; Hozumi et al., 2015). The signaling pathways and regulatory networks that direct neck specification, survival and differentiation remain uncharacterized.

Here, we investigate the molecular mechanisms underlying the specification and patterning of the neck. We show that the neck cells continue to divide throughout embryogenesis and the larval phase, with some derivatives differentiating into larval- and adult-specific neurons. We show that Pax2/5/8.a and Phox2 are required for the specification of neck-derived adult neurons, and that FGF signaling regulates the balance of differentiation and proliferation/survival in the neck during metamorphosis. Thus, we reveal key molecular mechanisms underlying the ability of *Ciona* to generate an entirely separate adult nervous system even as its larval nervous system is largely eliminated.

## RESULTS

### A time-series description of the ‘neck’ lineage through development and metamorphosis

**Fig. 2.**
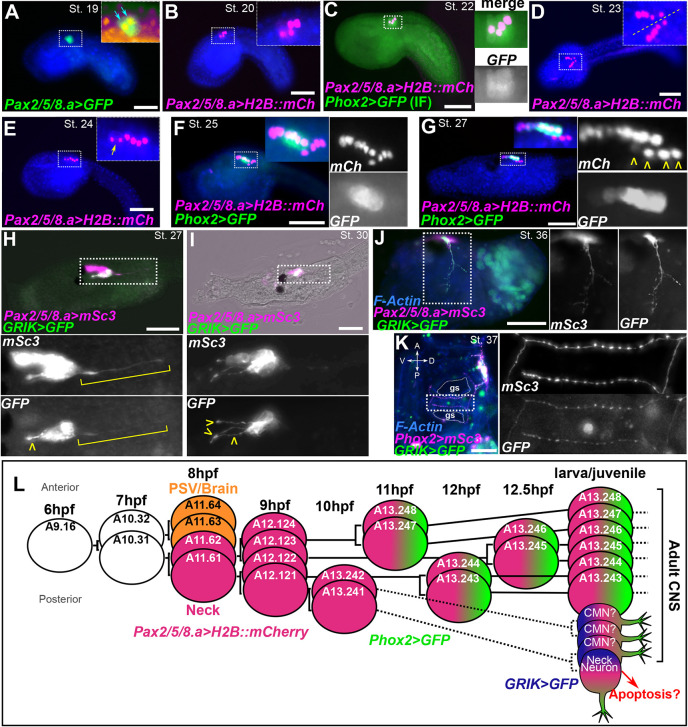
**Time-series investigation of cell division and differentiation in the neck.** (A-G) A time-series of images of *C. robusta* embryos electroporated with *Pax2/5/8.a* and *Phox2* (*C. intestinalis/Type B*) reporter plasmids and fixed at different times. Insets show higher magnification views of the areas in the dashed rectangles. Nuclei were counterstained with Hoechst 33342 (blue). Reporter genes used were H2B::mCherry (H2B::mCh) in magenta, and Unc-76::GFP (GFP) in green. Blue arrows in A indicate two *Pax2/5/8.a>Unc-76::GFP^+^* cells. (C) Nascent *Phox2(C. intestinalis)>Unc-76::GFP* expression as detected by immunofluorescent (IF) of GFP. Yellow arrow in E shows a cell undergoing mitosis. Yellow arrowheads in G show differentiating neuron nuclei separating from the rest of the neck, which remains a part of the neural tube epithelium. (H) St. 27 larva (∼17 hpf) electroporated with *Pax2/5/8.a>Unc76::mSc3* and *GRIK>Unc76::GFP* showing GFP- and mSc3-positive neurite outgrowth (yellow arrowhead) and neck neuron (NN, yellow bracket). (I) Stage 30 larva (∼24 hpf) electroporated with *Pax2/5/8.a>Unc76::mSc3* and *GRIK>Unc76::GFP* showing GFP- and mSc3-positive neurite outgrowth (yellow arrowheads). A, anterior; D, dorsal; P, posterior; V, ventral. (J) St. 36 body-axis rotating animal (∼48 hpf) electroporated with *Pax2/5/8.a>Unc76::mSc3* and *GRIK>Unc76::GFP* showing GFP- and mSc3-positive neurite outgrowth. (K) St. 37 post-metamorphic juvenile (∼72 hpf) electroporated with *Phox2>Unc76::mSc3* and *GRIK>Unc76::GFP* showing GFP- and mSc3-positive CMNs innervating the gill slits (gs). Animal was counterstained with phalloidin-Alexa Fluor 405. See Fig. S1 for a magnified view of the cerebral ganglion region and double labeling with *Pax2/5/8.a>H2B::mCherry.* (L) Proposed cell lineage and cell division timing for the early neck. Hypothesized cell divisions displayed with dashed lines. Depicted expression of *Phox2* based on direct visualization of *Phox2>GFP* reporter fluorescence and not *Phox2* transcript. All fluorescent images are of promoter-driven fluorescent proteins. In H-K, boxed areas are shown at higher magnification in associated panels. PSV, posterior sensory vesicle. Scale bars: 50 µm.

Presently, the *Ciona robusta* neck has been described as six ependymal cells per side, thought to be quiescent in nature, and a bilateral pair of differentiated neurons, the so-called ‘neck neurons’ (NNs) (Ryan et al., 2016). To establish how the neck lineage develops between early tailbud and hatched larval stage, we examined neck cell number, cell type, and morphology using *Pax2/5/8.a* (Ryan et al., 2017; Oonuma et al., 2021), *Phox2* (Dufour et al., 2006), and a novel *GRIK* fluorescent reporter at hourly intervals, in embryos raised at 20°C (Fig. 2A-K).

At the early tailbud stage [Hotta stage (St.) 19, ∼9 hours post-fertilization (hpf)], two *Pax2/5/8.a>GFP^+^* cells on either the left or the right side of the embryo could be observed, corresponding to A11.61 and A11.62 (Fig. 2A) (Ikuta and Saiga, 2007; Imai et al., 2009). These two cells continued to divide, forming a single line of four cells on either side by the mid-tailbud stage (St. 20-22, ∼9-10 hpf; Fig. 2B). By this stage, the four cells started to express *Phox2>GFP*, which was only detectable by anti-GFP immunofluorescence likely due to the low levels of mature GFP protein at this stage (Fig. 2C). Starting at St. 23 (∼11 hpf), the posterior-most cell (A12.121) divided first (Fig. 2D), followed by the anterior-most cell (A12.124) dividing around St. 24 (∼12 hpf; Fig. 2E). This brought the total number of *Pax2/5/8.a^+^* cells on either side to six. These six neck cells had not yet differentiated and maintained a typical ependymal cell morphology within the neural tube epithelium. Although the cells appeared to divide in a stereotypical order, the exact timing of their divisions was slightly variable from embryo to embryo and even between left and right sides in the same individual (Fig. S1A), as has been previously reported in the *Ciona* neural tube (Cole and Meinertzhagen, 2004).

During the late tailbud stage (St. 25, ∼14 hpf), we observed bright *Phox2>GFP* expression mostly in the middle to anterior part of the neck (Fig. 2F). Notably, *Phox2>GFP* was not visible in the posterior cells of the lineage, which appeared to delaminate from the neural tube epithelium, separating from the *Phox2^+^* cells (Fig. 2F,G). Beyond stage 26 (larval stages, ∼16 hpf onwards), additional cell divisions were seen among the *Phox2*>GFP*^+^* cells, as well as the anterior *Phox2*-negative cells. This increased the number of *Pax2/5/8.a^+^* cells to an average of 12 on each side, but the order of divisions and lineage could not be easily traced (Fig. 2G, Fig. S2B). Furthermore, *Phox2>GFP* expression expanded anteriorly in the lineage during the larval phase, but was still not detectable in the posterior cells that had delaminated from the neural tube epithelium (Fig. 2G). This *Phox2>GFP* expression in the anterior cells may have resulted from uneven proliferation of the *Phox2*^+^ cells descended from A12.123/A12.122, or from earlier transient expression in the anterior cells, which might vary based on mosaicism. The average number of *Pax2/5/8.a^+^* neck cells was quantified at each stage (Fig. S2B). We conclude that neck cells continue to divide into the larval stage, suggesting they are not quiescent.

**Fig. 3.**
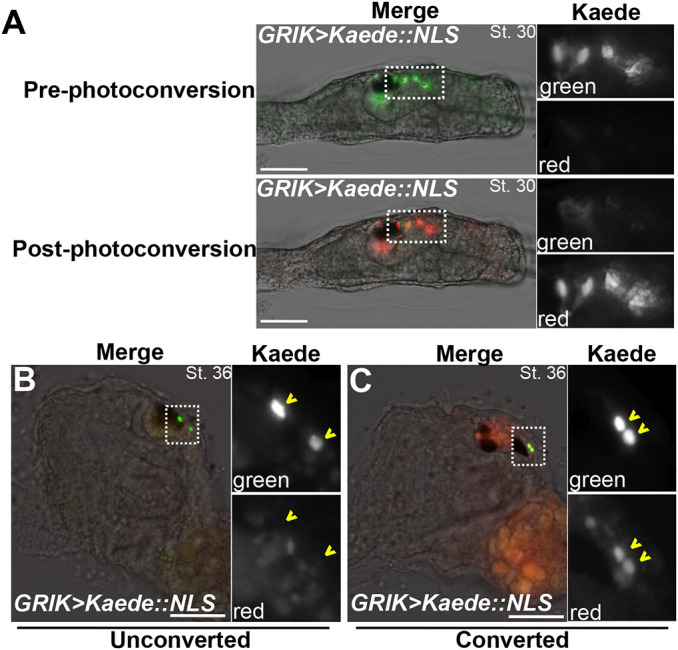
**Neck-derived GRIK^+^ cells in the larva persist through metamorphosis.** Using Kaede, an ultraviolet-sensitive photoconvertible fluorescent protein, expressed by the GRIK promoter, we traced cell lineage and survival through metamorphosis. (A) St. 30 (∼22 hpf) larva expressing the GRIK>Kaede reporter pre- and post-photoconversion. Before exposure, only green fluorescent protein was detectable in four neck-derived neurons. After exposure, most fluorescent protein was photoconverted to red. (B) St. 36 (∼44 hpf) post-metamorphic juvenile that did not undergo the photoconversion process, in which GRIK^+^ cells in the brain are only green, suggesting ongoing *GRIK>Kaede* expression post-metamorphosis. (C) St. 36 (∼44 hpf) post-metamorphic juvenile that underwent photoconversion as a larva, in which green Kaede^+^ cells have red (photoconverted) Kaede as well, confirming that these cells are derived from GRIK>Kaede::NLS^+^ cells photoconverted at the larval stage. Insets show magnified neck or brain regions, depending on animal staging, with green and red channels presented in grayscale. Scale bars: 50 µm.

At the larval stage, we first observed the differentiation of the posterior-most cell of the lineage on either side at larval stages, likely the single left/right pair of NNs previously described (Ryan et al., 2016, 2017). Shortly thereafter, we observed ascending *Pax2/5/8.a* reporter-expressing neurites extending from three cells situated just anterior to the NNs (Fig. 2H,I, Fig. S2). To examine further these different Neck-derived neurons, we generated a reporter using an intronic *cis*-regulatory element from the *Glutamate receptor ionotropic kainate* (*GRIK*) gene, expression of which had previously been detected by *in situ* hybridization in four neck cells on either side (Borba et al., 2023 preprint). In early larvae (St. 27, ∼17 hpf and St. 30, 24 hpf) we frequently observed *GRIK>GFP* signal in four cells on either side, including the NN and the three more anterior ascending neurons (Fig. 2H,I). In animals undergoing body-axis rotation (St. 36, 48 hpf), *GRIK>GFP^+^* cells appeared to continue extending their axons into the body cavity (Fig. 2J). In early post-metamorphic juveniles (St. 36, 38, ∼72-95 hpf), we observed neurons labeled by *Pax2/5/8.a* and *Phox2* reporters innervating the gill slits (Fig. S1C,D, Movie 1), similar to those reported previously (Dufour et al., 2006), and that presumably correspond to the cholinergic ciliomotor neurons (CMNs), which control branchial ciliary flow (Hozumi et al., 2015; Jokura et al., 2020). In post-metamorphic juveniles, *Phox2>mSc3*^+^ neurons innervating the gill slits were also *GRIK>GFP*^+^, supporting our model that some *GRIK>GFP*^+^ cells observed in the larva might correspond to post-metamorphic CMNs (Fig. 2K). This interpretation was further supported by photoconversion of Kaede, a photoconvertible fluorescent reporter (Ando et al., 2002). Kaede was expressed in posterior larval neck cells using the *GRIK* driver, and photoconverted before metamorphosis (Fig. 3A). Photoconverted Kaede^+^ cells were observed in the cerebral ganglion after metamorphosis, but none was seen in post-metamorphic juveniles that were not subjected to photoconverting illumination (Fig. 3B,C). Together, these observations suggest that the adult nervous system begins to differentiate before the larval phase is over. This was surprising given the traditional assumption that larval neurons are eliminated during metamorphosis, whereas adult neurons arise from set-aside, undifferentiated progenitors. Our results suggest a less clear contrast between pre- and post-metamorphic neurogenesis in tunicates. We propose an updated model of the neck cell lineage in Fig. 2L.

### Pax2/5/8 and Phox2 orthologs are required for the formation of neck-derived neurons

**Fig. 4.**
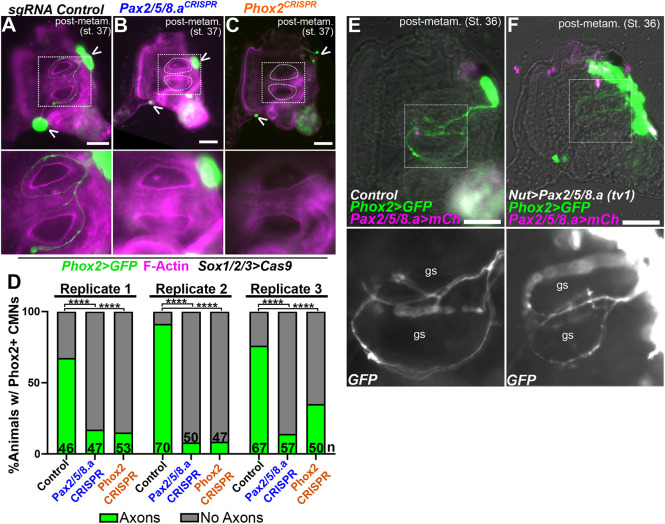
**Regulation of adult ciliomotor neuron specification by Pax2/5/8.a and Phox2.** (A) ‘Negative control’ St. 37 juvenile electroporated with *Sox1/2/3>Cas9::Geminin^N-ter^* and the *U6>Control* sgRNA expression vector, showing a normal pattern of *Phox2 (C.robusta)>Unc-76::GFP* reporter expression in neuronal cell bodies in the brain and heart (arrowheads) and axons innervating the gill slits (highlighted in the higher magnification image of the boxed area). (B) St. 37 juvenile in which neural-specific knockout of *Pax2/5/8.a* by CRISPR/Cas9 has resulted in loss of gill slit-innervating axons (highlighted in the higher magnification image of the boxed area), suggesting loss of ciliomotor neurons (CMNs). *Phox2* reporter expression in the brain (arrowheads) suggests some Phox2^+^ cells may still be specified. (C) St. 37 juvenile in which neural-specific knockout of *Phox2* has been achieved by CRISPR/Cas9, also resulting in loss of CMN axons innervating the gill slits (highlighted in the higher magnification image of the boxed area), and sparse Phox2^+^ cells remaining in the brain (open arrowheads in A-C). Counterstain in A-C was phalloidin-Alexa Fluor 647 (magenta). (D) Scoring of *Phox2^+^* CMN axon across all three conditions represented in A-C, in three independent replicates (*n*=number of individuals scored in each sample). Individuals were scored for the presence or absence of *Phox2>Unc-76::GFP* CMN axons innervating the gill slits. ****P<0.0001 (Fisher's exact test). (E) St. 36 juvenile (∼72 hpf) electroporated with *Phox2(C.robusta)>Unc-76::GFP* (green) and *Pax2/5/8.a>H2B::mCherry* (magenta nuclei), and a negative control *Nut>lacZ* plasmid. Gill slits (gs) innervated by CMNs are shown below in the higher magnification images of the boxed areas. (F) St. 36 juvenile electroporated with same reporters as in E, plus *Nut>Pax2/5/8.a (tv1)* to drive expression of Pax2/5/8.a in the entire larval neural tube. *Phox2* reporter is expanded in the resulting juveniles, although there is no noticeable increase in CMN axons innervating the gill slits. F-actin was labeled with conjugated Phalloidin; all other fluorescence is by promoter-driven fluorescent proteins. Scale bars: 50 µm.

Having established that the neck gives rise to gill slit-innervating CMNs, we next tested the roles of *Pax2/5/8.a* and *Phox2* in their specification. More specifically, we used tissue-specific CRISPR/Cas9-mediated mutagenesis to disrupt either gene in neural progenitors prior to hatching and metamorphosis. Embryos were electroporated with ‘negative control’ or gene-specific single-chain guide RNA (sgRNA) expression plasmids, *Sox1/2/3>Cas9::Geminin^N-ter^* plasmid to drive expression of Cas9 in early neural progenitors, and a *Phox2>GFP* reporter to label CMNs. The *Sox1/2/3* (also known as *SoxB1*) promoter drives Cas9 expression in the ectoderm and is pan-neuronal (Stolfi et al., 2014b). The sgRNAs were validated by Illumina sequencing of amplicons from targeted embryos (see Materials and Methods). Animals were grown to St. 37 (∼72 hpf) and imaged to assay the presence of properly differentiated CMNs. In negative control animals, *Phox2>GFP^+^* CMN axons were observed innervating gill slits in a majority of animals (Fig. 4A,D). In contrast, disrupting either *Pax2/5/8.a* or *Phox2* by CRISPR resulted in fewer gill-innervating axons, suggesting loss or incomplete differentiation of CMNs (Fig. 4B-D). Consistent with this, *Pax2/5/8.a* CRISPR also caused loss of *GRIK>GFP*^+^ neck cells in larvae (Fig. S1E,F). This suggests that Pax2/5/8.a and Phox2 are necessary for the formation of post-metamorphic CMNs.

To understand how overexpression of Pax2/5/8.a might impose a neck cell identity, we expressed *Pax2/5/8.a* (transcript variant 1, or tv1) under control of the pan-neuronal Nut promoter (*Nut>Pax2/5/8.a)* (Shimai et al., 2010). In *Nut>lacZ* negative controls, *Phox2>GFP^+^* CMNs innervate the early gill slits as expected (Fig. 4E). In animals overexpressing Pax2/5/8.a, we observed an expansion of Phox2^+^ cells throughout the juvenile brain region, but we did not observe any obvious excess of gill slit-innervating Phox2^+^ axons (Fig. 4F). Together, this suggests that Pax2/5/8.a is sufficient to activate Phox2 expression in the post-metamorphic CNS, but might not be sufficient to impart specifically a CMN-like identity.

### Characterizing the transcriptional program downstream of Pax2/5/8 by RNA sequencing

**Fig. 5.**
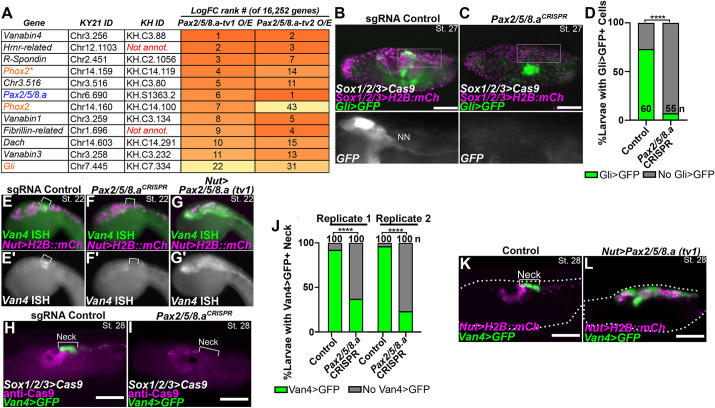
**Identification of neck-specific gene expression downstream of Pax2/5/8.a.** (A) Table of rank-ordered genes upregulated by Pax2/5/8.a overexpression (O/E) in *Ciona* embryos compared with negative control, as measured by bulk RNAseq. LogFC rank #=ranking of gene when sorting all genes by average Log2 fold-change between the negative control condition (*Nut>lacZ)* and overexpression conditions (*Nut>Pax2/5/8.a*, tv1 or tv2). See text for details. Kyoto 2021 (KY21) and KyotoHoya (KH) gene identification numbers are given for each gene. Genes in orange are previously known Pax2/5/8.a targets, identified by morpholino knockdown (Imai et al., 2009). Asterisk denotes C-terminal fragment gene model of *Phox2* (see discussion of Fig. S4 data in the Results for details). Differential expression of 16,252 gene models was analyzed in total. (B) Negative control animal showing *Gli>Unc76:GFP (Gli>GFP; green)* reporter gene expression in the neck and neck neuron (NN). (C) CRISPR/Cas9-mediated mutagenesis of *Pax2/5/8.a* results in loss of *Gli>GFP* reporter gene expression in the neck. *Sox1/2/3* promoter was used to drive expression of Cas9::Geminin^N-ter^ and H2B::mCherry (magenta nuclei) in the ectoderm, including the nervous system. In B,C, lower panels show high-magnifications of the boxed regions above. (D) Scoring of *Gli^+^* neck cells for the samples represented in B,C. Individuals were scored for the presence or absence of *Gli>GFP* cells in the neck region, posterior to the ocellus and otolith. (E) *In situ* mRNA hybridization for the Pax2/5/8.a downstream gene *Vanabin4* (*Van4*) in a negative control embryo, showing specific expression in the neck (bracket) at St. 22 (10 hpf). (F) *Pax2/5/8.a* knockout by CRISPR/Cas9 in the nervous system (using *Sox1/2/3>Cas9*) results in loss of *Van4* mRNA *in situ* signal in the neck. (G) Overexpression of *Pax2/5/8.a* (tv1) throughout the neural tube (*Nut>Pax2/5/8.a tv1*) results in widespread, ectopic expression of *Van4*, confirming this gene as being downstream of Pax2/5/8.a as indicated by RNAseq. All embryos were electroporated with *Nut>H2B::mCherry* (magenta nuclei). (E′-G′) Single channel grayscale images of *Van4* expression. (H) *Van4>Unc-76::GFP* reporter expression (green) also specifically labels the neck (bracket) in St. 28 negative control larvae (∼20 hpf). (I) Knockout of *Pax2/5/8.a* in the nervous system results in loss of *Van4* reporter expression. In H,I, Cas9 protein is visualized by immunostaining (magenta). (J) Scoring of *Van4>Unc-76::GFP* reporter expression in the *Pax2/5/8.a* CRISPR larvae represented in H,I, showing a dramatic reduction in the frequency of *Van4^+^* larvae in *Pax2/5/8.a* CRISPR larvae compared with negative control larvae, in two independent replicates. (K) St. 28 larva electroporated with *Nut>H2B::mCherry* (magenta nuclei), *Van4>Unc-76::GFP* (green) reporters, and *Nut>lacZ* control. (L) Larva electroporated with same reporters as in K, together with *Nut>Pax2/5/8.a tv1*, showing expansion of *Van4* reporter expression throughout the neural tube. In K,L, dotted lines delineate the larval body. In D and J, *n*=number of individuals scored in each sample. Scale bars: 50 µm. ****P<0.0001 (Fisher's exact test).

In order to confirm the role of Pax2/5/8.a as a regulator of neck identity and transcriptional programs, we used bulk RNA sequencing (RNAseq) to measure transcriptome changes elicited by ectopic Pax2/5/8.a overexpression. We used the *Nut* promoter (Shimai et al., 2010) to overexpress both isoforms of *Pax2/5/8.a* (transcript variants ‘tv1’ and ‘tv2’) throughout the neural tube, and compared whole-embryo transcriptomes of Pax2/5/8.a-overexpression and negative control (no overexpression) embryos at 10 hpf. Differential gene expression analysis revealed the enrichment, or depletion, of transcripts upon overexpression of either Pax2/5/8.a variant (Fig. 5A, Table S1). Previously known neck markers and Pax2/5/8.a targets (e.g. *Phox2*, *Gli*, *Eph.c*, *FGF9/16/*20) were among the top 600 upregulated genes (out of ∼16,000), with some being considerably higher in ranking (e.g. *Phox2*). In contrast, several known markers of the brain and motor ganglion (MG) were among the top 50 genes most downregulated by Pax2/5/8.a (e.g. *Pax6*, *Nk6*, *Neurogenin*, *BCO*)*.* Correlation between differential gene expression values elicited by the two isoforms was modestly high (Pearson correlation r=0.70) (Fig. S3), suggesting high reproducibility and specificity of the downstream effects of Pax2/5/8.a. Interestingly, despite the neck-specific expression of the *GRIK>GFP* reporter and its loss in *Pax2/5/8.a* CRISPR larvae, *GRIK* was not significantly upregulated by *Pax2/5/8.a* in our RNAseq experiment, suggesting that Pax2/5/8.a might be necessary but not sufficient for *GRIK* expression, or a mechanism exists that limits *GRIK* activation prior to hatching. Similarly, *Hox1* expression in the neck appeared to be independent of Pax2/5/8.a, consistent with previous studies that revealed its activation by retinoic acid signaling instead (Imai et al., 2009).

One gene previously known to be downstream of Pax2/5/8.a, *Gli* (Imai et al., 2009), was among the top 31 upregulated genes using either Pax2/5/8.a isoform (Fig. 5A). Indeed, CRISPR/Cas9-mediated knockout of *Pax2/5/8.a* largely abolished *Gli>GFP* reporter expression in the neck, confirming its regulation by Pax2/5/8.a (Fig. 5B-D). Following the same approach, we identified a previously unknown target of Pax2/5/8.a, the gene *Vanabin4* (*Van4*, gene IDs *KH.C3.88/KY21.Chr3.256*). *Van4* encodes a tunicate-specific vanadium-binding protein and was the second most upregulated gene by both Pax2/5/8.a isoforms (Fig. 5A). *In situ* mRNA hybridization revealed expression of *Van4* specifically in the neck (Fig. 5E,E′). *Van4* expression was lost upon *Pax2/5/8.a* CRISPR knockout (Fig. 5F,F′) and expanded throughout the neural tube upon overexpression of Pax2/5/8.a (Fig. 5G,G′). *Van4>GFP* reporter expression in the neck was similarly lost upon *Pax2/5/8.a* CRISPR knockout (Fig. 5H-J) and reporter expression was expanded across the neural tube upon overexpression of Pax2/5/8.a (Fig. 5K,L). Taken together, these data reveal a transcriptional program for neck specification downstream of Pax2/5/8.a*.*

### The *C. robusta Phox2* gene extends more in the 3′ direction than previously thought

During analysis of RNAseq data, we observed upregulation of *Phox2* (*KY21.Chr14.160*) and the neighboring gene *KY21.Chr14.159* by Pax2/5/8.a overexpression. On closer inspection, we found that RNAseq reads spanned exons covering both gene models, and further identified two cryptic exons (exon 1 and exon 5) (Fig. S4A,B). Our corrected gene model (Fig. S4C) was supported by cloning and sequencing a full-length *Phox2* cDNA spanning all seven exons (Fig. S4D), and by protein sequence alignment with predicted Phox2 sequences from other tunicate species (Fig. S4E). Finally, mRNA *in situ* hybridization using probes designed for both *Chr14.160* and *Chr14.159* gene models revealed identical expression patterns, further supporting the finding that these are the same gene (Fig. S4F,G). This could explain why enrichment of *Phox2* might go undetected in certain RNAseq data when using 3′-biased sequencing. The function of the extended C terminus (which is divergent relative to that of vertebrate Phox2 family members) is unknown but will likely be important for future studies on transcriptional regulation by Phox2.

### Characterization of neck cell transcriptomes in single-cell RNAseq data

To characterize further the molecular profiles of neck cells, we re-analyzed published whole-embryo single-cell RNAseq (scRNAseq) data obtained at the mid-tailbud II stage (Cao et al., 2019). This revealed a single cluster enriched for *Pax2/5/8.a* reads (cluster 25; Figs S5A, S6A,B, Table S2). Re-clustering performed only on cluster 25 cells revealed two distinct subclusters, with *Pax2/5/8.a* enriched in one subcluster (subcluster 0) and depleted in the other (subcluster 1) (Figs S5B, S6C). Double *in situ* hybridization using probes for *Pax2/5/8.a* and a top subcluster 1 marker, *Crls1* (*KH.C11.724*), confirmed that subcluster 0 represents the neck, whereas subcluster 1 appears to represent larval brain neurons and photoreceptors just anterior to the neck (Fig. S5C). Other markers enriched in subcluster 0 cells further confirmed their identity as neck cells, showing substantial overlap with the top genes upregulated by Pax2/5/8.a overexpression as measured by our bulk RNAseq experiment above (Fig. S5D,E, Table S3). Correlation between enrichment/depletion in the neck by scRNAseq and average upregulation/downregulation by Pax2/5/8.a was modest (Pearson correlation=0.43; Spearman's rank correlation=0.46) (Table S4). We observed notable exceptions, such as *Hox1*, which is highly expressed in the neck but was not upregulated by Pax2/5/8.a (Fig. S5D,E). This suggests that Pax2/5/8.a is not sufficient to activate the entirety of the transcriptional program of the neck, and that some important neck regulators might be expressed in parallel to, not downstream of, Pax2/5/8.a. In contrast, the top markers enriched in the ‘brain’ subcluster (subcluster 1) were among those transcripts most highly depleted by Pax2/5/8.a overexpression, suggesting that Pax2/5/8.a is also instrumental for repressing larval brain neuron/photoreceptor identity.

As expected, *Phox2* expression (taken as the expression of the more 3′ KH.C14.119/KY21.Chr14.159 gene model due to the 3′ bias of 10x Genomics system) was unevenly distributed within subcluster 0. *Phox2* was expressed more highly in one half of the subcluster than in the other half (Fig. S5F), recapitulating its expression in a subset of neck cells as seen by *Phox2>GFP* expression. Additional genes showed a similar distribution in the neck subcluster (e.g. *Rspo3*, *Tesk*; Figs S5F, S6D), further hinting at a distinction between proliferating/undifferentiated cells in the anterior and differentiating neurons in the posterior.

### Ephrin/Eph and FGF signaling regulate the balance between proliferation and differentiation

**Fig. 6.**
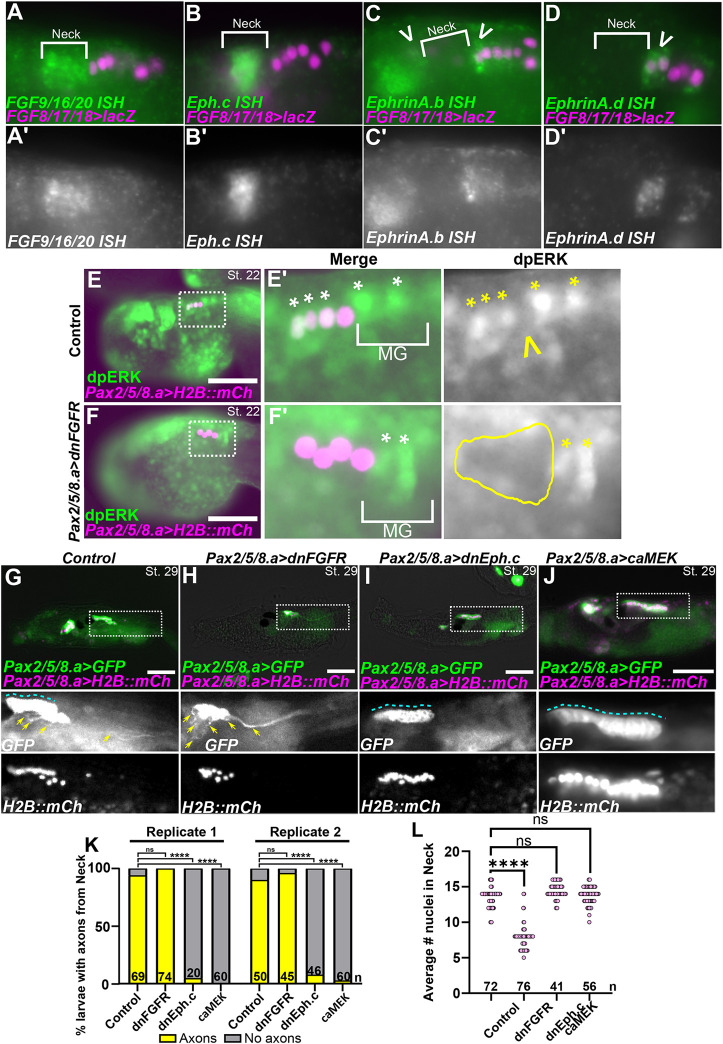
**Ephrin/Eph and FGF/MEK signaling pathways regulate differentiation in the neck.** (A) *In situ* hybridization (ISH) for *FGF9/16/20* (green), showing expression in the neck at St. 22. (B) ISH for *Eph.c* (green), showing expression in the neck (St. 22), more strongly in middle cells. (C) ISH for *EphrinA.b* (green), showing expression in the anterior cells of the A9.30 lineage as well as the brain region (arrowheads), both abutting the limits of the neck. (D) ISH for *EphrinA.d* (green), showing expression in the anterior cells of the A9.30 lineage (arrowhead). In A-D, the neighboring A9.30 lineage is marked by *FGF8/17/18>lacZ,* revealed by β-galactosidase immunostaining (magenta nuclei). (A′-D′) Single-channel grayscale images of ISH targets. (E,E′) Control St. 22 embryo (∼9 hpf) expressing *Pax2/5/8.a>H2B::mCh* and stained for doubly phosphorylated ERK (dpERK). The anterior three cells are dpERK^+^ whereas the posterior cell is dpERK^−^ (indicated by yellow open arrowhead). (F,F′) Experimental animals electroporated with *Pax2/5/8.a>dnFGFR*, which suppresses dpERK only in neck cells. White and yellow asterisks identify dpERK^+^ cells. The yellow outline highlights the neck region with absence of dpERK signal. Quantitative analysis of dpERK staining is shown in Fig. S7. MG, motor ganglion. (G) St. 29 larvae (∼21 hpf) electroporated with *Pax2/5/8.a>lacZ* control, *Pax2/5/8.a>Unc-76::GFP* (green) and *Pax2/5/8.a>H2B::mCherry* (magenta nuclei), showing the neck giving rise to both undifferentiated cells forming an epithelium in the neural tube (blue dashed line) as well as differentiating neurons and extending axons (yellow arrows). (H) Larva expressing *Pax2/5/8.a>dnFGFR*, showing loss of epithelial structure in the neck and supernumerary *Pax2/5/8.a^+^* axons (yellow arrows). (I) Larva expressing *Pax2/5/8.a>dnEph.c,* showing expansion of the undifferentiated neuroepithelium and loss of axons emanating from the neck. (J) Larva expressing *Pax2/5/8.a>caMEK*, which expanded the neuroepithelial state and suppressed neuronal differentiation and axon growth. In G-J, boxed areas are shown at higher magnification below. (K) Scoring of larvae represented in G-J, showing almost complete loss of neck-derived axons in larvae expressing dnEph.c or caMEK in the neck, but not dnFGFR across two replicates. (L) Plot showing the average number of *Pax2/5/8^+^* nuclei counted in the larvae represented in G-J, showing significantly fewer nuclei in the dnFGFR condition. In K and L, *n*=number of individuals scored in each sample. Scale bars: 50 µm. ****P<0.0001 (Fisher's exact test). ns, not significant (*P*>0.05).

FGF/MAPK signaling plays numerous roles in the development of the *Ciona* larval nervous system (Davidson et al., 2006; Picco et al., 2007; Stolfi et al., 2011; Haupaix et al., 2013; Razy-Krajka et al., 2018). In fact, FGF8/17/18 from the neighboring A9.30 cell lineage is required for expression of *Pax2/5/8.a*, which in turn activates expression of *FGF9/16/20* in the neck (Fig. 6A,A′) (Imai et al., 2002, 2009). Furthermore, Ephrin/Eph signaling antagonizes FGF/MAPK signaling as a spatial cue for FGF/MAPK-dependent cell fate choices in the nervous system, including the MG, in which downregulation of FGF/MEK promotes cell cycle exit and neuronal differentiation (Stolfi et al., 2011; Haupaix et al., 2013). By *in situ* hybridization, we confirmed the expression of *FGF9/16/20* and *Eph.c* (formerly *Eph3*) in the neck (Fig. 6A-B′), and *EphrinA.b* and *EphrinA.d* (Fig. 6C-D′) in the cells of MG just posterior to the neck (Imai et al., 2009). Immunostaining for doubly phosphorylated ERK (dpERK) showed that anterior *Pax2/5/8.a>H2b::mCh*^+^ cells were dpERK^+^, whereas the posterior-most fourth cell in contact with Ephrin^+^ MG cells was not labeled with dpERK antibody (Fig. 6E,E′, Fig. S7). As expected, dpERK signal was abolished in the neck by forced expression of a dominant-negative FGF receptor (dnFGFR) (Fig. 6F,F′). Taken together, these data suggested that FGF and Ephrin/Eph signaling might be key to regulating cell fate and neuronal differentiation in the neck as well.

To test the role of FGF and Ephrin signaling in the neck, we used the *Pax2/5/8.a* promoter to overexpress dnFGFR, dominant negative Eph.c receptor (dnEph.c), or a constitutively active form of the MAPK kinase MEK (MEK^S220E,S216D^, also called caMEK) (Davidson et al., 2006; Picco et al., 2007; Shi and Levine, 2008; Razy-Krajka et al., 2018). We examined the neck at the late swimming larval stage (St. 29), when there are approximately eight anterior putatively undifferentiated, neuroepithelial cells and approximately four posterior, differentiating neurons in control larvae (Fig. 6G,K,L). In larvae expressing the dnFGFR, we observed fewer cells overall in the neck, and undifferentiated cells appeared to be replaced by supernumerary neurons, as evidenced by loss of epithelial organization and excess axon outgrowth (Fig. 6H,K,L). This contrasted starkly with larvae expressing the dnEph.c, in which we observed a near-complete loss of differentiated neurons, most clearly evidenced by a distinct absence of the posterior NN axon extending towards the tail (Fig. 6I,K,L). Instead, the entire neck took on the morphology of undifferentiated neural precursors in a tightly packed neuroepithelium. This was phenocopied by caMEK, which constitutively activates the FGF/MAPK pathway in all cells (Fig. 6J-L). We observed these phenotypes consistently across two biological replicates (Fig. 6K,L). We also examined the expression of *Phox2>GFP* in the neck, which is normally expressed by the ependymal-like cells in the middle part of the neck (Fig. 2C, Fig. S5F). As expected, we observed a loss of Phox2^+^ neck cells in larvae electroporated with *Pax2/5/8.a>dnFGFR* (Fig. S8A,B,E). However, neither *Pax2/5/8.a>dnEph.c* nor *Pax2/5/8.a>caMEK* abolished *Phox2>GFP* expression but rather made its spatial distribution within the neck inconsistent (Fig. S8C-E). This suggests that sustained FGF/MEK signaling is permissive but not sufficient for *Phox2* activation in the neck. Taken together, these results suggest that Ephrin/Eph-mediated suppression of FGF/MAPK signaling is sufficient and necessary for neuronal differentiation in the neck, and that sustained FGF/MAPK signaling promotes an undifferentiated, neuroepithelial state instead.

Because our data had revealed upregulation of genes encoding the BMP antagonist Noggin and the Hedgehog (Hh) pathway effector Gli, downstream of Pax2/5/8.a (Fig. 4, Table S4), we also tested the effects of perturbing these pathways. We did not observe any obvious effects on neck morphogenesis upon overexpression of dominant-negative or constitutive BMP receptors (Fig. S9A-D) or CRISPR/Cas9-mediated knockout of *Gli* (Fig. S9E-G). Although these results do not rule out a role for BMP and/ Hh signaling pathways in the development of the neck or neck-derived neurons, we were unable to find any evidence that they impact the balance of differentiation and proliferation like Ephrin and FGF do.

**Fig. 7.**
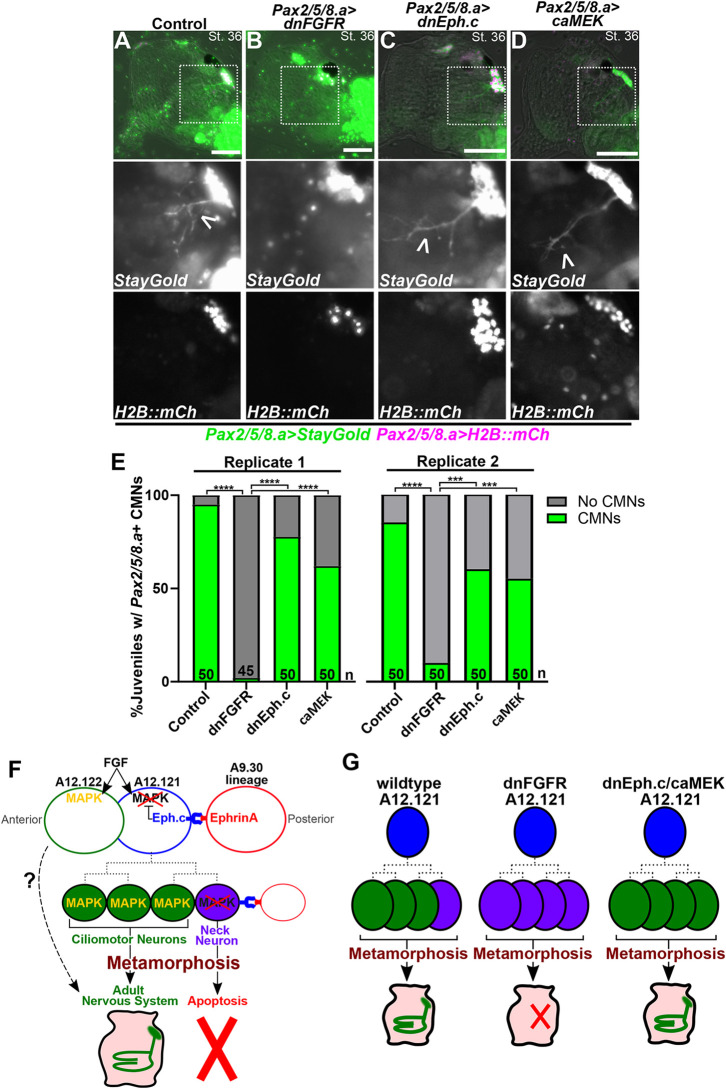
**FGF signaling is required for survival of CMN precursors through metamorphosis.** (A-D) St. 36 juveniles (∼72 hpf) electroporated with *Pax2/5/8.a>Unc-76::StayGold* (*Pax2/5/8.a>StayGold*; green) and *Pax2/5/8.a>H2B::mCherry* (magenta), showing growing ciliomotor (CMN) axons (arrowheads) in the process of innervating the gill slits. (A) Negative controls expressing *Pax2/5/8.a>lacZ* have normal CMNs. (B) *Pax2/5/8.a>dnFGFR* eliminates CMN axons, suggesting that maintenance of FGF/MAPK signaling is important for specification of CMNs and survival through metamorphosis. (C) Juveniles expressing dnEph.c still give rise to CMNs (arrowhead) during metamorphosis, indicating an ability to recover from earlier suppression of differentiation in the larva (see Fig. 6). (D) Juveniles expressing caMEK also recover, growing CMN axons (arrowhead) during metamorphosis. In A-D, boxed areas are shown at higher magnification below. (E) Scoring of juveniles represented in A-D, showing near total loss of CMNs in juveniles derived from larvae expressing dnFGFR in the neck (two replicates). *n*=number of individuals scored in each sample. ***P<0.001, ****P<0.0001 (Fisher's exact test). (F) Diagram explaining the model for Ephrin/Eph-mediated suppression of FGF/MAPK signaling in the posterior-most cell of the neck, which becomes the neck neuron (NN), and hypothesized cell lineage from A12.121 (dashed lines). More anterior cells escape Ephrin signaling at different times, giving rise to precursors of the adult nervous system including CMNs, whereas the NN does not appear to survive metamorphosis. The uncertain contribution of A12.122 derivatives to the adult nervous system is indicated by the dashed arrow. (G) Diagram explaining the different manipulations tested and their effects on CMN formation in juveniles. In wild-type animals, the NN (purple) does not survive metamorphosis, and the three anterior CMN precursors differentiate during late larval/early juvenile stages. In *Pax2/5/8.a>dnFGFR-*electroporated animals, the entire neck is converted to NN-like neurons, which are all eliminated during metamorphosis. In individuals electroporated with *Pax2/5/8.a>dnEph.c* or *Pax2/5/8.a>caMEK*, sustained FGF signaling temporarily suppresses neuronal differentiation in the neck during larval stages, but the cells eventually ‘recover’ during metamorphosis to give rise to CMNs. Scale bars: 50 µm.

Finally, because disruption of Ephrin/FGF/MAPK signaling significantly impacted cell proliferation and differentiation in larvae, we examined how these perturbations affected the development of CMNs in post-metamorphic juveniles. We observed a loss of *Pax2/5/8.a>GFP*^+^ CMN axons in juveniles that had expressed the dnFGFR receptor in the neck at the larval stage (Fig. 7A,B,E), but not in animals that expressed dnEph.c or caMEK (Fig. 7C-E). We conclude that the supernumerary, differentiating neurons seen in the larvae generated by *Pax2/5/8.a>dnFGFR* do not survive metamorphosis, failing to give rise to fully differentiated CMNs in the juvenile. In contrast, undifferentiated neuroepithelial cells elicited by dnEph.c or caMEK overexpression still retain the potential to differentiate into CMNs after metamorphosis. Taken together, these data suggest that the balance between neuronal differentiation in the larva and survival of neuronal precursors set aside for the adult CNS likely depends on a careful balance of FGF signaling prior to metamorphosis (Fig. 7F,G).

## DISCUSSION

We have described the development of the *Ciona* ‘neck’ through metamorphosis and post-metamorphic neuronal differentiation. Despite reports of neck cells being quiescent in the larva, we observe ongoing proliferation and precocious adult neuron differentiation throughout the larval stage. We also show that the transcription factors *Pax2/5/8.a* and *Phox2* are essential for the formation of adult (post-metamorphic) CNS neurons. Through RNAseq we have identified the neck transcriptional program downstream of *Pax2/5/8.a,* revealing candidate targets of Pax2/5/8.a that might be important for post-metamorphic neurodevelopment. Finally, although we did not identify an obvious role for either BMP or Hedgehog pathways, we establish that Ephrin and FGF signaling are responsible for patterning the neck into differentiated and undifferentiated compartments, with serious consequences for adult CNS formation if disturbed.

Across many species, *Pax2*/5/8 homologs are midbrain/hindbrain regulators, suggesting they are part of an ancestral gene regulatory network (Bridi et al., 2020; Schuster and Hirth, 2023). In vertebrates, Phox2 proteins regulate the development of various motor neurons of the head, which are also cholinergic (Pattyn et al., 2000; Mazzoni et al., 2013; Curto et al., 2015). For instance, Phox2a (along with Pax2/5) establishes oculomotor and trochlear cranial motor neurons at the isthmic organizer boundary between the midbrain and hindbrain (Deng et al., 2011; Fritzsch, 2024), and loss of *Phox2b* ablates cranial branchial neurons and visceral motor neurons of the hindbrain in mouse (Pattyn et al., 1999, 2000). Also in mouse, Phox2a promotes cranial motor neuron fate over spinal cord motor neuron fate (Mazzoni et al., 2013). In *Ciona*, *Pax2/5/8.a^+^/Phox2^+^* neck cells give rise to post-metamorphic neurons that innervate pharyngeal gill slits and possibly branchiomeric siphon muscles of the adult later on (Wada et al., 1998; Dufour et al., 2006; Hozumi et al., 2015). Here, we show that *Pax2/5/8.a* and *Phox2* are required for the formation of a subset of these neck-derived cholinergic CMNs*.* In contrast, *Ciona Phox2* is not expressed in larval motor neurons, which innervate (paraxial) muscles of the tail, like vertebrate spinal cord motor neurons. It is unclear whether the neck and/or Pax2/5/8^+^/Phox2^+^ cells also give rise to additional neuron types of the juvenile/adult. Taken together, these findings suggest that a conserved transcriptional program for cholinergic branchiomeric efferent specification and differentiation might have evolved in the last common ancestor of tunicates and vertebrates, predating the origin of cranial nerves and jaw muscles.

Like many tunicates, *Ciona* have a biphasic lifecycle; a motile, non-feeding larval stage and a sessile, filter-feeding juvenile/adult stage. It has been traditionally assumed that the development of the larval and adult nervous systems of *Ciona* are neatly separated by metamorphosis. However, our observations suggest that the larval–adult boundary of the CNS might not be as sharp. Instead, we observe delamination of multiple *GRIK^+^* cells in the posterior neck that project axons ventrally and anteriorly during the late larval stage (Fig. 2I), several hours before tail absorption and body axis rotation (Hotta et al., 2020). By following cells using a photoconvertible Kaede reporter, we find evidence that these larval *GRIK^+^* cells survive metamorphosis, suggesting they might represent the earliest-differentiating CMNs of the juvenile. In juveniles/adults, CMNs have been shown to use cholinergic neurotransmission to regulate ciliary flow in the branchial epithelium, which generates the water flow that brings food particles into the mouth (Petersen et al., 1999; Jokura et al., 2020). Therefore, the early, pre-metamorphic differentiation of these neurons may be key for the rapid onset of feeding and further growth after metamorphosis. This is consistent with the ‘anticipatory development’ of post-metamorphic structures seen in diverse tunicate larvae (Jacobs et al., 2008). Unfortunately, without an axon-targeted Kaede fusion available we were unable to verify the morphology of photoconverted cells to confirm their identity as CMNs. Highly stable axon-targeted Kaede fusion proteins will have to be developed and adapted to *Ciona* to fully address this question.

Although *Phox2* and *GRIK* show mutually exclusive expression domains at embryonic and larval stages, their reporters are co-expressed in post-metamorphic CMNs. Based on our interpretations, we believe *Phox2* is activated *de novo* in previously *Phox2-*negative, *GRIK^+^* larval cells (descended from A12.121) that give rise to early-differentiating CMNs. However, we cannot rule out that additional CMNs derive from *Phox2^+^, GRIK-*negative larval cells well after metamorphosis. As the branchial basket continues to grow, it will likely require additional innervation by Phox2^+^/GRIK^+^ CMNs. Whether this requires ongoing neurogenesis of neck-derived Phox2^+^/GRIK^+^ cells is not known, but is reasonable to assume that this may be the case. Furthermore, it is possible that there is developmental plasticity depending on the timing of larval settlement and/or rate of post-settlement growth. For instance, early-differentiating CMNs might be readily incorporated into the juvenile CNS when settlement and metamorphosis proceed rapidly, but they might not survive in cases of delayed metamorphosis. In the latter case, a pool of undifferentiated CMN progenitors might be relied upon to form CMNs when post-metamorphic growth resumes.

In addition to post-metamorphic CMNs, the neck also gives rise to the narrowly defined NNs, which here we show is the posterior-most cell of the neck lineage on either side of the larva, forming a bilaterally symmetric left-right pair of neurons. The NNs have been previously described at the early larval stage in the *C. intestinalis* whole-larva connectome studies (Ryan et al., 2016; Ryan and Meinertzhagen, 2019). The function of the NNs remains unknown, but the connectome described them as receiving synaptic inputs from ascending MG interneurons (AMG neurons) and synapsing primarily onto the basement membrane (Ryan et al., 2016). However, NNs might not be fully differentiated in early larvae, and it is possible they form connections later in larval development. Indeed, we noticed that NN axons continue to extend posteriorly during the larval phase and appear to exit the trunk and enter the proximal portion of the tail prior to larval settlement. Alternatively, the NNs might not serve a function other than to prevent nascent CMN axons from projecting posteriorly during the larval phase. This ‘roadblock’ effect is observed when MG neurons are ‘twinned’ under certain genetic perturbation conditions, in which ectopic neurons project anteriorly instead of posteriorly into the tail (Stolfi et al., 2011). Regardless of function, the NNs likely do not survive metamorphosis as we rarely observed them in juveniles. Interestingly, *Pax2/5/8.a>dnFGFR* converted the entire neck lineage into neurons that did not appear to survive metamorphosis (Fig. 7B). Because of this, we believe that the supernumerary neurons generated by dnFGFR represent NN-like neurons that do not persist to the adult stage. Although the supernumerary axons in the dnFGFR condition did not project towards the tail like the NNs, this may be due to disrupted neuronal polarity mechanisms. Consistent with this hypothesis, we have previously observed supernumerary MG neurons aberrantly project away from the tail (Stolfi et al., 2011). Future studies aim to identify regulatory differences between CMNs and NNs and to investigate what factors determine whether they survive or perish despite their shared lineage history. For instance, it is not yet clear whether sustained FGF signaling in neural progenitors is inherently pro-survival, or if FGF downregulation in the neck is simply a molecular switch for the specification of NNs, which may be pre-programmed to degenerate during metamorphosis.

It is possible that cell survival factors act downstream of *Pax2/5/8.a*, including those we identified here using RNAseq. One interesting candidate is *Vanabin4* (*Van4*), which encodes a vanadium-binding protein and was the top Pax2/5/8.a target by RNAseq. The biological function of vanadium remains elusive, and it is unclear what the role of Van4 might be in the neck (Ueki et al., 2015). Many tunicates accumulate high levels of vanadium, but not all species have vanabins and their purpose remains unknown (Ueki et al., 2003). One theory is that adults accumulate vanadium to deter predation (Odate and Pawlik, 2007). It is possible that vanadium provides a protective effect against oxidative stress (Tripathi et al., 2018), which would promote survival during metamorphosis. Future studies will examine the role of vanabins in *Ciona* and other marine organisms that accumulate vanadium (Thompson et al., 2018; Mendonca et al., 2024).

## MATERIALS AND METHODS

### *Ciona* handling, fixation, staining and imaging

Adult *Ciona robusta* (*intestinalis* Type A) were collected from San Diego, CA (M-REP). Dechorionated zygotes were generated and electroporated as previously described (Christiaen et al., 2009a,b). Embryos were raised in artificial sea water at 20°C. Animals raised beyond 24 hpf were moved to new agarose-coated plates with fresh artificial sea water containing 1.0% Penicillin-Streptomycin (Gibco) daily. Post-metamorphic animals were paralyzed with L-menthol prior to fixation, as previously described (Osugi et al., 2020). Staging is based on the TUNICANATO database (Hotta et al., 2020). Cell lineage nomenclature is based on Conklin (Conklin, 1905; Nicol and Meinertzhagen, 1991). All sequences of plasmids, probes and sgRNAs not previously published can be found in the supplementary Materials and Methods.

Sample processing for fluorescence and immunostaining were performed as previously described (Beh et al., 2007; Ikuta and Saiga, 2007; Stolfi et al., 2011). Unc-76-tagged GFP and mCherry (Dynes and Ngai, 1998) were used to improve labeling of cell bodies and axons, instead of GFP/mCherry alone. An Unc-76-tagged StayGold green fluorescent protein was also designed and used for its improved signal longevity (Hirano et al., 2022). Immunolabeling of Cas9 protein by monoclonal mouse anti-Cas9 antibody at 1:500 (4G10; Diagenode) and GFP protein by mouse anti-GFP antibody at 1:500 (Supply Solutions Roche, clones 7.1 and 13.1) was performed with blocking in PBS Super Block (37580; Thermo Fisher Scientific) and visualized with goat anti-mouse Alexa Fluor 568 or goat anti-mouse Alexa Fluor 488 (Thermo Fisher Scientific). Detection of MAP kinase pathway activity with dpERK antibody (1:500; Sigma-Aldrich, M9692) was performed as previously described (Stolfi et al., 2011, 2013). Juvenile animals were stained with phalloidin-Alexa Fluor 405 or 647 (Thermo Fisher Scientific; A30104, A22287) to visualize F-actin.

*In situ* hybridization coupled to immunostaining was performed as previously described (Beh et al., 2007), using TSA Plus amplification kits (Akoya Biosciences) and mouse anti-β-galactosidase (Promega, Z378; 1:1000) or rabbit anti-mCherry (BioVision, accession number ACY24904; 1:500) primary antibodies. Two-color (fluorescein and Cy3) double *in situ* hybridization was performed using TSA Plus amplification kits as previously described (Ikuta and Saiga, 2007; Stolfi et al., 2011). Probe template sequences can be found in the supplementary Materials and Methods.

In Kaede (Ando et al., 2002) photoconversion experiments, animals were electroporated with a plasmid using the *GRIK cis*-regulatory sequences to drive expression of Kaede fused to a nuclear localization sequence (Kaede::NLS) (Razy-Krajka et al., 2014). At 22 hpf, animals were transferred to an uncoated 10 cm plastic dish in artificial sea water (Instant Ocean Sea Salt). Half of all animals immediately underwent the photoconversion process and the other half were returned to the incubator. The Kaede construct was photoconverted using 400 nM light from an optoWELL (Opto Biolabs) at 100% intensity for 35 min. After photoconversion, animals were replaced in the incubator until post-metamorphic fixation at 44 hpf. Juveniles were fixed as previously described.

All standard images were captured on a Leica epifluorescence compound microscope. Confocal images were captured on a Nikon AX R with NSPARC and processed in ImageJ (1.54d). Phenotypes were quantified on a Leica DMi8 or DMIL LED inverted epifluorescence microscope. Biological replicates are presented in side-by-side graphs generated in Prism (9.5.1). Cell count data were analyzed by one-way ANOVA and Dunnett's test of multiple comparisons. Phenotypic data were analyzed by Fisher's exact test comparing each experimental group to the control. Significance was reported when appropriate with a minimum threshold of *P*<0.05. Fluorescence profiles and region of interest intensity of anti-dpERK-stained embryos was quantified in ImageJ and analyzed in Prism.

### CRISPR/Cas9 sgRNA design and validation

sgRNAs were designed using CRISPOR (http://crispor.tefor.net/) and vectors were constructed as previously described (Haeussler et al., 2016; Gandhi et al., 2018) or custom synthesized and cloned (Twist Bioscience). Individual sgRNA vectors were validated *in vivo* as previously described (Johnson et al., 2024) with a ubiquitous *Eef1a −1955/-1>Cas9* or *Eef1a −1955/-1>Cas9::Geminin* (Stolfi et al., 2014a; Johnson et al., 2024). Genomic DNA was isolated using QiaAMP Micro extraction kit (QIAGEN), targeted regions amplified by PCR using AccuPrime Pfx (Thermo Fisher Scientific), and PCR products purified using the QiaQuick PCR Purification kit (QIAGEN) following the published protocol (Johnson et al., 2024). Samples were sequenced using commercial Illumina-based amplicon sequencing (Amplicon-EZ; Azenta). Sequence data were analyzed by Azenta, and sgRNA efficiency was determined by the percentage of sequences containing insertions and/or deletions (indels) in and around the sgRNA target site. Data on sgRNA efficiency is provided in Fig. S10. Details of the electroporation mixes can be found in the supplementary Material and Methods. Sequences for sgRNAs and PCR primers are provided in the Table S5; gene model nomenclature can be found in Table S6.

### Bulk RNAseq

Fertilized zygotes were prepared and electroporated as described above. Zygotes were electroporated with DNA plasmids using a pan-neuronal driver *Nut −1155/-1* (Shimai et al., 2010*;* Johnson et al., 2024): *Nut-1>LacZ Control*, *Nut-1>Pax2/5/8.a transcript variant 1* (‘tv1’, transcript model *KY21.Chr6.690.v2.SL1-1*), or *Nut-1>Pax2/5/8.a transcript variant 2* (‘tv2’, transcript model *KY21.Chr6.690.v2.SL1-1*). The experiment was repeated in duplicate. RNA was isolated from 10 hpf embryos using a Monarch total RNA miniprep kit (New England BioLabs Inc.) RNA was stored at −80°C until sample analysis and sequencing by the Molecular Evolution Core at Georgia Tech as previously reported (Johnson et al., 2024). Briefly, total RNA integrity levels were using by the Agilent Bioanalyzer RNA 6000 Nano kit and all samples had RINs above 9. Enrichment for mRNAs was performed using the NEBNext Poly(A) mRNA isolation module and Illumina libraries were prepared by the NEBNext Ultra II RNA directional library preparation kit. Libraries were pooled and sequenced on the NovaSeq 6000 with an SP Flow Cell, to obtain PE100 bp reads. Pax2/5/8.a transcriptional variant fold change Pearson correlation analysis and graphs generated in Prism (9.5.1).

Data quality control and analysis were performed in Galaxy (usegalaxy.org) (Galaxy Community, 2022). Raw reads were quality controlled with FastQC and Cutadapt. Reads were mapped to the HT_KY21 *C. robusta* genome with RNA STAR and checked using the Integrative Genomics Viewer (IGV; Version 2.14.1) (Satou et al., 2005, 2022). The number of reads per annotated genes were counted using featureCounts and DESeq2 was then used on the read counts to normalize them to the controls. Datasets were then annotated with the HT_KY21 genome (Satou et al., 2022). KY21 gene models were linked to KH gene models using the Ciona Gene Model Converter https://github.com/katarzynampiekarz/ciona_gene_model_converter. For each step, quality reports were aggregated using MultiQC. Raw FASTQ files can be found in the SRA database under BioProject accession number PRJNA981160. Analyzed data are provided in Table S1.

### scRNAseq re-analysis

Re-processed scRNAseq data from Cao et al. were analyzed (Cao et al., 2019; Johnson et al., 2024). Data from *Ciona* embryos in the Mid-tailbud II stage (roughly 10 hpf) were analyzed using the Seurat v3 package in R to identify cell type clusters based on unique gene expression markers (Fig. S6A) (Satija et al., 2015; Stuart et al., 2019). Replicates were integrated and pre-processing and clustering were performed using the SCtransform and FindMarker functions (Hafemeister and Satija, 2019). Cluster 25 was identified as containing neck cells based on high enrichment for *Pax2/5/8.a* reads (Fig. S6B, Table S2). Cluster 25 cells were re-clustered based on differential gene expression, resulting in two sub-clusters (see ‘Results’ section). The FeaturePlot function was used to visualize feature expression in low-dimensional space, and to use colors to map out the relative expression levels for each gene of interest in the neck and brain clusters. Additionally, VlnPlot and RidgePlot functions were applied to the expression distributions within the clusters, allowing for the heterogeneity of the neck cluster to be further examined and the potential for additional sub-populations noted. R objects and code can be accessed on OSF at https://osf.io/uc32x/.

### RT-PCR of *Phox2* cDNA

RNA was isolated from 17 hpf *C. robusta* larvae using the Monarch Total RNA miniprep kit (New England BioLabs). cDNA was produced using the Omniscript reverse transcription kit (QIAGEN). The resulting cDNA was diluted in nuclease-free water and stored at −20°C. Based on the RNA model discovered during RNA sequence analysis, forward (5′-CATAACGATGGACTACCCTGC) and reverse (5′-CAGACATGTCGTGGTAGGATAGG) primers targeting the new exon 1 of *Phox2* and stop site of *KY21.CH14.159*, respectively, were used to perform PCR on the single-strand cDNA with OneTaq 2X Master Mix (NEB, M0482S) in 50 μl reactions with a touchdown PCR program. PCR products were purified (QIAquick PCR purification kit; QIAGEN) and TOPO-cloned into a dual promoter empty vector (Thermo Fisher Scientific, 450640). White colonies grown on LB plates with 100 mg/ml ampicillin and coated with X-Gal were selected and tested for an insert of correct size by restriction enzyme digest. Plasmids of the appropriate size were sequenced by Sanger sequencing with M13 forward and reverse primers (Eurofins Genomics).

## Supplementary Material



10.1242/develop.202719_sup1 Supplementary information

Table S1. Differential gene expression in 8. overexpression conditions as Pax2/5/measured by bulk RNAseq.

Table S2. Transcripts enriched or depleted in Cluster 25 of mid-tailbud II scRNAseq re-analysis.

Table S3. Transcripts enriched or depleted in subcluster 0 (Neck) vs. subcluster 1 (larval brain) within Cluster 25 of mid-tailbud II scRNAseq re-analysis.

Table S4. Cross-referencing subcluster 0 scRNAseq differential gene expression and Log2 fold-change from Pax2/5/8.a overexpression bulk RNAseq.
