# Supplementary material for: Specification and survival of post-metamorphic branchiomeric neurons in a non-vertebrate chordate
Source: Development. 2024 Jul 17;151(20):dev202719. doi: 10.1242/dev.202719 (PMC11273300; doi:10.1242/dev.202719)
Supplement: Supplementary information [file develop-151-202719-s1.pdf]

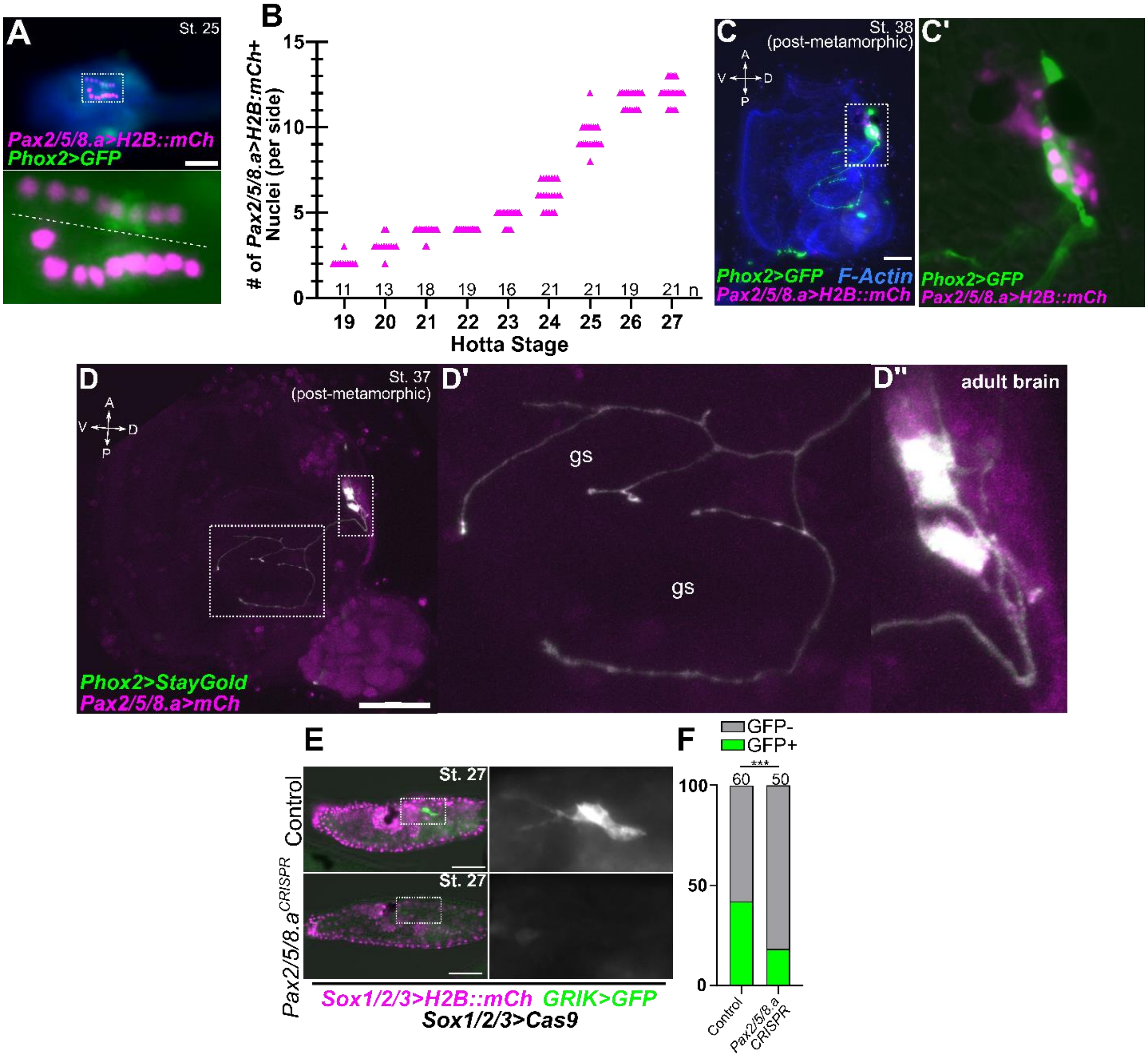

Fig. S1. Neck cell labeling in larval and juvenile brain.

A) A Stage 25 (~14 hpf) embryo expressing *Pax2/5/8.a>H2B::mCherry* (magenta) and *Phox2(C.intestinalis)>Unc76::GFP* (green). The Neck on the left of the midline (dashed line) has nine Pax2/5/8.a+ nuclei compared to only eight on the right side, demonstrating that Neck cell divisions can vary between left/right sides within an individual. B) Plot showing the number of *Pax2/5/8.a>H2B::mCherry*+ nuclei counted in embryos from each stage. C) A Post-metamorphic Stage 38 (~96 hpf) juvenile shows *Phox2(C.robusta)>Unc76:GFP*+ neurons innervating the gill slits. C') Close up juvenile brain shows *Pax2/5/8.a>H2B:mCherry*+ nuclei (magenta)

Development • Supplementary information

Phox2(*C.robusta*)>Unc76:GFP+ neurons (green). D) Confocal Z-stack projection of a stage 37 (~72 hpf) juvenile showing *Pax2/5/8.a>Unc-76:mCherry* (magenta), *Phox2(C.robusta)>Unc-76::StayGold* (green) CMNs innervating the first two gill slits (gs). Insets showing higher magnification view of D') gill slits and D'') brain. Merged white (green + magenta) signal due to all GFP signal colocalizing with that of mCherry. A: anterior, P: posterior, D: dorsal, V: ventral. D) Proposed cell lineage and cell division timing for the early neck. Hypothesized cell divisions displayed with dashed lines. E) Stage 27 larvae (~20hpf) expressing control or *Pax2/5/8.a*-targeted guide RNAs along with *Sox1/2/3>H2B::mCh*, *Sox1/2/3>Cas9*, and *GRIK>Unc76::GFP*. F) Quantification of animals with GRIK>Unc76::GFP+ neck neurons. Scale bars are 50 μm. In Panels B and F, n = number of embryos examined per stage. Statistical significance denoted by *** $p < 0.001$.

Development • Supplementary information

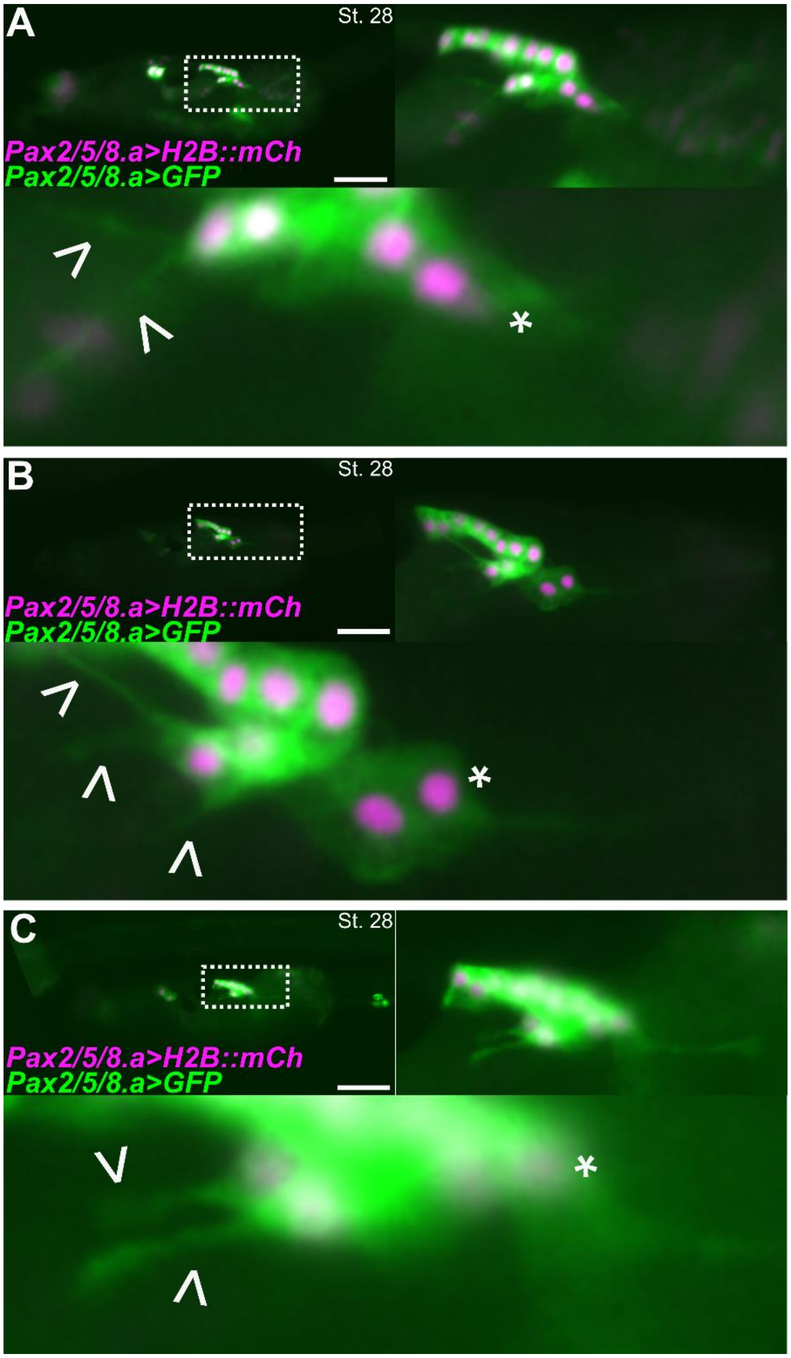

Fig. S2. Labeling of neck neuron neurites. Additional images of Stage 28 (~18 hpf) embryos electroporated with Pax2/5/8.a>Unc76::GFP (GFP) and Pax2/5/8>H2B:mCh neck-specific fluorescent reporters. In these three examples, GFP+ neurites exiting the anterior neck neurons

Development • Supplementary information

are sufficiently spaced to show individual neuron-like processes spatially separated from the posterior neck neuron. A) The most anterior neck neuron has two distinct neurites. B) The first and second anterior neck neurons have distinct neurite processes. C) Another example of the anterior first and second neck neurons with distinct neurite processes. In enlarged images, > are marking neurites exiting anterior neck neurons and * indicates the posterior neck neuron with descending axonal projection. All scale bars are 50 m.

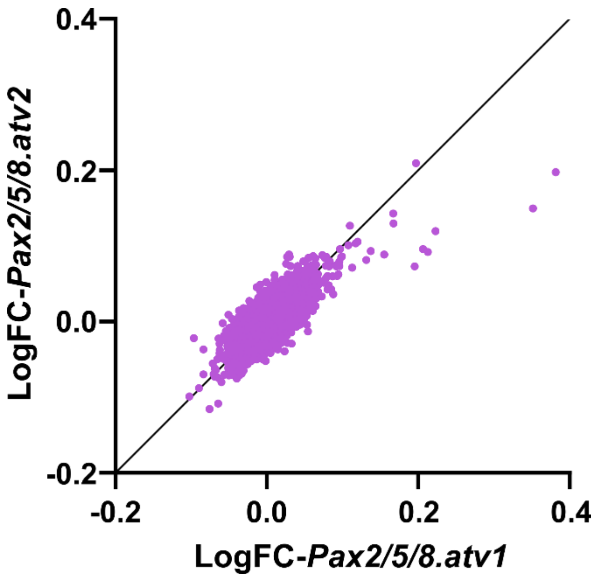

Fig. S3. Correlation of Pax2/5/8 transcriptional variant RNA sequencing data. Dot plot matrix showing comparison between average Log2 fold-change values for all 16,252 genes upon overexpression of Pax2/5/8.a transcript variant 1 (tv1) and 2 (tv2). Raw data can be found in **Table S1**.

Development • Supplementary information

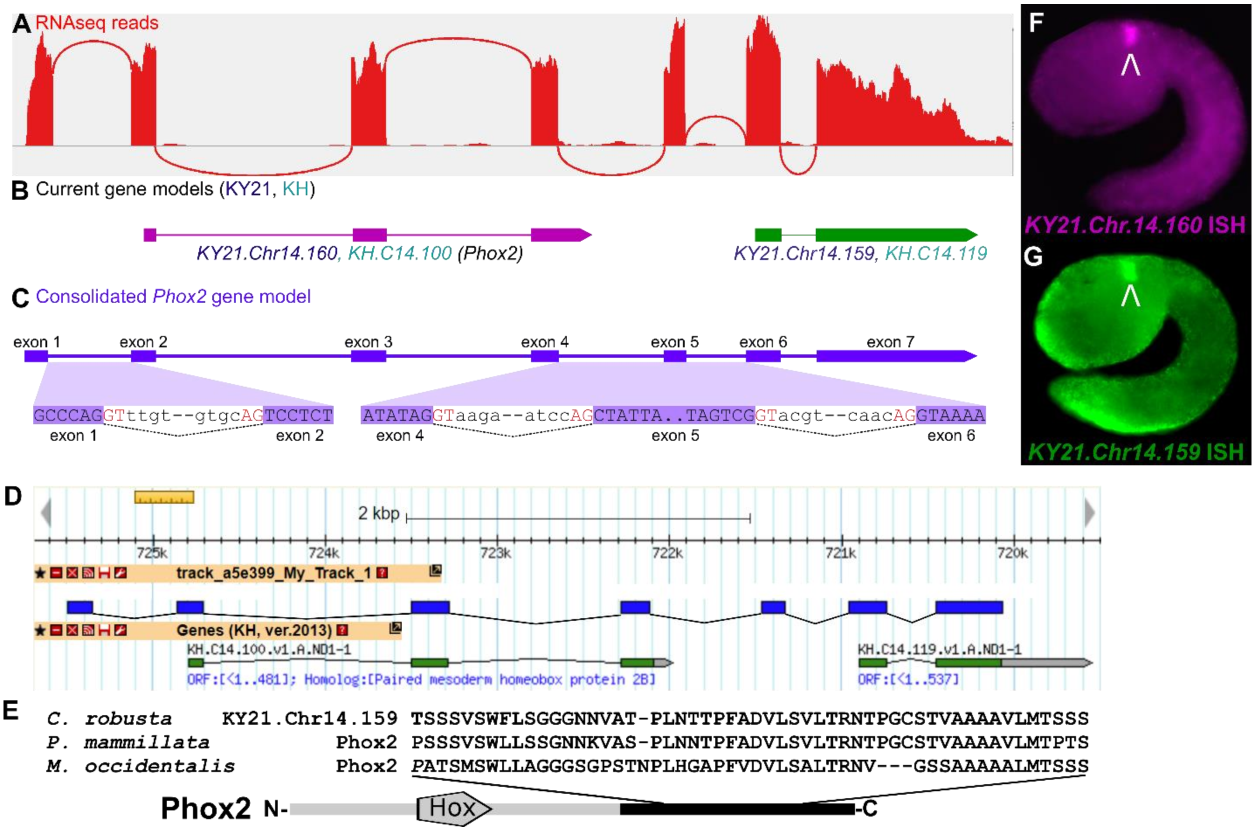

Fig. S4. RNAseq reveals cryptic exons in an updated *Phox2* gene model.

(A)“Sashimi” plot of RNASeq reads mapped to the *Phox2* locus in the *C. robusta* genome in (adapted from IGV). Red lines indicate reads spanning exon-exon junctions. B) Current Kyoto 2021 (KY21) and KyotoHoya (KH) gene models. Comparison to RNAseq reads in (A) shows that KY21.Chr14.160/KH.C14.100 and KY21.Chr14.159/KH.C14.119 should be consolidated into a single gene models, and reveals the existence of cryptic exons 1 and 5, which are not represented in any current *Ciona* gene model. C) Updated, correct *Phox2* gene model confirmed by cDNA cloning, showing splice donor (GT) and splice acceptor (AG) sequences flanking newly identified exonic and intronic sequences. D) Alignment of *Phox2* cDNA sequence (blue) to the KyotoHoya (KH) *Ciona robusta* (*intestinalis* type A) genome (green). The sequence reveals a cryptic exon 1 and 5 of Phox2, linking gene models KH.C14.100 and KH.C14.119. Panel adapted from KH genome browser(Satou, Kawashima et al. 2005). E) Alignment of proposed Phox2 C-terminus amino acid sequence and Phox2 protein sequences from *Phallusia mammillata*

Development • Supplementary information

(*Phmamm.g00000831*) and *Molgula occidentalis* (*Moocci.g00011046*)(Brozovic, Dantec et al. 2018). F-G) *In situ* mRNA hybridization using probes designed for *Phox2* (F) or *Chr14.159* (G) current gene model shows identical expression pattern in the Neck at stage 22 (~10 hpf), further suggesting the two gene models are likely to represent a single *Phox2* transcript.

Development • Supplementary information

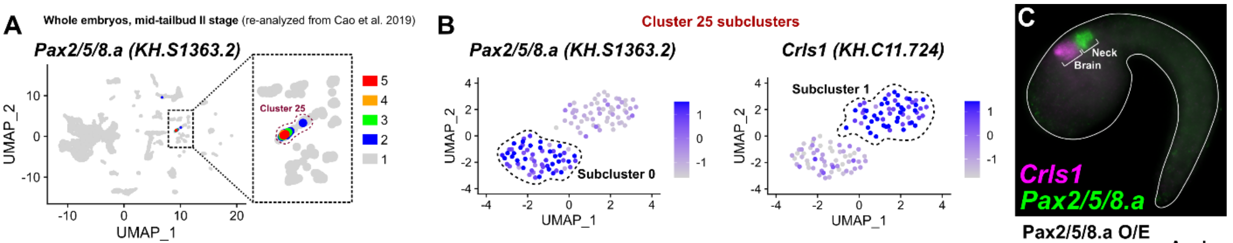

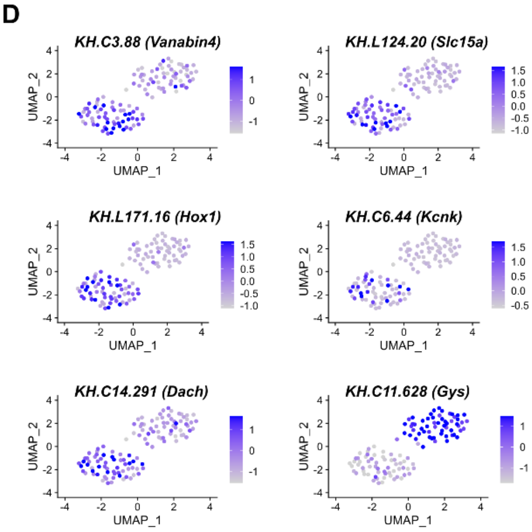

E

Gene	KH.	KY21.	scRNA logFC	Pax2/5/8.a O/E rank # tv1	Pax2/5/8.a O/E rank # tv2	Avg Log FC Nut> Pax2/5/8.a
*Pax2/5/8.a**	S1363.2	Chr6.690	2.09	6	1	0.20
*Phox2**	C14.119	Chr14.159	1.71	4	14	0.15
*Hox1**	L171.16	Chr1.841	1.46	13,181	14,572	-0.01
Slc15a	L124.20	Chr5.1075	1.33	19	23	0.09
Vanabin4	C3.88	Chr3.256	1.16	1	2	0.29
Dach	C14.291	Chr14.603	1.09	10	15	0.12
Abcb6	C11.189	Chr11.212	1.04	14	9	0.11
Gephyrin	C11.23	Chr11.1065	0.96	880	623	0.03
Kcnk	C6.44	Chr6.248	0.95	40	17	0.08
Scube2	C10.286	Chr10.480	0.95	13	8	0.11
*FGF9/16/20**	C2.125	Chr2.824	0.74	442	567	0.03
*Eph.c**	C7.568	Chr7.844	0.64	587	227	0.04
Slc6a14	C1.1124	Chr1.723	0.64	90	24	0.07
Piwi-like	C11.102	Chr11.321	-1.30	16,325	16,308	-0.06
Maml	C2.162	Chr2.458	-1.36	15,333	15,535	-0.02
BCO	C9.224	Chr9.932	-1.41	16,336	16,353	-0.10
GBE.a	C6.224	Chr6.250	-1.93	16,317	16,281	-0.05
Crls1	C11.724	Chr11.285	-1.99	1,368	1,876	0.02
Gys	C11.628	Chr11.757	-2.28	14,872	12,448	-0.01

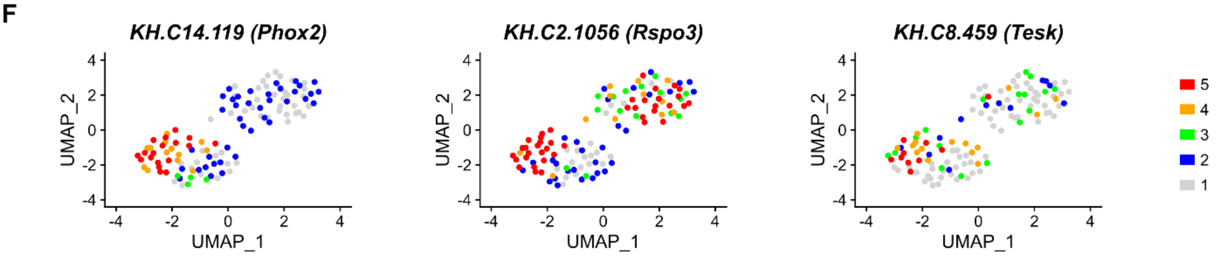

Fig. S5. Analyzing neck-specific gene expression in single-cell RNAseq data.

A) Re-analysis of published whole-embryo *C. robusta* single-cell RNA sequencing (scRNAseq) data from (Cao, Lemaire et al. 2019) revealed a cluster of cells (Cluster 25) enriched for neck marker *Pax2/5/8.a* (magnified view of boxed area in inset). Differential gene expression "FeaturePlot" color-coded as measured by "RNA" assay in Seurat. B) Differential gene expression as measured by "integrated" assay of reclustered cells from Cluster 25 showing enrichment of *Pax2/5/8.a* reads in Subcluster "0" relative to subcluster "1", and enrichment of *Crls1* in subcluster "1" relative to subcluster "0". C) Two-color double mRNA *in situ* hybridization for *Crls1* (magenta) and *Pax2/5/8.a* (green) in a Stage-22 embryo confirm that subcluster "0" represents the neck and

Development • Supplementary information

subcluster “1” represents brain/posterior sensory vesicle cells just anterior to the neck. D) Differential gene expression FeaturePlots showing enrichment of various genes in the neck (subcluster 0) relative to the brain (subcluster 1), or vice-versa (e.g. *Glycogen synthase, or Gys).* All plots generated by “integrated” assay in Seurat. E) Table of genes comparing enrichment (blue font) or depletion (red font) in subcluster “0” cells to upregulation upon overexpression of *Pax2/5/8.a* transcript variants 1 and 2. “Avg. Pax2/5/8/a logFC” = average tv1 and tv2 logFC values as compared to a negative control. Asterisks denote previously known neck marker genes. *Hox1* is a rare exception of a gene that is enriched in the neck by scRNAseq but is not upregulated by Pax2/5/8.a. For *Phox2,* the bulk RNAseq values correspond to the averages between the two incorrectly split gene models corresponding to the full length *Phox2* (KY21.Chr14.160 and KY21.Chr4.159). F) Differential gene expression FeaturePlots (5-color scale, “integrated” assay) showing higher expression of *Phox2, Rspo3,* and *Tesk* in roughly half of subcluster 0, recapitulating the distinction between *Phox2+* middle cells and flanking Phox2-negative cells seen by reporter assays (**Fig. 2**).

Development • Supplementary information

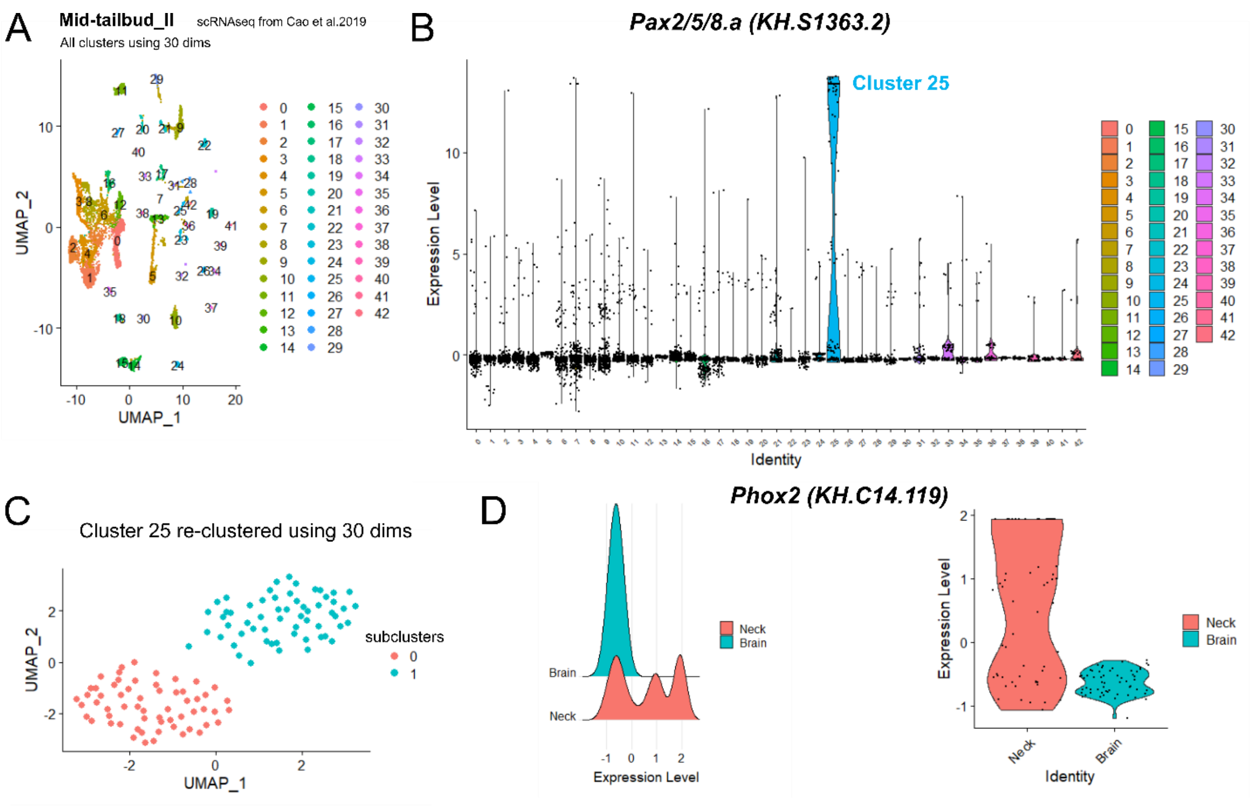

Fig. 6. Reclustering of single-cell RNA sequencing data.

A) Clustering of re-analyzed scRNAseq data from Mid-tailbud II stage(Cao, Lemaire et al. 2019). B) Cluster 25 shows enriched expression *Pax2/5/8.a*. C) Re-clustering of cells only in Cluster 25 revealed subcluster “0” (Neck) and subcluster “1” (brain) showing clear separation. D) Phox2 expression is further enriched in a specific subset of subcluster 0 cells, as indicated by both ridge (left) and violin (right) plots, mirroring the detection of *Phox2>GFP* reporter expression in only a subset of Neck cells during embryonic stages.

Development • Supplementary information

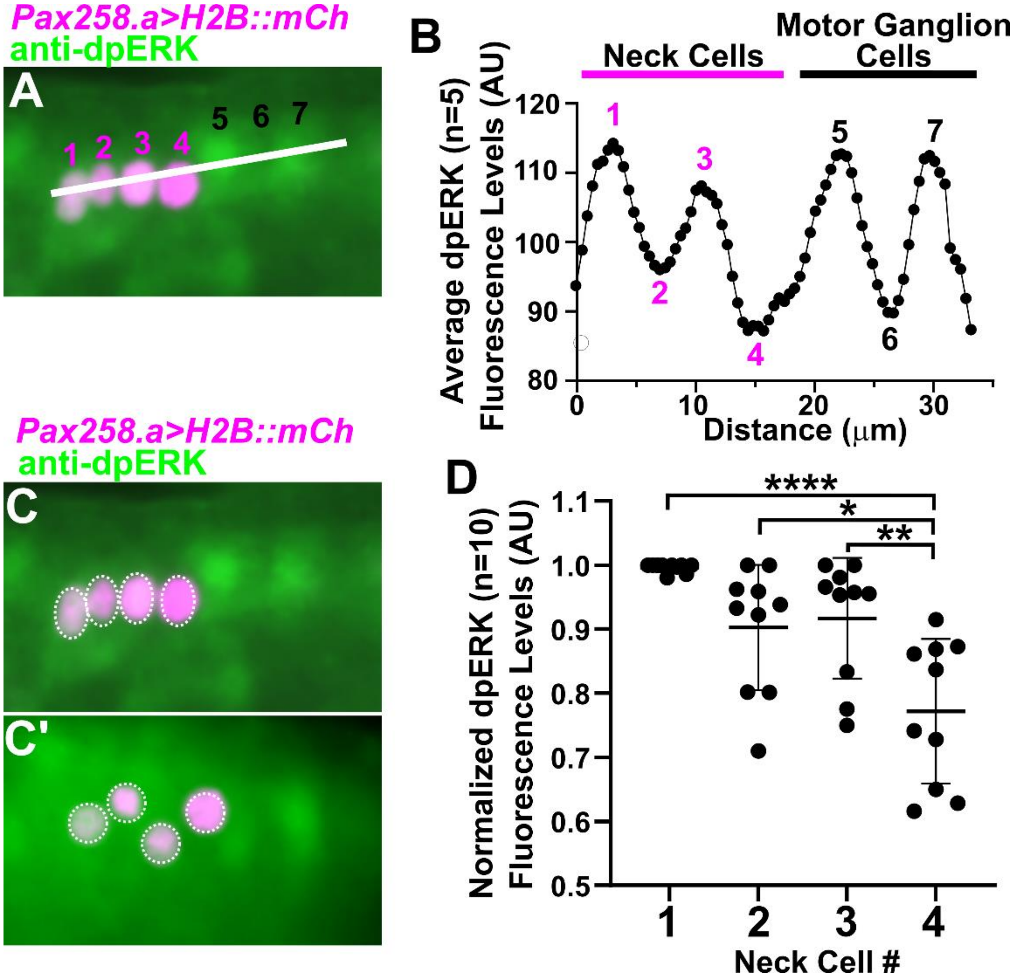

Fig. S7. Analysis of dpERK staining fluorescence levels.

A) To measure the differences in dpERK fluorescence levels across the cells of the neck and motor ganglion we acquired a linear profile of the fluorescence levels from five animals in Image J. B) A line graph of the average florescence level profiles with the relative location of each cell labeled. The boundaries between the columnar neck cells are difficult define and because dpERK typically localizes to the nucleus each Pax258.a>mCh positive nucleus was used as a region of interest (ROI) to measure average dpERK fluorescence levels. C) Example of ROI readings were taken from ten different 9 hpf embryos. In C', animals excluded from fluorescence profile analysis were included in this ROI analysis. D) The levels were then normalized to the highest ROI. Data were paired and non-parametric, so were analyzed by Friedman test and Dunn's multiple comparisons test correcting for multiple comparisons. Fluorescence levels of dpERK staining in the most posterior 4th neck cell was significantly lower than other anterior cells. Statistical significance denoted by * $p < 0.05$, ** $p < 0.01$, and **** $p < 0.0001$.

Development • Supplementary information

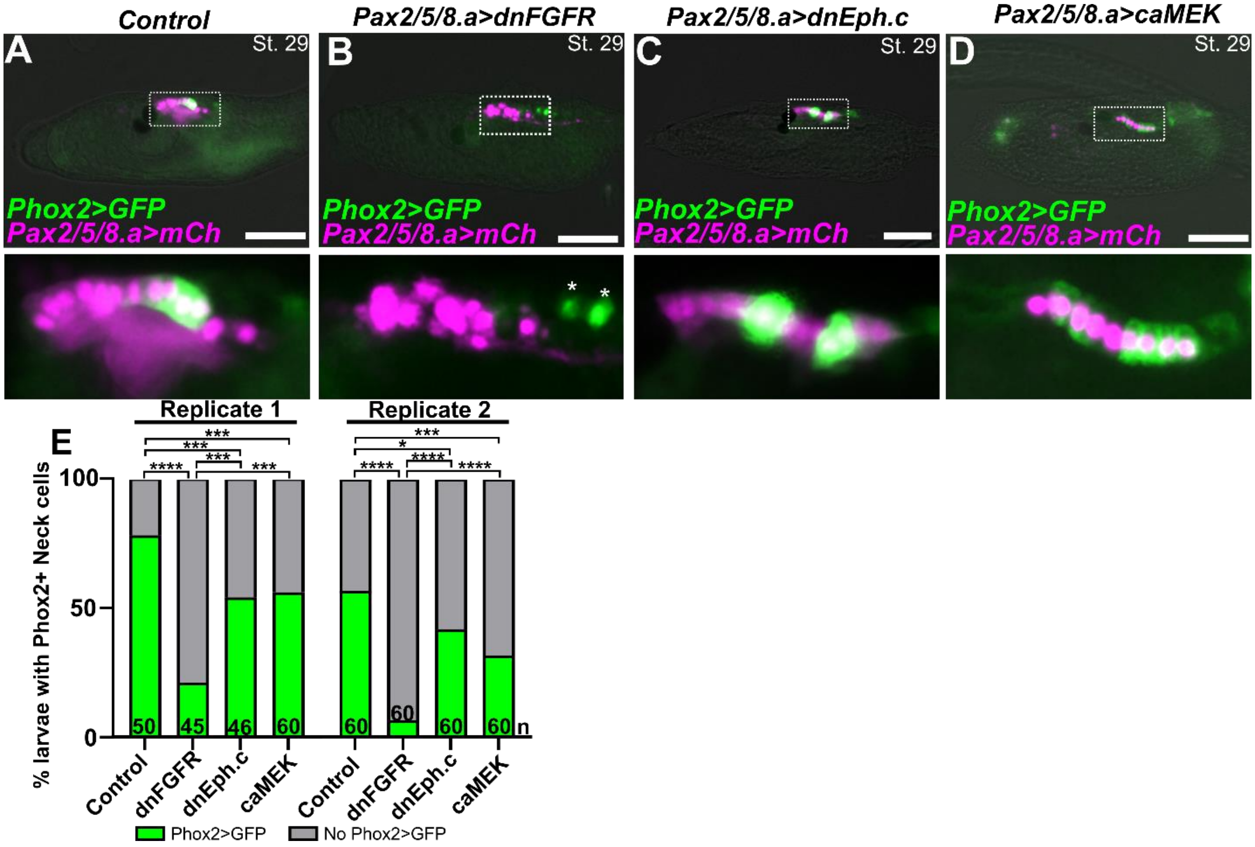

Fig. S8. Manipulations of MAPK impact neck cell expression of Phox2.

A) Negative control larva electroporated with *Pax2/5/8.a>LacZ* control, with *Pax2/5/8.a>Unc76:mCh* and *Pax2/5/8.a>H2b:mCh* (labeled as *Pax2/5/8.a>mCh),* showing *Phox2(C.robusta)>Unc-76::GFP* expression in a subset of neck cells (green). B) Overexpression of dnFGFR in the neck abolished *Phox2>GFP* reporter expression. C) Overexpression of dnEph.c expands *Phox2* expression in a variable manner. D) caMEK overexpression is like that of dnEph.c, resulting in variable expansion of *Phox2* reporter expression. E) *Phox2>Unc-76::GFP* expression was scored in larvae represented in panels A-B, across two independent replicates. While dnFGFR results in loss of *Phox2* reporter expression, dnEph.c and caMEK do not. In panel E, n = number of individuals scored in each sample. All scale bars = 50 µm. Statistical significance denoted by * p < 0.05, ** p <0.01, *** p <0.001, and **** a p < 0.0001.

Development • Supplementary information

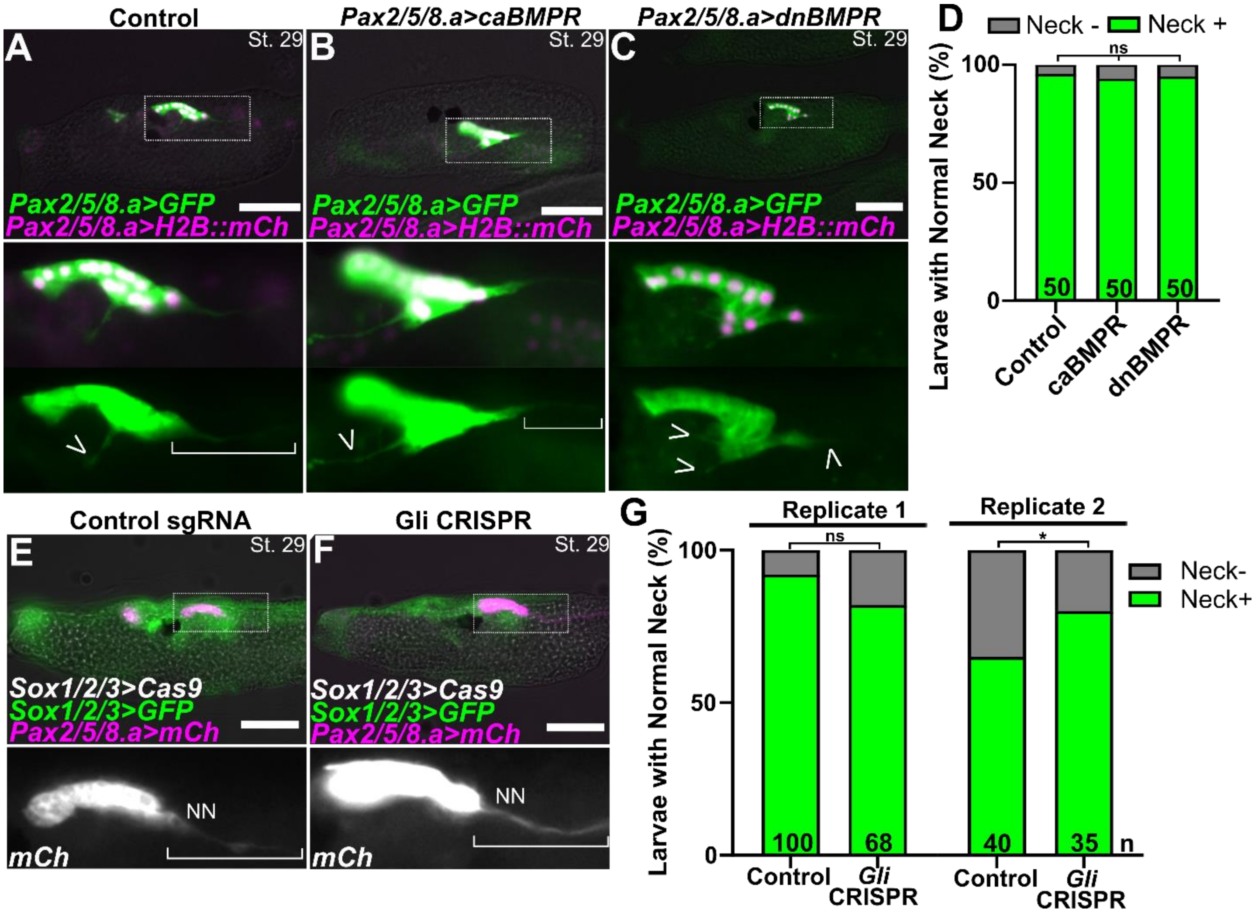

Fig. S9. BMP and Hedgehog signaling do not affect differentiation and 10 morphogenesis in the neck.

A) Negative control larva electroporated with *Pax2/5/8.a>lacZ, Pax2/5/8.a>Unc76::GFP* (*Pax2/5/8.a>GFP;* green), *and Pax2/5/8.a>H2B:mCh* (magenta) showing typical morphology of the Neck, with some neuroepithelial cells anteriorly and differentiating neurons and axons (bracket, open arrowhead) in the posterior. B) Larva expressing a constitutively-active BMP receptor (caBMPR), showing no noticeable difference in Neck-derived axons (bracket and open arrowhead). C) Larva expressing a dominant-negative BMP receptor (dnBMPR), also showing no noticeable effect on Neck morphogenesis or patterning. D) Scoring for “typical” Neck morphology in larvae represented in panels A-C. E) Negative CRISPR control larva expressing *Sox1/2/3>Cas9:Geminin*$^{N\text{-}ter}$ and *Sox1/2/3>Unc76::GFP* (*Sox1/2/3>GFP*, green) reporter, with *Pax2/5/8.a>Unc-76::mCherry* (*Pax2/5/8.a>mCh*; magenta) labeling the Neck including the axon

of the Neck neuron. F) Knocking out the Hedgehog effector-encoding gene *Gli* also does not alter Neck morphogenesis. G) Scoring of larvae represented in E and F. In panels D and G, n = number of individuals scored in each sample. All scale bars = 50 µm.

Development • Supplementary information

Supplemental Figure 10

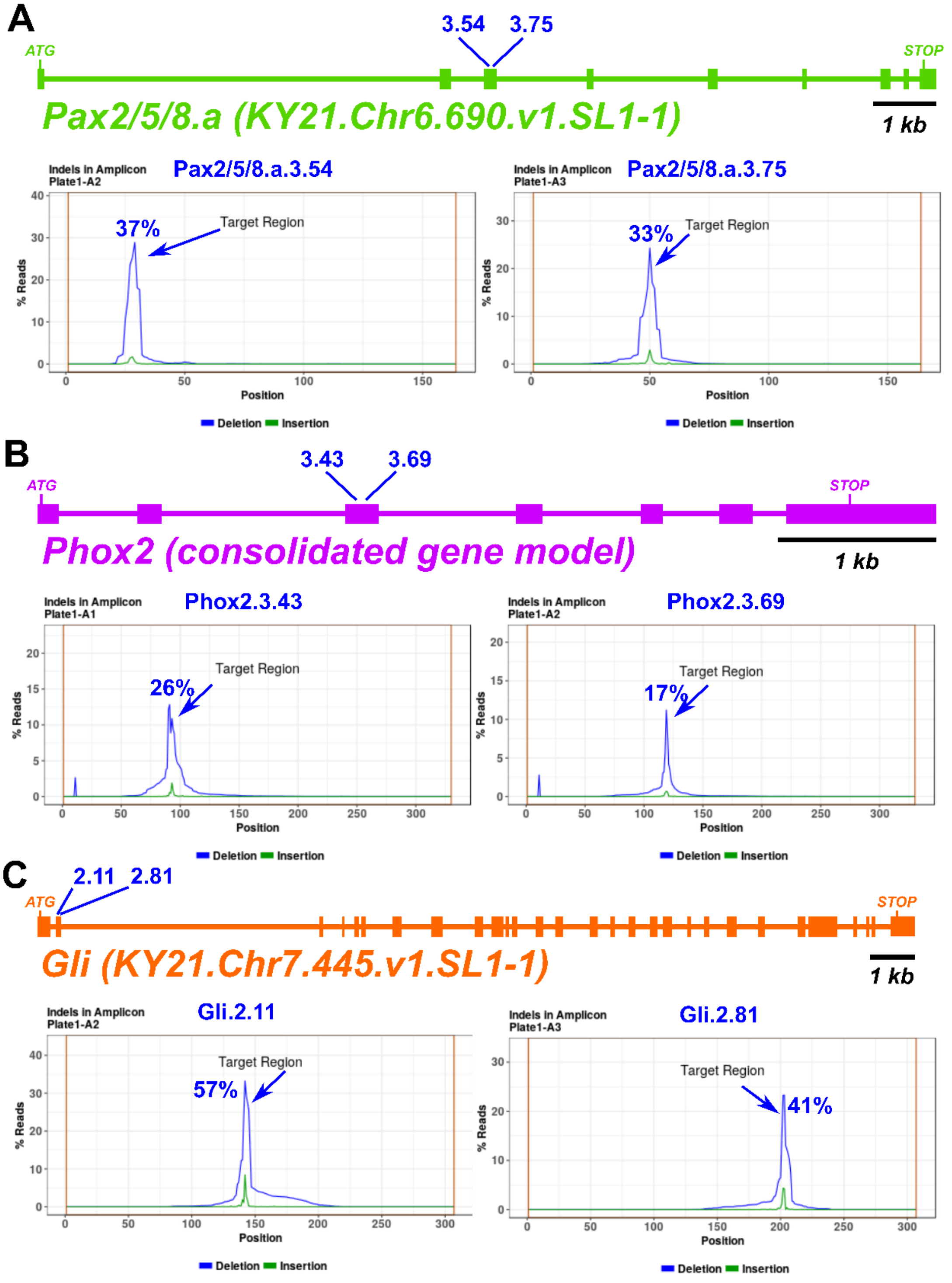

Fig. S10. Validation of sgRNA efficacy data and gene models.

Indel plots generated by Amplicon-EZ Next Generation Sequencing from Azenta/Genewiz for validation of sgRNAs targeting (A) *Pax2/5/8.a*, (B) *Phox2* and, (C) *Gli*.

Development • Supplementary information

Table S1. Differential gene expression in 8. overexpression conditions as Pax2/5/ measured by bulk RNAseq.

Available for download at
https://journals.biologists.com/dev/article-lookup/doi/10.1242/dev.202719#supplementary-data

Table S2. Transcripts enriched or depleted in Cluster 25 of mid-tailbud II scRNAseq re-analysis.

Available for download at
https://journals.biologists.com/dev/article-lookup/doi/10.1242/dev.202719#supplementary-data

Table S3. Transcripts enriched or depleted in subcluster 0 (Neck) vs. subcluster 1 (larval brain) within Cluster 25 of mid-tailbud II scRNAseq re-analysis.

Available for download at
https://journals.biologists.com/dev/article-lookup/doi/10.1242/dev.202719#supplementary-data

Table S4. Cross-referencing subcluster 0 scRNAseq differential gene expression and Log2 fold-change from Pax2/5/8.a overexpression bulk RNAseq.

Available for download at
https://journals.biologists.com/dev/article-lookup/doi/10.1242/dev.202719#supplementary-data

Table S5. PCR primers for amplicon sequencing by NGS

Gene + exon	Forward primer	Reverse primer
Pax2/5/8.a exon 3	TTACGAGACAGGCAGCAT	CACAAAACACAATTTCGGGTAATT
Phox2 exon 3	ATTGCACCTGGGCTAGAAGA	TATTGGTAATATTGTATGTCGCAAC
Gli exon 2	GTGGAACGTCCATGTGGTC	AACATGCGAATATGTCCTATCA

Development • Supplementary information

Table S6. Gene Models

Gene	KH ID	KY21 ID	ANISEED ID
Pax2/5/8.a	S1363.2	Chr6.690	Cirobu.g00013823
Phox2 (Nter)	C14.100	Chr14.160	Cirobu.g00003507
Crls1	C11.724	Chr11.285	Cirobu.g00002581
Phox2 (Cter)	C14.119	Chr14.159	Cirobu.g00003527
Gli	C7.334	Chr7.445	Cirobu.g00008171
Vanabin4	C3.88	Chr3.256	Cirobu.g00006092
FGF9/16/20	C2.125	Chr2.824	Cirobu.g00004295
Eph.c	C7.568	Chr7.844	Cirobu.g00008427
EphrinA.b	C3.202	Chr3.875	Cirobu.g00005364
EphrinA.d	C3.716	Chr3.881	Cirobu.g00005918

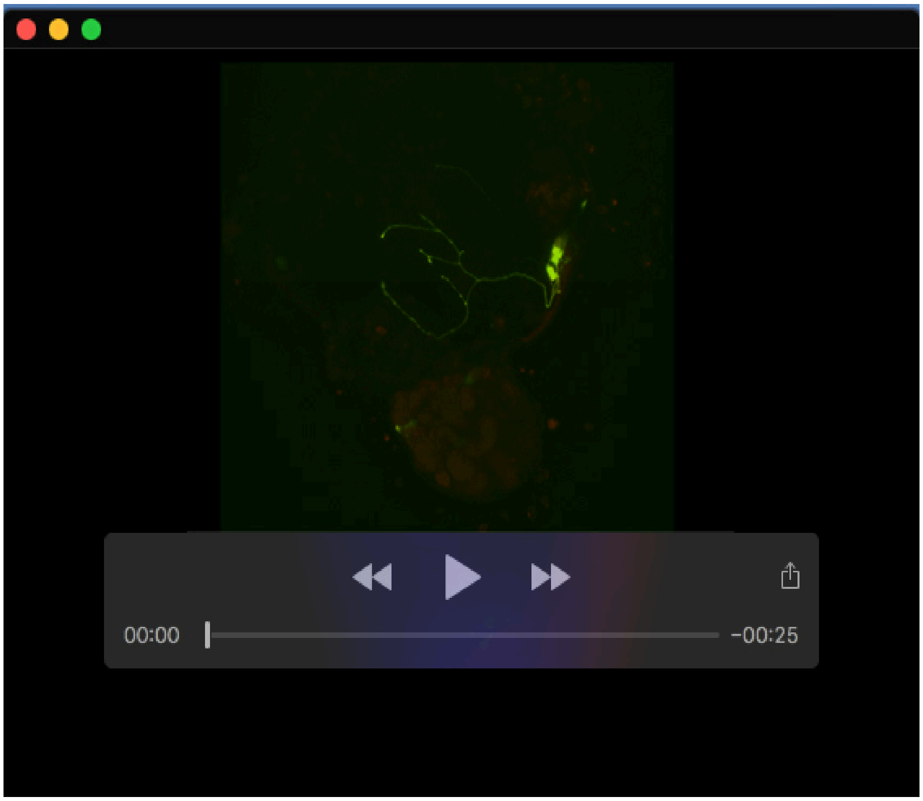

Movie 1. 3D projection of CMNs in post-metamorphic juvenile shown in Figure 2L.

Supplementary Materials and Methods

Some sequences are based on genome assemblies and might not be verified by sequencing after cloning. ANISEED unique gene IDs of marker/reporter genes are at the end of the document.

>Pax2/5/8.a -1961/-1 [smaller version of reporter first published by Oonuma et al. 2021(Oonuma, Yamamoto et al. 2021)]

TGTCAGCTAGTGCACTCTTACAGTGTTGCCAGCTTTTGTTTTTGCTGGGCCTAATGTTGCCAGCTATGTC
CCCAAGCACTTCTCAGTGCTTCTACATGTCTTTGAAACAAATGCAAAGGAATTGTTGCAACAATCTTACT
TGATAAAGATTAGCATCTACTGTAATGTTCATTCCTTCCGAGTGTTTTGTCTGTCGGGACTTGAATCTTA
CAGCGGTTTTGTAGTGTCTCGGTTTGCTTTGCGAGAACGCTGTCCCAGTATACATATACTCGAGTAATAT
CAATATTTAAATTCCTTTAATTCTTTAATAAATTAAATTAGTATAAAGTCCCCAACACACCGCTAAAGTT
AAATTATTTAACACAACATCGCGTTTGGCATAATTGTTACGATGGCCACGTCACAATTACGCTTTACGTC
GATGCGGTGGGTCAGCTACGTCATGACCCTGCGTTCGGTGCGGTTACGACACGGTCGGCTTAGTTACGTT
ACAGTTGCGGGTTTGTTACGTGGACGTCGCCGCTTTGCGGTTCGTAACAGCCACAGTATGTCGTAACGGT
CGGTGCGGTGCGTAACAGCCGGACGATATTATATTGTGCGAAGATACGACGAGAGTATCGCGGTACCAAG
ATGTTTTCTCAATAAGCGGTCGGGTGATGCAGGGCCGTTGTTTGCTTTAGACTTCTTGCAGGTGGGCACT
TGGTGGCGTGCGCACTGCAGGGCTTTTCTGTGAGACTTGCAACTTTTCGTTTGACGGTGAATGCTTGTTG
GTGTGACTGTTTTTGTCTATGGGTTTCTTTTCCTTGCTTGGGTAGGGTCTATCCAGCAAAGGGTTTCTCA
CCAGAGTAGAAACCCACTACCATTGGCTGGGGGCTTGTCGTGCCGTAGTGGCTTTCCACCAACGCCAGCC
ACATAAACTATGAAGGTATTTTAATCCTGTTTTTATTTTCCAGAATTTCAGTTTCAGATATTTTCATCCG
CACGTGGGCGCTAGCGATTGTTGGAATTACAGTTCGCATTGTTTGTAATTGTTATAATTGTAAGTCGCTT
TGTGCTGTGACGTCATAATAAAATATATTTCTGAACTTTGATTACGTCATATTCGTGTTGCTTAATGATG
AACTCGCGATACTAGTGACGTAATAAATGAAATTTGATTTTAATTTGATTGTTCTGTAGACGCCGCCACG
CCGAAGTCCAACTGCGTCGAACTGACAATACGCTGTGTGACGTCATAATGAAACAAGTGCATGAGGTAAG
TGACGTCACAATCGTGGATTAAGAGCGGAAGTTATCGCGTTACGTATTATGACGTGTACAATGTTTATTG

Development • Supplementary information

TTTCATAGTTAATCGGACGGAACAAATTAACTTGTGACGTCAGAGGGGTCAAGTCGCTTTATTTGTTTAA

ATTTTAGAATTGTTTAGCTTTTGAGACTAAACTATTGTGACGTCATAAACGAATTTCGCGTCTTCGTTTG

TCAACGTTTTTGTTACTTGTGACGAAATAATAACTAATTAGAAATGCTGACGTCCTAATGTATTACGTAA

TAATATTGTGATGACGTAGCATGATAATGACGTAGCTCGGGGTAAAATCTACGCCTAAATTTTAAGAGAG

AGCTTAAAAAAAACGAGAGCGGCGATAGAAAAAGGCGACTCCCTTTTTTCCACCCTGTCTCTTATATGTT

GGAGTGAAATCTTCGCATCTTCCATAAAGTTAAAAGTTTCGAAGATTTCACTGTTGGGTGATCGTTGCAG

AATAATCACTATAACTAGGGTTTGTTACGTCATAAAAGATTTAGGGATAATTATTTATTTTGTAGAATTC

TTATGCATTGTTACGTAGTAAGCGTTACGACGTCATACAGCAACCGCATTTTGTCTATTTATCGCAAAAT

TAGCTTTCTGACGTCACTGCATTATGACATCATCATGTATTACGTCACACAGGTGTTAGTAAGTTTGgAT

>C. intestinalis (Type B) Phox2 Promoter -2951 to -1 [Dufour et al. 2006(Dufour, Chettouh et al. 2006)]

CCCATCGGCGTTTTAAAGTCGGCTATTCTCTCTCTCAAATTGGAATTAACTCTAAATTGAAGTTTATGCG

GTATCCATATGCGAAATATAGGGCTCGTTTATTGGCAGCATCTGGCCGAGTGTTTCGACCACCGGCAATA

ATAACAGCACACCTACGCTCCACTGTTAGAACGTGAACCGGTCTTTGATGGCCAGAATAACAGCTCGGCT

TCGCTAAACGACATTTGGTGAACGATGATACTTCTTAGGTTTAACCGAACAGCGGGGGGCAGCTATACCC

GAACGCGACTCGTATTCATTTTCAGCGAGTTATTGTATCATATAGTAGGGTGGGGGAAGATGGGACACCT

TTAGCACATAATGTTTAAATATCCTGATCGTGTTTTAAACAAAACAATGGTCTATGGGAGTCGTGAGAAT

GCCGTTATATAATTCGTTGAATATTGTTTGTTTACTACCATATGCAACGAGAAAATGGAATGAAAAGGTG

TTCCATCGTCCCCCACCCTACTACACTGAATGCTTTACCGACAATGAATATTGGTTTGGTCATAGCACGC

TATAACTTCACGCTATGTAAAGAAGTGTGGATGCGAAAATTCTCATGATGTAAATTATAACAGACAACTA

ACTAATAGAAATGAACCCGTTTCAACTTTACCGCTGAGATATTCAATGAGGCGTTATACCGATTCCGGAA

TTAGTTGATAATTGCTTGAAGTTCCGAATGCGACACCCAAACAAAGGCACCCCGTATACCCATTTAGTGT

TACAAGCCTTGTCAAACAGATTAATGAATTTAATTCGCATGAGAAAATAATGTTTATTACATTTGAATAT

TGTTTCCAACATTTGAAAGTTAAAAATTCCTTAAAATCTAATTGGTTTGAAAATCCTTATCAATTTTTAG

AGAAATTTATTTTTCATTTAAACGCTTCCTAGTAAAACAAAACCTAGGCCTAACGTGGTTGCTAAAACAA

AATGGAGACGGTCGGTGCTCGTACAGCAAGAGAGCGAGCCAATAACGCACTCACGAGAAGCAATTAGGCG

CGGAATCAACTTAATCCAAGGTTTCTTCGATAAAAAGCCAACACTGTGTTGTACGTGAGTGCTAACATCG

AGTCAATTCTAACCTGGTTATGTTTGGTGCGCTGACGTCCTCGAGTCGGTAGAAATGTCATTAGAATTCG

ATTCGAAATGGAGTTTATTAAGTAAAGGCGGCGGCACTCGTCGGCCAGCACCTGGCGCTTTTAGTACTGG

AAGGACTGAACAATATACTGAGAAATAGCTTCCACACTTCCACATTTTACAAAAAAAAAATAGTTATGGA

ACACATGTGTTATGTTTTAAATAACATAATAAAACTCTTTGAGTAACGAATTCAGCGGTTTTTCAAAACA

TTTTTATCAAAATTATGTTTTTCTTATTAAACAGTATATTCTTATGGTTTTGGAAGATCTATATACATTA

AAAACACGTAGGATATATTACGGTATTTTTAACATTTTAAATAGTTTTATGTAGGTTTCTTGGTAGAATG

CGATGATTTAAATTCAATCCAATGTATTTCGAGATAAATGCGTTAACAATTGTGTGTTAAAGAAAGAATC

TGCAAACATATTTTGCGGTACCGTCGCTTTTGCCGGCCGGATTTAATCCAATTTGATCATAACGCGTGCC

ATGGTTCCGACCCGAGGCGAATCACTGGTGGGGAAAAAGGGGAAAGGCCAATTGACTTTATATCGGGAAA

GCTAACGTTATTGGGCGGACAGAGCTAAAGCGTCGAGTCATTGCTGTGGCCTAATGCAGTTTAGGTGCGA

AATTGGATTTTGCGATTATGCAGTTATGATCGGAAAGTTACAGACAATCTGTCTCAGCACATATATTAGA

TAACTTAGATTTTCAACAAATTTTCGCTCATGCTGTTTCGGTAGATCGGCTAACGTAATAAACAAATAAT

TTCGTTTTCCGAAAGCAGCATCTCTACTGCATTTGCCTGCCTAACAAGGATTAGATTATAGAACCTGGAA

AGCGACAGTGGCTTAAACGCCGGAATAAAGTGATTAATAAAGCAAAACAAATTAAGTTCAAAACTTAAGG

GCGAATTGCCTGAGGCAGCAGTCATTTTTTTAAAGATAATTTTTACATTTTTGCTATAAAACCTAACCAT

ATAAAAAATTTAAAAAAATCAAATAATAAAACCTTTTATTTTACAGTTGAAAAAATTAAAATATAACGAT

ATGGATTACCCTGCGTATTTAGGGGTTGGCACAAACTATGACACGACTGCTTGCATGGTGGCTGCGGCAG

CTAATAATGATCCACACCAACCATACGTTACTACACCATACGGGGATTTTAACTCATGCGCTCACGCCCA

GGTTTGTGCTGCATTATTTTACCACGCATGGTCTAAAAAGATATTAATTTCTGATAAAGTCGAAATATAT

TTGGGCTTGTAAAATTGTTTTATAGCGCTGTGGGGTAGGATGGGAAACCTTTAGCACCTAAAATCCGTAT

TTCTTGTTCCTGTTTTGAACAATTAACAACGATCTTTTAGAGCCGCAGGGCTACGGTTGTATAATTCTAT

AAATATTCTTTGTTTAGTATGCCAAATGGAACGAGAAAAGAGAATAAAAACACGTCCCATCTTACCCCAC

CCTACTATAACTTTAATTTCTGGTTAAGTCTAAAGAGATTTGAACTTATAACATTCGTTTTATTGTATTT

TTCAATTTCAATTTAACATTTTCATTCATTCGCATCTGCGCCTATTCTTATAATTCAAACAATAATGTCT

GCAAGTGGTGTTGAGCTTACAGTAATTTAAATTCCGAGTTATTTAAATTGAAAGTCAATAACCTTATGTA

CAGTCCTCTCAGAGCAACTTCGCGTCAGCTTATACACCGACAGTCCCGATCCGGAACTCCAGTTTTGGTG
GCGG

>Ciona robusta (Type A) Phox2 promoter -2416 to +15, coordinate based on newly identified start site in exon 1. ATG start codon

CAGAACGACTTGCTCTGACAACTTAATTATAAGCGGCTGGTCAGTCGCATGCGCGGTCGACCGATTACCA
TTTCCGCCCGCGCAAAAATCAACAATTTCTTGAAAATAATAAGAAAAACTTTCGGTGAATTAGCCCTTTT
CTATATACGATATAGACACATTAGTATTTTAAAGACGGTTATTCTTTCTCACAAATTGGAATTAACTCTA
ATTGAAGTTTATGCGGTATCCATATGCGAAATATATGGCCCGTTTATTGGTAGCATCTGGCCGGGTGTTT
CGACCATCGAAAATAATAACAGCACACCTACGCTCCACTGTTAAAACGTAAACCGGTCTTTGATGGCCAG
AATAACAGCTCGGCTTCGCTAAACGACATTTGGTGAACGATGATACTTCTTAGGTTTAACCGAACAGCGG
GGGGCGGCCATACCCGAACACGTCTCGTATTCATTTTCAGCGAAATATTGTATCATATAGTAGGGTGGGG
CGATATGGGACACCTTTAGCACGTAATGTTTAAATATCCTGATCGTGTTTTAAGCAGTTAGCAACATTCT
ATGGAAGTCGTGAGAATATAGATCTAAATTTCTTTAAATGTTCTTTGTTTACTACTAAATGGGATAAGAA
AATAGAATGAAAAGGTGTCCCATCTTCCCCCATCCTACTACACTAAATGCCCAACCGAATACACAATAAA
TATACCGGTTTGGTCATAGTATTTTAACTACACGTTATATAATGATATGTGGGTGAGAAAATTCTCATGG
TGTTAAATTATAACAGACAACCAACTAATGGAAATAAACTCATTTCAACTTAAACGCTGAGATATTCAAT
GAGGCGTTATAACGATTCCGGAATTAGATGATAATTGCTTGAAGTTCCGAATGCGACACCCAAACAAAGG
AACCCCATGGCCGTTTAGTGTAACCAGCCTGGCGAACACATATTATGATTAATGAACTTAATTCGCATGA
GAAAATAACGTTTGTTATATTTAAATTCTAATGCTAGCATTTAAAAGCAAAAATTTCACCAAAATCTGAT
TGGTTTGAAAATATTTTATTAATTTTTTGGGAAAAATATGTTTTATTTCAACGCTTCTAGTGATACAAAA
CCTAGGCTTTAAGTGGTTGCTGAAACAAAATGGAGACGGTCGCTGCTCGTACAGTTAAGCGAGGGAGCCA
ATAACGCACTCACGAGAAGCAATTAGGCGCGGAATCAACTTAATCCAAGGTTTCCTCGATAAAAAGCCAA
CACTGTGTTATACGTGTGTGCTAACATTGAGTCAATTCTAACCTAGTTATGTTCGGTGCGCTGACGTTCC
CGAGTCGGTAGAAATGTCATTAGAATTCGATTCGAAATGGAGTTTATTAAGTAAAGGCGGCGGCACTCGT
CGGCCAGCACCTGGCGCTTTTAGTTCTGGGAGGACTAAACAAAAAACTGAGAAACAGTTTCCACACTTTT
AAATTTTAGAACAAAAAATATATGTTTATGCAACATTTTGTAATGTTTTAAATAAATTAACAAAACTCTT

Development • Supplementary information

TAAGTAACGAAATCGACGGTTTTAAACTATATTAATCAAAACTAACTAAGTATTTCTAAACAAAAATAAT

TCCTTATGCTTTGGAGTATCTTCATACATTCAAAAAAACGTAGGCTATATAACGGTGTTTTTAACATTTT

GAGTAGCTTTATAGATTTTTGGTATAAAAAGCGATGATTTAAATTAAATCCAATACATTTCGAGATATAA

ATGTGTAAACGTTTATGTGTTAAAGAAAGAATCTGCAAACATATTTTGCGGTACCGTTGCTTTTGCCGGC

CGGATTTTAATCCAATTTGATCATAACGCGTGCCAGGGTTCCGACCCGAGGCGAATCATTGGTGGGGAAA

AAGGGGAAAGGCCAATTGACTTTATATCGGGAAAGCTAACGTTATTGGGAAGACAGAGCTAAAGCGTCGA

GTCATTGCTGTGGCCTAATGCAGTTTAGGTGCGAAATTGGATTTTGCGATTATGCAGTTATGATCGGAAA

GTTACAGACAATCTGTCTCAGCACTTATATTAGATAAGTTAGATTCTCAACAAATTTTCGCTCATGCTGT

TTCGATATATCGGCTAACGTAATAAACAAATAATTTCGTTTTCCGAAAGCAGCGTCTCTATTGCATTTGT

ATGGCTAACAAGGATTAGATTATAGAACCTGGAAAGCGACAGTAGTGGCCTAAACGCCGAAATAAAGGGA

TTATTAAAGCAAAACAAATTAAGTTCAAAACTTAAGGGAGAATTGCCCCAGGCAGAAATCATTTTTTAAA

GATAATTTTTAACACTATTTGCTAACCAACCTATCCATGTAAAGAAAATTATAAAAATCAAAATAAAACT

TTTTTTTACAGTTGAAAAAAAAATAAAACATAACGATGGACTACCCTGCGTAT

>Sox1/2/3 -2391 to -1 (Stolfi, Lowe et al. 2014)

CGCTAGCATGTCAAATAGTTCGAATTTTATTGTAGAACTCGGGCAGTTGTGATGTAACACGTGAATTGGA

TTCAGCATTCGTTCTTTAAACCTATGAGTCGGCAAGGAAGCGATCTGTCAAATTAAATCGAATCTTTCAG

CGCTCGCATCGAGACATAGCGATACTGGAAGCGTCGGAAATAGAATAGTGGGGTTGATGACAGGTGAAAA

CAGAATTCGTTGCGCAAATTACGAAATATTTTGCGCTGGTTTTATTACACAACAATTTTCTCAATAGTGG

GGTCGAAATTGATGTTTGCATTTTGTTGTAGGGGTTGTACATGCGGGATACGCTCCGTGGTAGGCGTCTA

TGTTATACAGTCGGTGTAACTTAACGACAAATATAGAATACGTTGTGTTCGTTCATTGATGGTCTTGAGT

TGCACGATTTCCCGTCGCAAATGAACCGTTAACACAACTTGGCCCGAAGCAGGCATGGCAGAAGTCGTCG

TAAGTAGGCTGGGGTGTCGTGGGGCCGCGCGTCGTCATGCTAAAACCGACCGAGCAGAGCGTAGGCAAAA

GTAAGCACTGAATTGAGTGAAACGCGACGAAAAAAACGGTGGTACTTGTCGGTTTGTCCGCGCTGAAACC

TAATAACTGGCTGCGCGTGTTTTCTAGGATGTCGAGTGTTCGCGGACACTTGAACACTTAGATATCTACA

TGCTGTCGTTGCTTTGGTTGGTTTTACACTGGATTTGTATTGCTGGTGTTTTGCTGTAAAGCCAGAGTTT

Development • Supplementary information

GTAACCATATAGACATCGTATATTTGTAATGTAGTATTCCTAGTTTTGAGTATAGAGCTCGCTATTTAGT
TCTGTTGTCAGTTTTAAGTGTGTTAAGGCAAGATACATAAAGAAACAAAGTCTTTATCTATCTTGTCGAA
TAGAGTAACGAAGATGAAATTATTAAAAGTTTAAAAACAAGGAATTAACAGCAAAATGACAGTTGGTAAA
ACACGCTATGAATAAACTGTATTGAAAAACATCAGGAAATCGTTTGGTTTGTGTTCAATTTGTTTAATTT
AGAAGTTTTGTATTGTTAGTATAATGACTGTTGTCTGTACACAACACGTGTTGACCACTATAGTAGTGTA
GTAGCCCAATAACAATTACTGATGTTCTGCGCATAAGTCGCGTGTGCTTTACTGGGCATTCATGCCGGTA
GAATTAACCATTCATCGTAAAAGAACAAAACGGTCTATTCTGATCCGTTCATTGAGTGGCTTATGAATTG
GCAAAAGTGCTTGTAAAGTCGGCACTGAATGGGCGCTGCCTTTGTTACTGACAATGTGCATTCATGACGA
TAGTTCCGAAAGGTGTGGAAGTAAAATACACTGTTTGTGTTCATACTACGCATTCCGAGGAGGAATACCT
TTTTGATACGCCATGTATGCTTGATTTTTTTTGACAAAATTAAACATTACAATTATTGGAACGACCACAA
GGTTGTTAGTTTTACACTTTGTTGATATTTTTGTATGGGTGTTATACGTTAATGGGGATGTTATTGAAAT
ATAGAGTTGTGTAATTGTAATTGTGCGGTTTCATGTTAACGCTTAAAACAGTTTTCTTTCTAAGCTGGTG
TTTTGAGTTTTAGGTATATATGCTAAAGGTTTAAGTATTGGGTTATGAGTTTTGTTATTCGTTTCTTTTT
TGTAAATATAACTTTTGCGGGTTTTCATATTTTTCGATTCGTATAAAATCGAATTCTGTCTTATCATGAA
CCGACCACGTTTTTCGCTGATTCGAAGTCGTTTAGATGGTTGCTTTAGGGAACGCTGGATCCCAACGAAA
AGAAAAAGGCGACGTTCGCCTCCGCGAAGATAAGCCGTAGCGAGAGAAAGGTCGTTTGTAGCGCAATAAA
GGCAGCCTGTGAGATGCCTACTTCATTCATTCTGCCTTTTGTGACTTCATAGTGGCATTGTGAGCTTCTA
TTCAGGCATTGTCTCCAAGAAAGTTATGAGTCCATAGATTATAAGACCTCTTCTATGGACAAGCAGAACA
ATTGAATTACAATTGTTAAAAACAATCGTAAATTGTAGACCGTAGTTTCAATAGAATTAACGCGAGAATA
TTTCTGGAGTAAAAAGTCTGAAATAGAAAAATAGGGACCATTAAAAATTGTCAGCCGATAGCCAGAATAT
GTTACAAGTCAACTGTGATTTGTGTTTGAAGTTTTTTAAATTGATTTTTAACTGAAACTTCTACTTTACA
GCGTTTGGAATCAAGTAAAGATATTTAATTCAATTCTTGCGAACATTCGCCTAAAGTCTCACACGTCATT
AAACTGGATTTTGTAGCTTACAAAAACTTCTCCGTCCCTACTCCACCGGGGTTTCTGAAAGAGCCATCTC
AGAACGACTTC

>Nut Promoter -1155 to -1 (Shimai, Kitaura et al. 2010)

Development • Supplementary information

ATCTGTTCTAGGAATCTTTTAACTCGGCGAGCCTGTGTTTTCTTTTTAGACTATAATACCCTCCAATAAC

AGGGTAAGATGCATGCGAAAAAAACGATTGATTTTGTGCATATAAAATATTTATAATAGTCTGGGGTTAT

GTGAGTGAAATAGCTGGTTATATCTAACTGCAGCCGGAAATACGTGCTCTTTGCAGAAAGGAAAATCCAT

TTCTCGTGTATTGCAACATACGGTATAGTAGGTTTAGATGGGTCACCTTTACCACAAATAACATCCAAAT

ATCCTGAGCATGTTTTAAACATTTAACAACGGTATATGTGGAAGTTGCGAGAATACGGTTTGATAATTCA

TTAAATATGCTTTGTTTACTACAAAATGGGACGGGAAAATAAAATAAAAAGGTGTCCTATCTTCCCCCAT

CCTACTATATATATCATATTTTCTAATCCTGTTTTAGCAATTAACAACGCTCGTTTAAAGTCGTGGGTAT

ATGCGGTTACATAAATTTAAAAATATTATTTGTTTACTTCAAAATAGAACGATAAAAGCGTGTATAAACA

TGTTGTATCATCTTACCCCATCCTGCTACAAATATAAACAATTTCATGATAAGAAAATGCACATGTTTGT

TATGCCGCGTGTTGTGTACAGTTCTGGTTAGGATTTCAGAACACACACACAGAAAATCGGTCGTGCCTGC

TGCTGCAACTAGTGCGACACCTCAATAATTATGAGACTGCCTCAGTGCATTCCTATCAACGTGTTTTGCG

CTCGTATCACCCAAAAAGCGTGCCTCGTGCTGCCTACACGGTGTGGCTACATAAAAACGTCGCGTGTTTT

ACTGTTCCAGTTCGTTTTTGTATTCGTGGAAATAGGTTCGCATTTTTTATTAAGAAAGAGTTTGGTTTTC

GAGGTCACTGAATTGCAATTAGAAGGCATTTAATAGTAGCACTAGGACACTCTATTCACTGCGTTGTCTA

AATCCATGCGAAACAACAAAATAAGTCGCAAGACATGCTGTGCGTGTATTGTTTTTTTGAAACCCCGTCG

CTTTGTGAAAATCTGGTTGATTATTTTTTCGTACCAGTTTTACAGTTTAAATACGACTGTGCTTCAGTTT

TTGTTAGTATTGAGTTGTACACTATATCAACAAC

>Gli -3339 to +57 atg start codon

GGTGATGACTGGAACCGATGACCCGTGACCGTGCGCCGTTATGCTCCGTTGCTCGCGTCAGTCGACAGCT

AATGCGTTAAGCAGTTAGCCGAAATCCTTTGCACACTAAGTCTGCTGGAGATTCGATCGAAAATAATTTG

AATATTTTCACAGCCCTTGCTTGGCTGATATTATATTACCAACCAGCAAAGCATCCAGTTTATCCTAGCG

AGTACCACCGCCGCAGGCCGTATTTGGAATCAACGTGAACTTGATACGTTTGCTAAGATATCGTAATATT

ATCAAAACTGGGAATAATGCTAGGATATATATAATCTAAATATCTTCGCTTACCATTTTTGGAAAACTTA

CGGTAATGTCATGTGTTGTACAAGAAATGTCGAAAGCGTTTTGGTATTTGGTAAATTAAACAAGGTGAAG

TGGGATAATTATAACACTTAACATTTGAAAATAACTTTCCCTCTTAAATACCACCTTGATCATAATAGTT

CGACAATTCATTTGAAGTCGAAACTCGCTGTAATTTACAACAGGTTTCCGTAATGAACAAGTGATCATTT

Development • Supplementary information

TTGAAGAGGAGGCGTGAAGTGAAGCTTCTCGAACTTCGACCTCAACAAAAAGTGCAACCGCCCAAAACGG

CCGGTATGCTGCCCCTTGCGGTCTCATATGTGGGCATTTGAAGGCCTAACACAGGCCTGCAATCAAAGAA

ACTACGCTTGCGTTTAGGAGATTTTCGGGCGCTGTCTTTTCCAGTTCCGCAGATTTTTTTCAGACAAAGC

GCGCTGGCCGTCACGCCTCACCGCACCCGTGTTACCATTGTAGCGTCACAAACCGAAGTTTTTTCGACGC

GCTCTGTTTATCGTTTATTATTTCGTCACGAAGCTGGCGCGGGAGCCTCCGATGCACTGCGCAAACGATC

TGTAGTAGTTAGCTATAAAACAAGCTTTATTTATATCTGTATTAAGGCTTTTCAATTTACAAGGCACATT

TTGTCGAACCAGTGTATTATATTAATCATTATTTGATAAGAAATTCGCCGAAAATGAAGAAGTTTTAGTT

CCAGTTGCAGAAAAGGTGAGTTATCACAATGCGGGTCTAAAACTAAGTGTTAGGCTACTATAATCAGCTG

TTGAGGAATGCAGTTTACGATTCCGATAGAAAAACATGTTCAAATACAGAGCTAGCAGTGTGTATAGGTA

TGTAGAGGTGCGTGTATGATGTATATTGATGCGGTAGTCTGGTGGAATGAGGTATGTAGGCCTCCCATCG

AAAGAAACGTTTTATTATTCAAAGACTGTTCGATCAAAGTCGAGGTAGAGGCGCCTTCGTGAATTGGATT

GAAATGAACCAGAACCAGCAGCAGCGGAATATTTCAGGCTCCCCCGGGGCCCGTTAATGGCCCTGCGGCT

TGTGCTCTGAGGTAACTCTGTTATTCCTACGCCTAAGCTAACTTTATAAGAGTATATGCGATTTGGGGTA

GTTCGTCCGTTTTATGCGCCATTACGACTGTTGGTAGACACCGTACGGCTGTGGCTGGTTTGTTGTTCGT

CCTCTAACTGCTCATTATGTCTTCCCGAGTGCCCGAGCCTCAATTACAGGATTACTCGCGAGTTTTAAAA

GCTTATCACTGGCGCACATAAGCCACCATCGTCACAGTACAGCGATTTCGACAAAAATGCCTGAGTGCTG

CCTCGTGGGCGCCCCTCTCTGCCCTATAACCGATCAAAACAGCATGACTCGTGTTGTATTTTGACATTGT

CGAGGGAGTTTTTCTGACTTTGTTGAAGTTTACGCTAAACGAGCTGAAGCAACCTTTCGCCTTTCTAACA

TAATTGCAAGCGCCCCGTAAAAAAAGTATTTTTGATTCGGAAACTCAAGGGCGAAGTGTTTTCAGGTTAC

GTTGGAATTTCCTGCTCAACGGTCTTGTAGCGAAACGTTAAGAGCCATTCTGCCTTTAAAATGTAAACCA

TGTATCCATTCAACCGGCGAGACACTGATACACTGATAGTCTATGCGGATTCCCACAAACAAACTCGGTT

CCCATAATGCCCAATGTATCGAATTCAAGTTCAGTAAGTGGCTTGTATTAAGTCGCATTGTGGTAGGGTA

TTAAGTTACAAGCCAAGTTCAAATACTTAAACCAGGACCACATCTAAGCGCATTTGACCGAGATCGTATC

AACTTTGGTTGATCAACGGAAATCGGGCCAATTCAAGTCCGTTAAGAATCCTCGCTTCAATAAATCTGTT

ATGTAACGTTTCCGTATCAAAAATCTTTCAGGATATATCGCTAACGGCCTCCTTCACCCCTATGGCTAAC

TATTTTTGGCTAGTAACGGTATTTTTAATAAAGTCTCTCGCCCCAATAGAAATGTTGACCCCTATCTGCC

Development • Supplementary information

CCACTTATAAAAGCGACGATCGCTGGGTAATCAAATAAGTGGAGTCAAGATCGGGGTCAAGATTGGGGTC

AAAAGGGTCAAGGTCAGGCGTGACCTTTTGGCTAGCTGGTGACACAATCAAGCCAAATGTGACAACAATT

GAGAATTTAATTAATAGAAATTTAGGATCGATGGTTGATTACGTCATACATCATCATACCTCGTGACGTC

ATAATTACGTCAGCTTAGATAGATTTGTAAAAGCTGGTTAGGTAAAGTTTTCCAAGCAAGAAGATACGAA

AATTATAAAATTGTTTAGTCAATCAAAATAGAGGTGTCAATTATAGCAATTACAATTTTCTGGTTACAAT

TTTTGGCATTTCCTTGAACCTATTTTCATATAGTGCCGTTTTAATCTTCGTTGGAAGTATTGAACATATT

AAAACTAATTAAATGCGGCATTTCTTGGCAGCGCTTGCCCTTGTAAGACAACCCGCAAGATCGTTCTATT

ATCTGGATGATGCCAATAATTTCTGGGTCGTTTCTCTCGAATTTCTTCCCAGAATGCGACGTCGGGCGTT

GATGGAATGTAACCTATTCTTGTAGACCGCCATTAATTTGACACCTACTGTTGCCAGGGCCTTGTCCCGT

AGACTTGCGGATTTTGCAGCTTTTGACACAGATTTGACTGTGGGAATCTTGCATGCCTTTAAACGTGGTT

TAGTTGCTCAATGTACCACAGCCGCGAATGAGTCAGTATCGAAGCCTGGCGAATGGAATTTGAGTTCTAT

CCATGTGGCGTGCCGCATCGCAAATCCGCGGCGTCTGGTTTTCGTCCCTGTTATCTGTACACTGTTGTAT

GGTTCTGTGGAACGTCCATGTGGTCAGTTTAGAATTTCAAGACTGATAACAATTTTGGTATTTTTTAGGG

GTAGGTATGTCGTAGATTTTACATGGAGAACGCGGCAAAAAGCGACGTTCAAGTTCTGTGTCA

>Vanabin4 promoter -1793 to +162 atg start codon

TAATACAATAGGAAAGAAACGATGGTATGTCGCTACTGTTGTTGCAATTCTTTATATTACGATCAGTTTA

GTCAACTTGCCGAAGAACTGTCTTTTAAAGCTCCGTTTTCGTATGAATGAATTTAATTTATTACGCCCGA

CGAGGCGGGACAGCGACAAGCGTTGTTGCACAGGGTTTGATTTCATATACCTTGAACCCAATTGCGAACT

TTATAACTTGTTTGTACATAGGTAAACGATGCCGCTGTGGCCGTTTAAAAAGTGTACACTTTTAGGACGC

GCTGTATATTATATTTAGCGGAGGACTTGCGCCCCCTGGCGGCCAGTTTTTTTTAATTTAGAAAAAAACG

AGCGGTTTAAAATTAGGTTTAAGTTAAAAATACAGAGCCAGATACCGCAGTTGGAAAAAAACTGAAAGCC

AATACATTGTAGTTTTACATTGTTTTTTTTTGTTTTTTTGCAGGATCATAAAATTTCGTTTGGAGCTGCG

AAAAAATATTTCAATGCACAAATTGTTCCGATGCTTAAAAGTACGATGAAAAACACCGATCTATATCATT

AGACATCAAAATCATGAGAATAAATGTAACTTGCTTTATCCTGGCGTGGCCGGAACACGACAGCCGTTGT

AACACGGACGTATAGGCTACACTCAATGTCAGCTTATGAGTAACCATGTATGTTACTTAGTGAGAGTATA

TTATATACACTATCGTCTTCGCCAGTTTAAGACTACTGCTATTAACTTTCTTCAATATATTTTCCGCAGC

Development • Supplementary information

AGGTTTTGGGATTCTTTCGTAGATGTGCGTTAACGTTTAATGTACGCTTGTGGAACTTGTTTGCCAGTAA

CAATGCAATTTAACCAATGTCAAAACAAGCGCCTGCATTTTGCAAAACAAAACCCAGCGGCGTCTGAGCG

GTTCGAGCTCGAGCACCGCAGACTAAGATTCTGTGGGTATGTTTTCGACTCAATAACCCCCACCAGCTCA

AAACAACAATATTTTCCTCCTTTAATTCTCTTCAACGTTTCTTTCTTTTATTCCACCACCGACGTGAAGA

GAGAAAAAACGCAGGAGGTGGTTCGAACATTCTAGCAAACGCGGGTCAAACGAGGCAGCAAGACCAACGT

TTTTTTTTGTGGAAATATACTCGCCTAAAACAGCATCTTCCTTCACGGCAAAATATGATTCTTTTTAAAT

AACAGAGATGCAATTTTGGATGAAAAATATATTCAACAATATAAAAAACGTTTGGAATTTTGTCGGTTGG

ACATTTCGTAGCATTTGATCCTGAATGTTATTTCGTTACGAGAATCGTGCAACTTATAAAGGAGACCAAG

GACAGATTAATGAAATATATTACAGTTAGCATGTGAAACAAAGCGAACTCTCCCATATACCGTGTTGAAT

AAGGCGACAAACAGAAAAGTTCAATTGAGGCCAATTTATGAACGAAAACGTCAAAAACAAGTGCATTGTA

CCCCGTTGACTGATGTTTTACCAAAAGACAAAAAACGAACCATTTTCGCTTCACTGAAGCTCGACGCTTT

GAACGCGTGATCTTTTGACAAATTCCATCATCAGCAGAAACAGCTAGCGGAATTGGAGCACAAGGTTAAT

CCAACCGTGCAGAGCGATTTACTTAGCTTGCGTTTTGCAAGGAAAAGCTTCATTTGGAGTCAACAGGACT

GTAAGTAGTTGCTGTTGTTTGTAGCTAATGCTAATTGTTAAACAGGGGAGCGAATGTATTTAAAATAATA

ATTATTTTAATTATCTACATGCAGAAGCTATCAACCGATCAAAATGAAAACGTTCTGTGTTGTTACAATT

GTACTTGTGCTTGCATCGGTGTGTGTTGATGCCCGTGGAAACCGCCATCATGGAGGATTGATGGGAACTG

GGGTGCCAAGGTGCCTGAAAACGTGCAAAGATGATTGCACGGAAATGAAACCTTGCGCTTTAGCT

>Eef1a promoter -1955 to -1

GTGACGGGAAAACGATAGTCGTTATAACACGAGTATTCGTACACCTCGTGCGAGCTAACGAGCTACCATA

TATGTTGTGGGCGAATAAAGGTTTTATAAATATAACATTGGTTTTATAAATAAAACAACGCCATTTTAAA

GTCGGTTACATAATTCTGTAACTAGTTCAAATTGAACGGTAAACGTAAATAAAAACCTTGACCGTCTTAC

CCAATTATATAAAAACACTTTGAACGCTTTTTAAGATGGAAGGGTATGGCCATGCCTAGATAATTCTGTG

GACCATCTCACCCCAACCTATTACAGAACGGTCGTAATAATGAAAATGGGTACCATTTTTAGGCATATAG

ACTGATTCCTCCTTTCTAGAAACGTAAGCAGTATACACAGAAAAAATGAAGTGTGATTCTGTGCAATTAA

ACCGTTCTAAATTCATAGCCGACTGAATTTCTAATTAAGTGAATGTCTGACCTAGATTTATTGTTAAGTT

TAGCACCAAATCTGAGCCAGCGATAAGCAGTCTAATTAAATTGGCTGCTGGCGATAAAATAGGTCATCCT

Development • Supplementary information

GAAAAATCGTTTGCGCCTTTATTTAAAATATAGTAGAGTGGGGAAAGACGGGACATCTTATCGTTCTATT
TTCTCGTCCCATTTCGTAGTAAACAAAGAACATTCAAAAAATATAAAACCATAACTTCAAAACTTCAATA
GACCGTTGTCAACTGTTTAAAACACAATAAGAGAATTTGGATATTATGTGCTAAAGGTGTCCCATCTCCC
CCCACCCTACTATATCTGTTTATAGTTCTGTGGGGTAAGATGAGATACCGTTAACACCTAAACATTTTTA
CTTTAAACAATCAACCACGTTTTTTATAGTCGTAATGGACATGTGGTTACATAATTCTGAAAATATTTTT
TGCCCCCGACCAAAAGACGCGAAGAGTAAAAACATGTCTCAGCTTATATTCCCCACATAAATATATTTTT
GTACTGTTTGGTGAATTTATAAACTTATATTACCATGCATATACGTTATGTTACTGGTATTTTCTCAGTA
GGCAAATTCATTTGTCCACGTTTTATAGGTTTTCAATATTTATGATTTTTAAAATGCTAAAAATGTGGGA
GGGGGGTTGAAAGTACAATACAAACACACAAAACAACTCAAACTAAAGATTTATAGTTATGCTAATTCAC
CTACACAATATAACAAGATGTGTAATGCAACCATGTGTTTATGATGAGCGCTAACATATTTTGTAACCAC
TCAAATTCCCCGCCACACGAGGATAATGAATAGGTGACTCTGTAGTCTGTACATCTTAGACTGAAATAAA
GATTATAAATCTACGAAATAAAATAATTTCTGCTCACTGATTATACTTCTGTTTTATAGATTAGAAACCG
TTTCTAATAAATGACCTAATTCGCTATACACACACGCTGTGCGCGAGATAATCATTCTCGCACCCCGTTT
ATTGTGTTAAAATTGCCGCCTAGATTCACAAAGCGTGACGGCTAGAGCCAGCAACGTGTCGCCTTCAATT
ACGCAACATCCGGGTTGCGCAATTCTGGATATAAAAGAACTAACAAAGATGACGTAGCTACCTTTTTCAG
TTCAGACTTACGAAAGACTCACGTGTCGGCGGTCTACTTGTCCTTTTCGAGCTGTGGCAATTTGGTGAGT
GGTTCTATCTTATATCTGAGTACATCTCTAAGGAATTATAGTTTGATTAGTTAAGTTTTTATTGTTAGGA
AAGATGAAATCATTAGGTTTTACTTAGTTTAAGTATGTTAGTACTGGTTAGGCGTTTGAATTATTGAAAA
ACTCAGTTCGTTAACTGTAGTAGTTCTGGTAGCTTAGCAAGTATACCCTGTATACGCCTTTTGGCTTTTT
AACAATAACTTAAACTTATTTTACAGCAAATTTCTGTGCATTCGGTTAACCCCAACCTTCCAAA

>GRIK intron 1 + bpFOG promoter (bpFOG from Rothbächer et al 2007(Rothbächer, Bertrand et al. 2007))

CCGACTGCATCCGAGTAGGTAAGTTGACTTCGTTTATTTTTATTTATTTGTTGTATTTGTTACATAATAT
AATATAGTAGGGTGGGGAAAGATGAGACACCTTTTCGTTTTATTTTGTCGTTCCGTCTGGTAGTAAACAA
AGAACATTTAAAAGAAATAATAAACCGTATCCTCACGACTTCCATAGATCGTTGTTAAAACCCAATATTG

GACGGTATATGCTAAAGGTGTCCCATTTTTCTCCACAGTACTAAACAGTAAAGCTTGCCAAGTTTTTAGT

TTTTAATGTATCGTAACTGAACAGTTTCTGTCAATTTTTGACGTACTATACCCAATCTAAACAGCAAAAT

TCATACTATTAACAGACCGTATGAATAGTATGGTTTTGTATGGCGCCATCCCGTTCACGCTGCTTTAACC

TTTGTTTTAAACACTTACCAAGATACGTATATGATTCAAAAATAGAATATCGTATAGGGTGGGGAAAATT

GTAACACTTAAGCTCGTACTCTCCCATATTTAAACACATTTAGTATTTAATAAAATTATTCGGCTCTGGC

GCGCGTTTTACGGCTGTGAAAATACTATCGAATAATCGTATAATATATAACATTTTTATTTGCTTATTAT

AAAATGGGAGCCAAAAAAATATATCGTCCCACATTATTTAGTGCCCTACTGTATACCCCATCCAAAAGAA

CAGTTGTATATCCATTTACTTTTTCCTATTCTCAAATCACGATTTATTAATTGTAATCTCGACATAAAAC

GTTTGTATTCAAATGTCAAAGCGAGGATCACGTCATGTAAAATAAATAAATTCCGCCGCCGTAGCACAGT

GATTAGTAAGCCTGCCTATATACGTTATAACCATATACGTTATGGGAAGGCTCGGCGCTGTTAAGCATTG

TGGGCGTATATGTGCCGTATAGGACAAGCAACGGCGATCGCTGTATTTCATGCAGTAATTACTAATGGGT

TGTCTAAATTATAAGCCATATATAGAAAAAAAAACAAACTCACAAAGTTAGATATTTGTCATATCTCGTG

AGCGGGCGCGAGGTGTATGATACAAAACACCGGTGTTATATAACTACTTTCGTTTTTCGGCCGCGCAATT

GAATAATGCAAGTTACATTTTTCATTAAGTCATTGCATTTAAATTAAGCCATGTTTGTATTTTGTCGAGT

ATTGGTTACTGACTATGCAGCATATCGCCTTTGCATTTCCAATCTTGGAACGTTAAAGTATAAGAATCAT

GGGTGACATATATTCAATATAGCGTGCTGTAATGACGCACGAAACGTCCCGGTCAAACGGTGCCGCTCCT

ACCCCACCTTCCATCATTATTATCACGAATTATTTCGCACTTTTATGTAACTGCATTTATTAAGGACCC

GTCGTCCAATGACGTTTAGAATTCGCCTGAAAATTATTTGAAATATCGACGCCCAGCGAGTGTTATATCA

TTCAGTCACGGTGTCGGTTTAGCGTCTCTAGAGCTTTCCAGCGTCTGCTTTGCTGCTTTAAACGTAACAA

AGCGTGTATAAGCAGATTAAGCGACTCAAAATTTAATTTGCGTTTCTTTGAATTCGAAGCAAAAAATCAT

TTTAGTCTTTTTTCTTCGACTATGTTTAAAGTGTTTTCTTAATTTTGCTGCCGACGCACTTTTATGGAGT

AAGAGAAAAGCGGTTAATTTTTAATGCGCTTACGGCGACCGCAGAATGATATTTTTAACGCGGTCGACGT

TAATAAAAACTGGACGATTTGATTGCAGTCAAAATTTATTAATCTTACTATATCAAGATAATAATAAAAC

CAAAGACCAAACAACCGAGGAAGAATCGGCAAAAAAATGAATCAATACCTCGCTCGGCTTAATTTGTCCG

ATTAAGTAGCGGTGCAGTAGGAGAGTTGTACTTTAATCGGACTTGCTTTAATAATTGATCAGCTCAAGCG

GGATGGAAGACTGCCAAGGCTGGATCGGTATAGTCACAGTAGTCAACTTTGGAAAAAGGCGACGAGCTGT

Development • Supplementary information

CGGTCTTTTTCGGGAAATTCGACCGTTCTGATTTATTCATTGTTAGCCAAGCAGATTTCGTTGACCGGCA

ATCCCCTTTAAATCGATCGTTACCTTGTCCCTTTAATAGTGGTTGTGGAATAGGATTCTCAGACCTTAGC

GTGATGTCTAATTCATAATCCTTGCGTCCGACAGCAATTTAAAGAGGAAAAATTCGATTTATTTCGAGGT

GTGGCTCAGCATGTTCGATTGTGTTATCTGCGGCTCGTATTGGTTATTCCATTTACACCACATTCAGCAA

TAGCCTTTAACAAAATATGTTTAAGAATAATAATAACAGCACGCACGTTGCTAATGAATTTGTAAACTAG

TTCTATTTATAGATCACTGTTATATTTATAAATTATTGCGGTCAAATATAACACAAGTCTAAAACACCTT

TAATCCGTCAGTTTATTTAGTATAGGTTGAGGTAAGATGGGACATGTTTTCATTTCCTTTTTTCTGCCCA

TTTGGTAGCAAACAAACCATATTTGCCGAATTATATAAACGTAGCCTCGCGCCTCTTAAAGAACGTTGTT

AATTGTTAAAAAGTAGATAAGGAAGTCTGATTTCGCCGTCACCCTTAATATTGCATTATGTCGACGTTTT

GCCGTATAGTTAGGTCACTGATAAGCAAATTCAGTATTGATACATTATCACATAGCTAATTTCCTGCATA

CAATTTTAGGAGGAATTTTTCGCAAGAACATCGCAACCTCGAGTATCTGCAGGTCGACTCTAGAGGATCC

GGCAAAGCTTCGTGTATTGTACCGGCCCATTGTCAATCATGCAAACTTGATATTATATTGACAAGAGAAG

AAGGCAGTTTAAATTAAAACTCTAAAGTAGAGAGACATTAATCTCAGCTGACAAGGCAGGTGGTCACAGT

AAGTTCATTTAAATAGTTGGCCAACAATAGCCTTTCCAAGAAAGTATTTTTGTTCCAGGTCTATACAAAA

ATAACACACAACAT

>Cas9::CionaGeminin-Nterminus (from Song et al. 2022(Song, Yuan et al. 2022), which originally described it as having the Geminin sequence from human instead)

nls::Cas9::nls (described in Stolfi et al. 2014)

Ciona robusta Geminin N-terminus

ATGGCTAGCCCCAAAAAGAAGAGGAAAGTGGACAAGAAGTATTCTATCGGACTGGACATCGGGACTAATA

GCGTCGGGTGGGCCGTGATCACTGACGAGTACAAGGTGCCCTCTAAGAAGTTCAAGGTGCTCGGGAACAC

CGACCGGCATTCCATCAAGAAAAATCTGATCGGAGCTCTCCTCTTTGATTCAGGGGAGACCGCTGAAGCA

ACCCGCCTCAAGCGGACTGCTAGACGGCGGTACACCAGGAGGAAGAACCGGATTTGTTACCTTCAAGAGA

TATTCTCCAACGAAATGGCAAAGGTCGACGACAGCTTCTTCCATAGGCTGGAAGAATCATTCCTCGTGGA

Development • Supplementary information

AGAGGATAAGAAGCATGAACGGCATCCCATCTTCGGTAATATCGTCGACGAGGTGGCCTATCACGAGAAA

TACCCAACCATCTACCATCTTCGCAAAAAGCTGGTGGACTCAACCGACAAGGCAGACCTCCGGCTTATCT

ACCTGGCCCTGGCCCACATGATCAAGTTCAGAGGCCACTTCCTGATCGAGGGCGACCTCAATCCTGACAA

TAGCGATGTGGATAAACTGTTCATCCAGCTGGTGCAGACTTACAACCAGCTCTTTGAAGAGAACCCCATC

AATGCAAGCGGAGTCGATGCCAAGGCCATTCTGTCAGCCCGGCTGTCAAAGAGCCGCAGACTTGAGAATC

TTATCGCTCAGCTGCCGGGTGAAAAGAAAAATGGACTGTTCGGGAACCTGATTGCTCTTTCACTTGGGCT

GACTCCCAATTTCAAGTCTAATTTCGACCTGGCAGAGGATGCCAAGCTGCAACTGTCCAAGGACACCTAT

GATGACGATCTCGACAACCTCCTGGCCCAGATCGGTGACCAATACGCCGACCTTTTCCTTGCTGCTAAGA

ATCTTTCTGACGCCATCCTGCTGTCTGACATTCTCCGCGTGAACACTGAAATCACCAAGGCCCCTCTTTC

AGCTTCAATGATTAAGCGGTATGATGAGCACCACCAGGACCTGACCCTGCTTAAGGCACTCGTCCGGCAG

CAGCTTCCGGAGAAGTACAAGGAAATCTTCTTTGACCAGTCAAAGAATGGATACGCCGGCTACATCGACG

GAGGTGCCTCCCAAGAGGAATTTTATAAGTTTATCAAACCTATCCTTGAGAAGATGGACGGCACCGAAGA

GCTCCTCGTGAAACTGAATCGGGAGGATCTGCTGCGGAAGCAGCGCACTTTCGACAATGGGAGCATTCCC

CACCAGATCCATCTTGGGGAGCTTCACGCCATCCTTCGGCGCCAAGAGGACTTCTACCCCTTTCTTAAGG

ACAACAGGGAGAAGATTGAGAAAATTCTCACTTTCCGCATCCCCTACTACGTGGGACCCCTCGCCAGAGG

AAATAGCCGGTTTGCTTGGATGACCAGAAAGTCAGAAGAAACTATCACTCCCTGGAACTTCGAAGAGGTG

GTGGACAAGGGAGCCAGCGCTCAGTCATTCATCGAACGGATGACTAACTTCGATAAGAACCTCCCCAATG

AGAAGGTCCTGCCGAAACATTCCCTGCTCTACGAGTACTTTACCGTGTACAACGAGCTGACCAAGGTGAA

ATATGTCACCGAAGGGATGAGGAAGCCCGCATTCCTGTCAGGCGAACAAAAGAAGGCAATTGTGGACCTT

CTGTTCAAGACCAATAGAAAGGTGACCGTGAAGCAGCTGAAGGAGGACTATTTCAAGAAAATTGAATGCT

TCGACTCTGTGGAGATTAGCGGGGTCGAAGATCGGTTCAACGCAAGCCTGGGTACCTACCATGATCTGCT

TAAGATCATCAAGGACAAGGATTTTCTGGACAATGAGGAGAACGAGGACATCCTTGAGGACATTGTCCTG

ACTCTCACTCTGTTCGAGGACCGGGAAATGATCGAGGAGAGGCTTAAGACCTACGCCCATCTGTTCGACG

ATAAAGTGATGAAGCAACTTAAACGGAGAAGATATACCGGATGGGGACGCCTTAGCCGCAAACTCATCAA

CGGAATCCGGGACAAACAGAGCGGAAAGACCATTCTTGATTTCCTTAAGAGCGACGGATTCGCTAATCGC

AACTTCATGCAACTTATCCATGATGATTCCCTGACCTTTAAGGAGGACATCCAGAAGGCCCAAGTGTCTG

Development • Supplementary information

GACAAGGTGACTCACTGCACGAGCATATCGCAAATCTGGCTGGTTCACCCGCTATTAAGAAGGGTATTCT

CCAGACCGTGAAAGTCGTGGACGAGCTGGTCAAGGTGATGGGTCGCCATAAACCAGAGAACATTGTCATC

GAGATGGCCAGGGAAAACCAGACTACCCAGAAGGGACAGAAGAACAGCAGGGAGCGGATGAAAAGAATTG

AGGAAGGGATTAAGGAGCTCGGGTCACAGATCCTTAAAGAGCACCCGGTGGAAAACACCCAGCTTCAGAA

TGAGAAGCTCTATCTGTACTACCTTCAAAATGGACGCGATATGTATGTGGACCAAGAGCTTGATATCAAC

AGGCTCTCAGACTACGACGTGGACCACATCGTCCCTCAGAGCTTCCTCAAAGACGACTCAATTGACAATA

AGGTGCTGACTCGCTCAGACAAGAACCGGGGAAAGTCAGATAACGTGCCCTCAGAGGAAGTCGTGAAAAA

GATGAAGAACTATTGGCGCCAGCTTCTGAACGCAAAGCTGATCACTCAGCGGAAGTTCGACAATCTCACT

AAGGCTGAGAGGGGCGGACTGAGCGAACTGGACAAAGCAGGATTCATTAAACGGCAACTTGTGGAGACTC

GGCAGATTACTAAACATGTCGCCCAAATCCTTGACTCACGCATGAATACCAAGTACGACGAAAACGACAA

ACTTATCCGCGAGGTGAAGGTGATTACCCTGAAGTCCAAGCTGGTCAGCGATTTCAGAAAGGACTTTCAA

TTCTACAAAGTGCGGGAGATCAATAACTATCATCATGCTCATGACGCATATCTGAATGCCGTGGTGGGAA

CCGCCCTGATCAAGAAGTACCCAAAGCTGGAAAGCGAGTTCGTGTACGGAGACTACAAGGTCTACGACGT

GCGCAAGATGATTGCCAAATCTGAGCAGGAGATCGGAAAGGCCACCGCAAAGTACTTCTTCTACAGCAAC

ATCATGAATTTCTTCAAGACCGAAATCACCCTTGCAAACGGTGAGATCCGGAAGAGGCCGCTCATCGAGA

CTAATGGGGAGACTGGCGAAATCGTGTGGGACAAGGGCAGAGATTTCGCTACCGTGCGCAAAGTGCTTTC

TATGCCTCAAGTGAACATCGTGAAGAAAACCGAGGTGCAAACCGGAGGCTTTTCTAAGGAATCAATCCTC

CCCAAGCGCAACTCCGACAAGCTCATTGCAAGGAAGAAGGATTGGGACCCTAAGAAGTACGGCGGATTCG

ATTCACCAACTGTGGCTTATTCTGTCCTGGTCGTGGCTAAGGTGGAAAAAGGAAAGTCTAAGAAGCTCAA

GAGCGTGAAGGAACTGCTGGGTATCACCATTATGGAGCGCAGCTCCTTCGAGAAGAACCCAATTGACTTT

CTCGAAGCCAAAGGTTACAAGGAAGTCAAGAAGGACCTTATCATCAAGCTCCCAAAGTATAGCCTGTTCG

AACTGGAGAATGGGCGGAAGCGGATGCTCGCCTCCGCTGGCGAACTTCAGAAGGGTAATGAGCTGGCTCT

CCCCTCCAAGTACGTGAATTTCCTCTACCTTGCAAGCCATTACGAGAAGCTGAAGGGGAGCCCCGAGGAC

AACGAGCAAAAGCAACTGTTTGTGGAGCAGCATAAGCATTATCTGGACGAGATCATTGAGCAGATTTCCG

AGTTTTCTAAACGCGTCATTCTCGCTGATGCCAACCTCGATAAAGTCCTTAGCGCATACAATAAGCACAG

AGACAAACCAATTCGGGAGCAGGCTGAGAATATCATCCACCTGTTCACCCTCACCAATCTTGGTGCCCCT

Development • Supplementary information

GCCGCATTCAAGTACTTCGACACCACCATCGACCGGAAACGCTATACCTCCACCAAAGAAGTGCTGGACG

CCACCCTCATCCACCAGAGCATCACCGGACTTTACGAAACTCGGATTGACCTCTCACAGCTCGGAGGGGA

TGAGGGAGCTCCCAAGAAAAAGCGCAAGGTAATGGCCACGAAAAATATTCTTCAAAATATAAATGCACAA

TGGAAGGAGAATGACAACAGATCACCAAGTAGAAAGCGACGGTTAGATGACGTCACTGAAGAATCACAAT

TACCTTCCACGACCAAACGACGTCATCTTCAAACAAATACAAACGTTGTAAATTCCACAGGATTGAAACA

AGGCCTGACAAATGTGAAAAATTCAATAAATCCAAAGAACAAATCAATAAAAAATTTCTTTTCTGATATT

CCACGTGTGTCATGTACTAAATCTGAAAAGATTCAAATTTTTAAAGAAGCTAAGAAAACTCCAAAAAAGA

ATGCAACCACTCAGACAAGGAGTGAAGCTGAAGAATTGGTCTGCAGTGATCAACCCAGTGAAAAATATTG

GGAACTCTTAGCCGAGGAGCGAAGGAAAGGGTTGTAA

>Pax2/5/8.a tv1 “rescue” coding sequence, sgRNA sites mutated

ATGAACTGGGGATCAGCAATGGCGGTTGGACCATCCAGTGTGGGACATCCATTCGTGGGATCAGGAATGG

CGGCCTCACTCACCCCTTCAAGATCAGGGCACGGTGGGGTGAACCAGTTGGGCGGGGTTTACGTGAACGG

CCGACCATTACCCGACCAAGTCCGACAACAAATtGTcGAtCAgGCtCAtATAGGGGTTCGACCTTGcGAt

ATaGCacgcCAgctCCGGGTGTCGCATGGTTGTGTAAGCAAGATATTAGCAAGATATTACGAGACAGGCA

GCATCCGACCTGGTGTaATtGGaGGttccAAgCCaAAaGTtGCaACtCCaagaGTtGTGGAGAAGATATG

TGATTATAAACGACAGAATCCAACTATGTTTGCTTGGGAGATACGAGACCGATTGTTGAGTGAGGGAATT

TGTGACCATGATAATGTACCCAGTGTTAGTTCGATCAATAGGATTGTCCGAAACAAAGCTGCAGAAAATG

CAAAGTCGCACCAACAACTCATGGTCCCAATGACGCCGTCATCGCTGGACAACTACAGCGAGCACATCGG

GCAGGTGTCGCGCATGAACGGTTTCAATAGATTCGCTTCATCGACCACAGCATTACCCACAATGCAACAA

TCAAGTCATGTGACTTCTCAGATGAATTTCGTTCGTAAAGAAAACAAGGGATTGGATTATTCGTACGATT

GTCGCGGTTCAACGAAcTCACCCAACGTAGCTACTTACCCAGTGTTACCTCACAATCAACCTCGAGCTAA

CTGTGATGTCACAATCAGCCCAATGACATCACAAACCAACACTGCAAACGCGACAGTTTCACCCAGCAAC

AGTGGGGGTTACTCTGGGTCAAGTTTTGCCCCGATCACAACCGCGTACGCTCCGACAGGTGAGTTTGTGG

ATTATGGATATCAACAATACAATCAACACTGGAAGTTTGGCCAACAGCACCATAATGATAGCAACACTGG

TAAAGTTTTGAATCTTCGAGGTAAAGAACATCCGGCAACACTGGAGATGGTCAGCGCTCAATAA

Development • Supplementary information

>Pax2/5/8.a tv2 “rescue” coding sequence, sgRNA sites mutated

ATGAACTGGGGATCAGCAATGGCGGTTGGACCATCCAGTGTGGGACATCCATTCGTGGGATCAGGAATGG
CGGCCTCACTCACCCCTTCAAGATCAGGGCACGGTGGGGTGAACCAGTTGGGCGGGGTTTACGTGAACGG
CCGACCATTACCCGACCAAGTCCGACAACAAATtGTcGAtCAgGCtCAtATAGGGGTTCGACCTTGcGAt
ATaGCacgcCAgttCCGGGTGTCGCATGGTTGTGTAAGCAAGATATTAGCAAGATATTACGAGACAGGCA
GCATCCGACCTGGTGTaATtGGaGGttccAAgCCaAAaGTtGCaACtCCaagaGTtGTGGAGAAGATATG
TGATTATAAACGACAGAATCCAACTATGTTTGCTTGGGAGATACGAGACCGATTGTTGAGTGAGGGAATT
TGTGACCATGATAATGTACCCAGTGTTAGTTCGATCAATAGGATTGTCCGAAACAAAGCTGCAGAAAATG
CAAAGTCGCACCAACAACTCATGGTCCCAATGACGCCGTCATCGCTGGGTATCCACACAAACGGTCCGAT
ATTAATGGAACACGAGATTCGCGGAACGTACACGATTAACGATATATTAAGGTTACCCCACAACCCCCTT
CCCCCACATAACCCCCCCACCACTGAAACAGACCCAACACATTATAAACAAGCCGAAAATGGGATCCATT
ATAATCATAACGACAACTACAGCGAGCACATCGGGCAGGTGTCGCGCATGAACGGTTTCAATAGATTCGC
TTCATCGACCACAGCATTACCCACAATGCAACAATCAAGTCATGTGACTTCTCAGATGAATTTCGTTCGT
AAAGAAAACAAGGGATTGGATTATTCGTACGATTGTCGCGGTTCAACGAAcTCACCCAACGTAGCTACTT
ACCCAGTGTTACCTCACAATCAACCTCGAGCTAACTGTGATGTCACAATCAGCCCAATGACATCACAAAC
CAACACTGCAAACGCGACAGTTTCACCCAGCAACAGTGGGGGTTACTCTGGGTCAAGTTTTGCCCCGATC
ACAACCGCGTACGCTCCGACAGGTGAGTTTGTGGATTATGGATATCAACAATACAATCAACACTGGAAGT
TTGGCCAACAGCACCATAATGATAGCAACACTGGTAAAGTTTTGAATCTTCGAGGTAAAGAACATCCGGC
AACACTGGAGATGGTCAGCGCTCAATAA

>caBMPR1b coding sequence, gac (Q202D) constitutively active mutation

ATGGCGGCCGCAACCATGCTGGACAATGGACTACCTCGTATGCAGTGTCGTTGTATTGGAGAATGCCCGG
ACCACAAATACAACTCCACATGCGACCCGAAACCCAACGCAAAGTGCTTCAAAAAGTTGTACATTAATGA
ATATGGTGAAGAGGAGTTACGAGCTGGGTGTTTAGGTTCACAAGATGATTACTTGAACCAATGTCATAAT
AAAGCAAAAACAGAATTCGAAGTTCCAACTGCAGTGGCTTGCTGCAACAATGGTACAATGTGCAACGATT
ATTTAGACCTCGGGTTGCCTGAATATTATGATGAACCATCTGACACACCAGTTTCAGAAGCAAATTCGGA
TGTTATCACCGTTATAGCTGTTACAGTGCCAGTCTTCTGCTTTTTGTTTGGTTTAATAATAATGTTTTAT

Development • Supplementary information

TACATAAGATTATGCCGTAGAGAATCGCTACGACGACGTCAAATTGAGAATGAAAACAAAAAAGCTCTTT

GTCCTGTTTTATATGGAGATCGGGGAGACTTGGAAGGACATAATGAGATGATGGAAAATTGGTCCTCACT

TGCTGGTACATCTTCAGGATCAGGGATGCCTCTACTTGTACAGAGAACGATATCACGAGACATCGAAATT

CTTCACGAAATTGGAAAAGGAAGATATGGGACAGTGATGCTGGGGAAATGGAGAGAAGAGAAAGTTGCTC

TTAAAATATTTAATTCCAGTGATGAAGAAAGCTGGTTTCGAGAAACTGAAATTTACCAAACTGTGTTGCT

ACGTCATGACAATATATTAGGTTTCATAGCAGCCGATATATCTGGTGCTGGCTCATGGACTCAGCTGTTC

CTAATTACAGAGTACCACAAGCATGGCTCCTTATACTACTACCTACAAAACAGAGCCATCAACATAGCAG

AAGCTCTTAAACTTGCTTATACAGCTTGTTGTGGGTTAGCACATCTACATACAGAGATAGCTGGCACACA

AGGCAAGCCAGCCATTGCACACAGGGATGTAAAATCGCAGAATATTCTCGTAAAACTTGATGGACAATGT

TGTATTGCAGATATGGGACTGGCTGTCTGTTTCTCCAGATTACATGAAACGATTGATGTTGGAAAGCATG

ACCGCTCAAGAAGACAAGGTACCAAGCGTTACATGTCACCTGAGGTGCTTGCCCAGTCTTTTCACCCAGA

CTCCTTTGAAGCATACAAAGCATCAGATGTGTACAGCTTTGCTTTGGTGTTATGGGAAATAATCAACAGG

ACTGAAGTCAACGGATTTGCAAATGACTACCATCTTCCCTACCACGATGTGGTGGGTAATGACCCTGATT

TTGATGAGATGAGAAAAATAGTTGTTCTTGAAAATCTCAGACCAGAGATTTACAAACAGTGGCAAGCACA

TAAGATAATGTCAACATACACTACAACCCTGCAAGAATGTTGGTCCCCTCGACCCGAGTCTCGTCTCTCA

ATGCTCCGTCTCCGCAAGACTCTCTACTTTCTCCTCAATGGTGGACGAGGCCCAGTGGAGGCGGGAAGCT

CTGAAAGTGACCGAAAACCGTCTGCCTCTTCCAGCTCTTCTGTTAAAGAAGAGGAGCATTCAAGTTGCTA

A

>dnBMPR1b coding sequence, early STOP dominant-negative truncation

ATGCTGGACAATGGACTACCTCGTATGCAGTGTCGATGTATTGGAGAATGCCCGGACCACAAATACAACT

CTACATGCGACCTGAAACCCAACGCAAAGTGCTTCAAAAAGTTGTACATTAATGAATATGGTGAAGAGGA

GTTACGAGCTGGGTGTTTAGGTTCACAAGATGATTACTTGAACCAATGTCATAATAAAGCAAAAACAGAA

TTCGAAGATCCAACTGCAGTGGCTTGCTGCAACAATGGTACAATGTGCAACGATTATTTAGACCTCGGGT

TGCCTGAATATTATGATGAACCATCTGACACACCAGTTTCAGAAGCAAATTCGGATGTTATCACCGTTAT

AGCTGTTACAGTGCCAGTCTTCTGCTTTTTGTTTGGTTTAATAATAATGTTTTATTACATAAGATTATGC

CGTAGAGAATCGCTACGACGACGTCAAATTGAGAATGAAAACAAAAAAGCTCTTTGTCCTGTTTTATATG

Development • Supplementary information

GAGATCGGGGAGACTTGGAAGGACATAATGAGATGATGGAATACCCATACGACGTGCCAGACTACGCTTT

ATAA

>dnFGFR (dominant negative FGF receptor, Davidson et al. 2006(Davidson, Shi et al. 2006))

ATGATACAACTACAAAATACGTTTATTTTTATCGCTTTGACAATTTTTACTTCTGCTTCAACAACAAGCT

TAAAGAATGAAACCAAACCCCTCAACACAATTTCAACGCTAGCTGCTCAAACAAACATTTCAAACCCAGA

AGACGATTTGTTCGATACAAACGGAGCACCAAAAAGTGATACTGTGAATGCATCTACAACTACGGATCGT

CACAAGATTCCACGCTGGGTCAATGAACAGAAGATGCAAAAGCGACTTCACGCTGAACCAGCAGGTAACA

CTGTCCAGTTTAGATGTGCAGTTCAAGGTGCAAGACCAATCACCGTGGATTGGTATAAAGATGGGGAACC

AATCAAGAAGAATGGAAGACTGGGGGGGTACAAGTTCCGTCAACGCAACCAGCAAATATCATTGGAGTCG

GTGATAATGTCCGACCGTGCTAAGTACATGTGTGTGGCCCATAATAAGTACGGCTCCATCAACCATACTT

ACGAACTGGATGTAGTAGAACGTCTGGCACACCGCCCCATCCTCCAATTTGGATTACCAGCAAATAAAAC

TGTCAAGGTGGGAGAGGATGTGACCTTCAAATGCAAGGTCTACAGTGACCCTCACCCTCATATGGAGTGG

TTAAAGCATGTAGAGGTTAACGGGTCAAAATACGACCCAGTTTCTAAGTCCCCCTATGTGATTACATTGA

AGAGAGCTGGTATTAACACAACTGACGCGGAGATGGAAAAGTTAACATTGAAAAATGTTTCATTTGCCGA

CGCTGGGGAATATACTTGTCTTGCTGGAAATTCTATCGGGGTGTCTCATGTTTCTGCATGGCTCACAGTG

CTGCCAGTGGTAGATGAGAATGATGTATGGACCGAAGAAATCCCACAAGACACTCATTATCTCATATATA

TCTTTGGGGTTGTGTGCTTCATAATACTACTTGCGTTCATTGTGTATATGTGCAACTCTCGCTATCAAAA

TAAAGATCCTCCCCGTTTGATCCCGATCGAGAACCCCGACAACATCCCCCCCATGTCGAAGATGGAGGAG

CCGGTGATGTTGTTCGGGAACTAA

>dnEph.c (dominant negative Eph.c receptor, (Picco, Hudson et al. 2007))

ATGTTTTTTCTATTTATCGTCTTCTACTGCATTTCAATTGTAACTGGAGAAATACACGTTTTATACAACA

CTAAAGTTGCGACAAGTGATTTAGATTGGGCCTTACACCCCACATATGGAGCGTGGGAGGAATTGAGCGG

ACTCGATGTAGACGGTAACACAATACGTTACCACCAAGTATGTAACACAGGAATGGATGAACAGGACAAT

Development • Supplementary information

TGGGTACGATCCCCATTTATCGACGCAAAGTCGGCCCAGCGTATTTATATGGACATCGAATTTTCGGTTA

TGAAATGTGAGGAATGTCGCGAAACATTCGCGTTGTATTATTACCCGTCCAGTTCCGACACTGCTACCAC

TACATTCCCGCCTTGGAGAGAAAACCCCTATATCAAAATCGATACATTAGCAGCTGGTGAAAGATTCGAT

TCCGAAACCGTAGGTGCGGAGGGAATTAATAAGAAAACATTGGTTATTGGACCATTATCAAGACGCGGAT

TTTATATCGCTGCTCAGGACCAGGGCGCCTGTATGTCAATTATGAGTTTGAAATTGTATTATTACCACTG

TGAGGAAACGACCCATAACCTGGCTTATTTTCCGAACACCATATCGGGCGGCGGTATCGCTGAATTGGTG

TCGCAATCTGGAAAGTGCGTTTCGAACTCAGTATACGCGAATGAAGTTCCGAAATACCGATGTAACATTT

ACGGCGAATGGCAAGTCCCTACTGGTTCTTGTCAGTGCAGAGCTGGTTATGAACCAAATACACAACTCAC

CGCATGTACAGGCTGCATGGTTGGCAAGTACAAATCTACAAACGGCAATACACCTTGTCAAGTCTGCCCA

CAGCACAGCGTTACACATTCAACATCCGCTAGTCACTGCACTTGTGTGGCTGGACATTATCGAGCCGAAA

ATGACCCAATCAGCCAAGCCTGCACTCGGCCCCCCACTAAGCCTCGGAATGTGACTCATGTTCAAAACAA

GACTTCTCTCTTATTGTCATGGGTCCCACCTTCAACTACAGGGGGTAGAACTGATATATATTACAGCATC

TCATGTGAACTGTGCGATTCTGAGCATGAGAACTGTCAGCCCTGCAATGTTGATGTTCAATACAGACCAA

GCAACCACCAGCACACCACAACAACGTACATGGAAGTCTCAAATCTTAATCCCTTCTCATGCTATAAGTT

CAAGGTTTCTTCTTCAAATGGTGTTTCTAGGGTCAGTGTGGAGCCTGAACAATTTGAGCTAATAAAGATT

TGCACCAACGCCGCTGCTCCGTCTGCTGTCACGGGCCTTAAGTTTATTTGGATTGGGGAAGTTTCGGCAA

CTTTAAGTTGGTTGCCGCCCACACATAGCAACAAGATTGTTGGATATGAAGTGCAATTGTTCCAAAACAA

TCAGCGTTTAAAACTGACAAAAATTATGGAGGTTTCAACACCAAACGTTACAATAAATGGTTTGAACCCT

GGATGGAAATATGTAGTGATGGTACGAGCATGTAATAACGATGGGTGCGGCCAGTTCAGTGAAAAACTGC

AGCTAGTTACTTATGACAAAGGTTCTGAGCCTGAATCTATAGTGGAAGGCAGTACTACATGGATTGGAGG

AGTTATTGGTGGGGTTCTAGTTATCGTTATAATAATTATCGTCGTGATGATAAAAAGACGACAAACCGAT

AAAAAGAAACGAAAAGAAATCCAAGCCAGAACAAAACTCAACGAAAACACTCAACAACTAAACCAGAGTA

GCTTTTTACAAACCGCAGGGCGAACTTATGTTGATTACCGTGACCCCCACAATGGGGTAAAAGAAATCGC

AACCGAGATTGATCAAACGAGAATTAAGATCGACAGTGTTATTGGAAGAGGTGAGTTCGGTGAAGTGTGT

CGTGGTAAAATGTTGACGGGCAAAACAACGACTTCTGTCGCCGTGAAACGATTAAAACACGGAGCAAGTT

TAATTGACCACACCAACTTTCTACGAGAGGCATGCACTATGGCACAGTTTAAAGACCCGAATATTATTCA

ACTGAAAGGAGTGGTTACTAAAAGCATCCCCGCAATGATAATCACGGAATTTATGGAGCATGGTTCGCTT

GATAAATTTTTACAGGCTCGTTCTGGGCAGCTTACTGTTCTACAATTACTTGAAATGTTACGCGGCATCG

CAAGTGGAATGAAATATCTTTCATCAATGAAATATGTTCATCGAGATTTAGCTGCAAGAAATATCCTCGT

TAATTCTCAACTTGTTTGTAAAGTATCCGACTTTGGTCTTTCAAGAACTCTTGAGAATGACCCTCAAGCT

ACCTATACCACACAGGGTGGAAAGATTGCTCTCCGCTGGACAGCCCCTGAGAGCATCCGTTGTCGTCAAT

TTACATCAGCAAGTGATGTATGGAGCTATGGGATCGTTATGTGGGAAGTCATGTCTTATGGGGAGAAACC

TTACTGGGATATGAGTAATGAAGTTGTGACAGAGGTATTAGAAGATGGATACAGGCTCCCGTCACCTGAG

GGTTGTCCTACTCCTGTCCATAGTTTGATGTTGAAATGTTGGTCTTATGAACCGAAACGCAGACCAACCC

TGCTGGAAATTATTAAGACTTTGGATCATTTTATTAAACAGCCCAGCTCTCTTCAAGACGACATGGAAGC

TGATGCAAGTGCCCCGCTGTTAAAGCCAGACAGTCCCAACAGCATTCAAGATGTATCAACATTAGACGAG

TGGTTAGACATGGTAAAACTTGGAAGATACCGCAGGAGTTTTCATAACAACGGAATTAATGATCTTGAAA

GCTTGGCTCACATAAGTGAAAGTGAGTTGGACAGACTTGGCATAGCGTCACCTTCCCACCGCACCAGACT

ACAGGGAGGCATTAACACTTTACGACAACATTTGGTCGAAGTAAACGAAGTTCAATCCAGCAGTAACGCA

CCTTACGACGCCCACGCTTCAACGCTACCAATAGCTCACGGGAAACCAAACAATCCTGTTGCAGTTTGA

>caMEK (Razy-Krajka, Gravez et al. 2018)

ATGCCTCCTAAACGTAAGTTAAACCCGTTGAACCTAACACTTGAGGGGAGTTCGCCTATCCCCAATCAGC

AGGTCTCTCAGTTCATTAAGCAGAAGCAAAAAGTGGGAGTCATGGAAAATGCTCAGAACTCTGACTTCAC

AAAGAAAGGAGAACTGGGTGCCGGAAATGGGGGTGTTGTTCATCTTGTGGTGCACAACGCAACTGGCTTT

GTCATGGCGCGGAAACTTATTCATTTGGAAGTGAAGCAAGCCATCTTGAACCAAATCACACGAGAATTGC

AGGTTTTGCATGATTGTAGAAGCCCGTACATTGTTGGGTATTATGGGACTTTTTACAGTGATGGGGAAAT

CAGTATTTGTATGGAAAGCATGGACGCTGGGTCGCTTGATTTAGTGCTGAAGAAGGCGCGAAAAATTCCT

GAGATTTACTTGGGAAAAGTGAGCAAAGCTGTTATCCTTGGCCTCAAGTACTTAAGAGAAGAACGTAGCA

TCATTCATCGCGATGTAAAACCATCAAATATTCTCGTTAATTCCCGAGGAGAGATTAAGTTATGTGATTT

TGGCGTGAGCGGGCAACTGATCGACGATATGGCCAACGAGTTTGTAGGGACAAGATCATACATGGCCCCA

GAACGCTTGCAAGGATCCAAGTATACAATCCTTTCAGATATCTGGTCTCTTGGTCTCTCACTTATTGAAA

TGGCAATTGGTAGATTCCCTATCCCGCCACCCACAGCCAGCCAAATAGCAGCCATATTCAACACTGAAGT

GGCAGGGGGAAGTGGTAAAGCACCAAACCCACATGATGTTGCACGACCAATGGCAATCTTTGAGCTGCTT

GATTACATTGTGAATGAGCCAGCACCGAAACTCCCACAAGGAATTTTCGAGAAAGATTTTTGTGATTTCG

TGGCTAGTTGCTTGAAGAAAGAACCGAAAGAGCGATCAGATCTCGGGGAACTAATGAAGGCTCCATTTAT

TAAAAATGTTAGCTTAACCCAGTATGAGTTTGCTAAGTGGGTTTGCAGTACTATGGGTTTGAAAGCCCCG

AGTCCAGACACTGTACCTGATTAA

>Phox2 complete gene model(new exon 1, new exon 4-7)

ATGGACTACCCTGCGTATTTAGGGGTTGGCACAAATTATGACACGACTGCCTGTATGGTAGCTGCGGCAG

CTAATAACGATCCACACCAGCCTTACGTTACTACTCCATATGGGGACTTTAACTCATGCGCCCACGCCCA

GTCCTCTCAGAGCAACTTCGCGTCAGCTTATACACCGACAGTCCCGATCCGGAACTCCAGTTTTGGTGGC

GGAAACTGCCAGCCACCGATGCCTACAGCGGCCGCGTACGGCCTCAACAGCTTAAGGGATCAGTCGCCAT

ATTCTTCAGTACCTTGTAAGTTCTTCACCGAGACGGCCCATCAACACACCGGGGGCTACGGAGGACTCCA

CGAAAGGAGGAAGCAACGTCGCATCCGGACTACGTTCACAAGCTCCCAGTTAAAAGAGCTTGAAAAAGTT

TTCGCCGAAACTCATTACCCGGATATTTATACAAGAGAAGAACTTGCGCTAAAAATTGATCTCACTGAAG

CCAGAGTGCAGGTTTGGTTTCAAAATCGTCGAGCAAAATGGCGAAAAATGGAGCGAGCAAAACAACAACC

TCAACCCATACATTCTCCTGGGAGTTCCCCTTCTTCTCCCAACAATATCTCGTCCATAAACAACTCGGAA

AATATGATAAGTTCCGCAAGTCCTATGGAAGATATAGCTATTAGCTCAGTAAGCAGTGGAGCAATGATGG

AAGAAACAAACCAAGATCATCATTCAAGTCAGGATGACGTCATGGCTCTTAAAAACTCACTGCTAGGTAG

CGCTGCAGAGCTCGCAAGCATAGTCGGTAGTAAAAAACAAATTAGCACTCCTGTTCGAAACGAAAACATA

ACCGAAAATTCCGGTTTGCTATCTGATGACGTCACCACGCATCAATCCCATCATAATACGTCAGAAATGA

CGTCACCGCCCGGTTTTCGCGGTAAACCAATCCAATCTTTGATGTTACCAAACCATGGACAATCAGGATC

AAGTTATAACAGCAATCAACAGTTCATGCAAGGTCACAACCGATCTCAGCACAACACTTCATCATCGGTG

TCTTGGTTCCTATCCGGCGGGGGTAACAACGTCGCCACCCCGCTAAACACGACTCCTTTCGCCGATGTCC

TATCCGTTCTAACGCGTAACACACCCGGGTGCTCCACCGTCGCCGCTGCGGCAGTTTTGATGACGTCATC

ATCCCCCGTGACGTCAACTACTACGTCATACCAAAGCAGCGTAATGAGAATGAATCCACTGCCGGGGCCT

Development • Supplementary information

AATGGTGGATTCGACAAATCGTTCACCAACGGAGTCGGGAACAAACAGAGCAGAAATATGTTTCTGGATC
AGAGTCCAATTAACTCTGGCTCTTGCTCTGCGACAATTAACCACAGCCATAAGTTAAACTGA

>Unc76::StayGold (based on sequence from (Hirano, Ando et al. 2022))

ATGGCGGATCTGCGAGTACCGGACATTCCGCTCGCCTCGTGTGATGATGATGATATCGATAGTAATAAGA
ATTTGAGCAACCATTCATCAGACGAGAAACATCACTGCAACAGCAACAGCGACGAGGAACGTCTTCATGA
CGAGTTCTCTGGATCCCTTGAGGACCTTGTCGGCAACTTTGACGAAAAAATTGCGGCATGCCTGAAGGAC
CACGAGGTGACGACAGCGGATATTGCACCTGTGCAGATACGTACTCAAGAGGAAGTTATGAATGAAAGCC
AAACATGGTGGACATTAACCGGAAACTTTGGAAACATTCAACCTCTCGACTTTGGAACCTCTTCGATATG
TAAAAAGATGGCCGCAGCTCTGGACAGTGATTCATTGAAAGACGACGCATCTACACGCCGAAGTATGACA
AATTCCGATGATGAGGATCTTTTACGACAACAAATGGATGTTCATCAAATGATTGGACATCATCATGGAT
CTACGGATACTGGTGGTGAAACACCTCCACAGACTGCTGATCAAGTTATCGAAGAAATTGATGAAATGTT
ACAGGTACCGGTCGCCACCACTAGTGCCAGCACCCCCTTCAAGTTCCAGCTGAAGGGCACCATCAACGGC
AAGAGCTTCACCGTGGAAGGCGAGGGCGAGGGCAATAGCCACGAGGGCAGCCACAAAGGCAAGTACGTGT
GCACCAGCGGCAAACTGCCAATGTCTTGGGCCGCCCTGGGAACTAGCTTCGGCTATGGCATGAAGTACTA
CACCAAGTACCCCAGCGGCCTGAAGAACTGGTTCCACGAGGTGATGCCCGAGGGCTTCACCTACGACAGA
CACATCCAGTACAAGGGCGACGGCAGCATCCACGCCAAGCACCAGCACTTCATGAAGAACGGCACCTACC
ACAACATCGTGGAGTTCACCGGCCAGGACTTCAAGGAGAACAGCCCCGTGCTGACCGGCGACATGAACGT
GAGCCTGCCCAACGAGGTGCAGCACATCCCCAGAGATGACGGCGTGGAGTGCCCAGTGACCCTGCTGTAC
CCTCTGTTGAGCGACAAGAGCAAGTGCGTGGAGGCCCACCAGAACACCATCTGCAAGCCCCTGCACAATC
AGCCAGCCCCCGATGTGCCATACCACTGGATCAGAAAGCAGTACACCCAGAGCAAGGACGACACCGAGGA
GAGAGACCACATCTGCCAGAGCGAGACCCTGGAGGCCCACCTGTAA

>Unc76:mScarlet3 (based on a sequence from (Gadella Jr, van Weeren et al. 2023)

ATGGCGGATCTGCGAGTACCGGACATTCCGCTCGCCTCGTGTGATGATGATGATATCGATAGTAATAAGA
ATTTGAGCAACCATTCATCAGACGAGAAACATCACTGCAACAGCAACAGCGACGAGGAACGTCTTCATGA

Development • Supplementary information

CGAGTTCTCTGGATCCCTTGAGGACCTTGTCGGCAACTTTGACGAAAAAATTGCGGCATGCCTGAAGGAC

CACGAGGTGACGACAGCGGATATTGCACCTGTGCAGATACGTACTCAAGAGGAAGTTATGAATGAAAGCC

AAACATGGTGGACATTAACCGGAAACTTTGGAAACATTCAACCTCTCGACTTTGGAACCTCTTCGATATG

TAAAAAGATGGCCGCAGCTCTGGACAGTGATTCATTGAAAGACGACGCATCTACACGCCGAAGTATGACA

AATTCCGATGATGAGGATCTTTTACGACAACAAATGGATGTTCATCAAATGATTGGACATCATCATGGAT

CTACGGATACTGGTGGTGAAACACCTCCACAGACTGCTGATCAAGTTATCGAAGAAATTGATGAAATGTT

ACAGGTACCGGTCGCCACCGCTAGTATGGATAGCACCGAGGCAGTGATCAAGGAGTTCATGCGGTTCAAG

GTGCACATGGAGGGCTCCATGAACGGCCACGAGTTCGAGATCGAGGGCGAGGGCGAGGGCCGCCCCTACG

AGGGCACCCAGACCGCCAAGCTGAGGGTGACCAAGGGTGGCCCCCTGCCCTTCTCCTGGGACATCCTGTC

CCCTCAGTTCATGTACGGCTCCAGGGCCTTCACGAAGCACCCCGCCGACATCCCCGACTACTGGAAGCAG

TCCTTCCCCGAGGGCTTCAAGTGGGAGCGCGTGATGAACTTCGAGGACGGCGGCGCCGTGTCCGTGGCCC

AGGACACCTCCCTGGAGGACGGCACCCTGATCTACAAGGTGAAGCTCCGCGGCACCAACTTCCCTCCTGA

CGGCCCCGTAATGCAGAAGAAGACAATGGGCTGGGAAGCATCCACCGAGCGGTTGTACCCCGAGGACGTC

GTGCTGAAGGGCGACATTAAGATGGCCCTGCGCCTGAAGGACGGCGGTCGCTACCTGGCGGACTTCAAGA

CCACCTACAGGGCCAAGAAGCCCGTGCAGATGCCCGGCGCCTTCAACATCGACCGCAAGTTGGACATCAC

ATCCCACAACGAGGACTACACCGTGGTGGAACAGTACGAACGCTCCGTGGCCCGCCACTCCACCGGCGGC

TCCGGTGGCTCCTAA

>Kaede::NLS (Ando, Hama et al. 2002, Razy-Krajka, Lam et al. 2014)

ATGAGTCTGATTAAACCAGAAATGAAGATCAAGCTGCTTATGGAAGGCAATGTAAACGGGCACCAGTTTG

TTATCGAGGGAGATGGAAAAGGCCATCCTTTTGAGGGAAAACAGAGTATGGACCTTGTAGTCAAAGAAGG

CGCACCTCTCCCTTTTGCCTACGATATCTTGACAACAGCATTCCATTATGGTAACAGGGTTTTTGCTAAA

TACCCAGACCATATACCAGACTACTTCAAGCAGTCGTTTCCCAAAGGGTTTTCTTGGGAGCGAAGCCTGA

TGTTCGAGGACGGGGGCGTTTGCATCGCTACAAATGACATAACACTGAAAGGAGACACTTTTTTTAACAA

AGTTCGATTTGATGGCGTAAACTTTCCCCCAAATGGTCCTGTTATGCAGAAGAAGACTCTGAAATGGGAG

Development • Supplementary information

GCATCCACTGAGAAAATGTATTTGCGTGATGGAGTGTTGACGGGCGATATTACAATGGCTCTGCTGCTTA

AAGGAGATGTCCATTACCGATGTGACTTCAGAACTACTTACAAATCTAGGCAGGAGGGTGTCAAGTTGCC

AGGATATCACTTTGTCGATCACTGCATCAGCATATTGAGGCATGACAAAGACTACAACGAGGTTAAGCTG

TATGAGCACGCTGTTGCCCATTCTGGATTGCCGGACAACGTCAAGGGTTCTCCAAAAAAGAAAAGAAAAG

TTGACTAA

In situ probe templates:

>Vanabin4 (coding sequence)

ATGAAAACGTTCTGTGTTGTTACAATTGTACTTGTGCTTGCATCGGTGTGTGTTGATGCCCGTGGAAACC

GCCATCATGGAGGATTGATGGGAACTGGGGTGCCAAGGTGCCTGAAAACGTGCAAAGATGATTGCACGGA

AATGAAACCTTGCGCTTTAGCTACATGTCCTTCTGTCTGCCACGCAACTCGCGAAGCGGCCGAAGGCAGT

GGCGCTAATCGCTGCATGATCAGGTGCGGGTTGACTCAATGTTTGCCGAGATTCCCAAGCTGTAAAGCGT

GCGTCGCCCGTTGCGCTGCACCGGTTACCGCATGCAAGCGAAGCAGTTGTGCATCTGAGTGCCCAGCCGG

GATGACCATACCTGAGCACATGCAACTAAGTGGATGCGTTCGTTGCATGAAACGTAATTGCAGAAATATA

ATGAATGGGAACTAG

>Phox2 N-terminus gene model (KH.C14.100)

ATGCCTACAGCGGCaGCGTACGGCCTCAACAGCTTAAGGGATCAGTCGCCATATTCTTCAGTACCTTGTA

AGTTCTTCACCGAGACGGCCCATCAACACACCGGGGGATACGGAGGACTCCACGAAAGGAGGAAGCAACG

TCGCATCCGGACTACGTTCACAAGCTCCCAGTTAAAAGAGCTTGAAAAAGTTTTCGCCGAAACTCATTAC

CCGGATATTTATACAAGAGAAGAACTTGCGCTAAAAATTGATCTCACTGAAGCCAGAGTGCAGGTTTGGT

TTCAAAATCGTCGAGCAAAATGGCGAAAAATGGAGCGAGCAAAACAACAACCTCAACCCATACATTCTCC

TGGGAGTTCCCCTTCTTCTCCCAACAATATCTCGTCCATAAACAACTCGGAAAATATGATAAGTTCCGCA

AGTCCTATGGAAGATATAGGTAAGAGCAAGAGATGTATAGTT

>Phox2 C-terminus gene model (KH.C14.119)

GGTTTGCTATCTGATGACGTCACCACGCATCAATCCCATCATAATACGTCAGAAATGACGTCACCGCCCG

GTTTTCGCGGTAAACCAATCCAATCTTTGATGTTACCAAACCATGGACAATCAGGATCAAGTTATAACAG

Development • Supplementary information

CAATCAACAGTTCATGCAAGGTCACAACCGATCACAGCACAACACTTCATCATCGGTGTCTTGGTTCCTA

TCCGGTGGGGGTAACAACGTTGCCACCCCGCTAAATACGACTCCTTTCGCCGATGTCCTATCCGTTCTAA

CGCGTAACACACCCGGGTGCTCTACTGTCGCCGCTGCGGCAGTTTTGATGACGTCATCATCCCCCGTGAC

GTCAACTACTACGTCATACCAAAGCAGCGTAATGAGAATGAATCCACTGCCGGGGCCTAACGGTGGATTC

GACAAATCGTTCACCAACGGAGTCGGGAACAAACAGAGCAGAAATATGTTTCTGGATCAGcCCTATAGTG

AGTCGTATTA

>Pax2/5/8.a (coding sequence)

ATGAACTGGGGATCAGCAATGGCGGTTGGACCATCCAGTGTGGGACATCCATTCGTGGGATCAGGAATGG

CGGCCTCACTCACCCCTTCAAGATCAGGGCACGGTGGGGTGAACCAGTTGGGCGGGGTTTACGTGAACGG

CCGACCATTACCCGACCAAGTCCGACAACAAATAGTGGACCAAGCACACATAGGGGTTCGACCTTGTGAC

ATTGCTAGACAACTCCGGGTGTCGCATGGTTGTGTAAGCAAGATATTAGCAAGATATTACGAGACAGGCA

GCATCCGACCTGGTGTTATCGGTGGAAGCAAACCTAAGGTAGCTACACCTCGGGTAGTGGAGAAGATATG

TGATTATAAACGACAGAATCCAACTATGTTTGCTTGGGAGATACGAGACCGATTGTTGAGTGAGGGAATT

TGTGACCATGATAATGTACCCAGTGTTAGTTCGATCAATAGGATTGTCCGAAACAAAGCTGCAGAAAATG

CAAAGTCGCACCAACAACTCATGGTCCCAATGACGCCGTCATCGCTGGGTATCCACACAAACGGTCCGAT

ATTAATGGAACACGAGATTCGCGGAACGTACACGATTAACGATATATTAAGGTTACCCCACAACCCCCTT

CCCCCACATAACCCCCCCACCACTGAAACAGACCCAACACATTATAAACAAGCCGAAAATGGGATCCATT

ATAATCATAACGACAACTACAGCGAGCACATCGGGCAGGTGTCGCGCATGAACGGTTTCAATAGATTCGC

TTCATCGACCACAGCATTACCCACAATGCAACAATCAAGTCATGTGACTTCTCAGATGAATTTCGTTCGT

AAAGAAAACAAGGGATTGGATTATTCGTACGATTGTCGCGGTTCAACTAATTCACCCAACGTAGCTACTT

ACCCAGTGTTACCTCACAATCAACCTCGAGCTAACTGTGATGTCACAATCAGCCCAATGACATCACAAAC

CAACACTGCAAACGCGACAGTTTCACCCAGCAACAGTGGGGGTTACTCTGGGTCAAGTTTTGCCCCGATC

ACAACCGCGTACGCTCCGACAGGTGAGTTTGTGGATTATGGATATCAACAATACAATCAACACTGGAAGT

TTGGCCAACAGCACCATAATGATAGCAACACTGGTAAAGTTTTGAATCTTCGAGGTAAAGAACATCCGGC

AACACTGGAGATGGTCAGCGCTCAATAA

Development • Supplementary information

>Crls1 (coding sequence)

ATGATCAAATGCAGTTTGTCCATTCCAAGATTCCTGCAATCTAAAACTTTGAACTCAATCAAGACATTGG

AACGATGCCAACATGTAAAAAGTAATTGGGTGAAACACAGTGTTGGATGGTGCAACAATGGACATATTAA

CATAAAAACCTACAACACACTGGGACTTGGTCATCCTACTCTAAACAAGTTTCATCGTTGCCATTTAAGT

TGTATATCATCTCAGCAATGCAGATCATTCTCACTTAGTTGTAAGCTTCTATCTTCAAATGAAGAATGGG

AAAACCTGTTGGAAAATAAAAGCAAAAAGGAAAAAATTGAAAGTATTCCAGTGAAGAAAGAAAACATCTA

CACTGTCCCAAACCTACTTTCATTTTGTAGAATCGTGGTTTCCCCTTATCTCAGTTACCTTGTGCTTACT

GGTCAATCCCAAACAGCTTTAGTGTTGTGTGTGATTGCTGCCGTAACTGACATGATTGATGGACAAATAG

CTCGTACTTGGCCGTCACAACAAACAGCATTGGGTTCGGCACTTGATCCACTTGCTGATAAAGTTCTGGT

TGCTTTTCTTTCCCTTTCTCTCACATATGTCAACATTATACCATGGGCACTTACTGGTTTGTTCATTGGT

CGTGACGTCATCCTTATTCTCGCTGTGTTTTATCTACGTTACAAGACATGCCCACCCCCTGTAACTTGGG

AAAGATATTTTGATCCAAGTTTAGTTAATGTTAAACTCTCCCCAACCAACCTTAGCAAGGCAAACACTGC

GGCACAGTTGATTCTGTTATGGTGCTCTGTGGCTGCTCCTGTGTTTGGGTTTGTAGACCATGCTGCACTT

CGTTGTTTGTGGGCGTTCACTGCTTTTACTACAGTAGCTTCTGGTGTCAGTTATATCTTACAAAAAGACA

CAATTCGAATCATGAAAGAAAGCCCATGA

>FGF9/16/20 (coding sequence)

ATGTCTATGTTAACCAACATGTTAGGCCTCAGCAGCAATGTTCCGCAATCAGCAACGGGAACAAACTTTT

TAAGTTCGCTTTTACTCGCAGCCGTAGCCCGAGCGAGAGGCAAGGCAGTTACCGGACAATCGCCACCCGA

AAGCCCGTCGTCCGTCCTAGAAAGCTCGATGTCAAGAACCGAAGCAGCAAAAGTACAATCAGTTTTTTCA

GCCAAGCTTGGACGGGCAAGAAGAAAAAATTCCCTCTCTGTCGACTCCTCCACCCCCCAGAAATTAACAG

AAGAAACAATGCCTGTGGTACACGACCGACTCCCCGATCTCAGCCTTTGGAGCAAAATTGACGAGGAATT

AAAAGAGCAAGAGAAATCTTCCGCCAATGGACTATTGTCCTCAACCTCAGCAAGGAGTAAGAGAAACACC

GGTATGTCTGCCTACGACTACGATACCCAAGAAACTACCATGAGACGAAGAATGTTGTATTGTAAGAACG

GATTCAACCTTCAAATTTTACGAAACGGAAGAATATCTGGTACCCAAGAAAGCCACAATCAATACGCCGT

CCTTGAATTTATTTCTACTGGAATCGGAAGTCTCACCATCCGGGGTGTCGCAAGTGGTCTCTACCTGGCA

ATGAACAGCAAAGGAAGATTGTATGCTTCGGAAACATTCAATCGCGAGTGCATCTTCTACGAAACAGTAT

Development • Supplementary information

TGGAAAACAACCAGAACACGTTCGAATCATTCGCCCACAGAAATGGCTCAAAGAAATGCTACATCGCCCT

TGGTCGTCATGGAAGACCAAAGCAAGGATGCAGACTACACCCAAACAACCGACACAGCCAATTCTTGCCC

AGGAACATCGATACTGATAAAGTAAAAAACCTGTACTCTGGCCGACTCTACTGA

>EphrinA.b (coding sequence from Stolfi et al. 2011(Stolfi, Wagner et al. 2011))

ATGCCTTTGTCAAAAGTTAATCGAATGATTTTAAGTTTGTTTATGTGCTATCTTCTCTTTGATGGGGTAC

GAAGCAAGCGGTTTAATTCGGGGACCATCACCCATGAGGTTATGTGGGATCCACGAGAAAACGGAGGGTT

TGCTTTCGGAAACGAGTTCAGCATTGAGGTTCATATGCGAGACTACATGAATATTCACTGCCCTCAGTAT

GATCAAGATGATAAAGCTAAGCTTAGTTTCATCATCTACAATGTCAGCGAACAATCATACAACAGTTGCT

CTCTCACTGATATGAAAACTGCGATTTTTAAGTGTGACAACCCAGTTAAAGGACGCAAGCTCACCACCAA

GTTTCAACGAAGAAGTCCAAACCCACTGGGATTCGTATATCAACCAAATAAAGATTATTTCTTTATGGCA

TTCAAAAAAGATGAACCTCAGAATTGCAAATCAGCGATGAAAATGAAAGTTCATGTTCTGCCGAAAAGAC

TGCACGAACACGACCGGAAACCAGTCACTGCAGGTACAACACGGTCGTCAACGCCAAGGACACTTTCGTC

AACCACTACTACTACAACAATAGCAACAACAACAACAACAACACCCCGAACAAGCCGGAAACCCCATATA

CCAAGACAGAGCTCGACAAGAAATACTCCGCCTACAAAGACGTTTAAACCTGGATATGTCCGGGGCGACG

GTGAAGGTGACGGCCCGGGTGGCGGGGTCGGTAGAATTACTTGCGCCACCACGTGGATGTTAGTTGCGTT

AGTGCTGACTGTTCTGTTACAAAACTGA

Nori Satoh gene collection clones used for other in situ probes:

Eph.c: R1CiGC16p22

EphrinA.d: R1CiGC01j20

Development • Supplementary information

sgRNAS used in this study:

Pax2/5/8.a.3.75

gGTAGCTACACCTCGGGTAG (G+N19)

Pax2/5/8.a.3.54

gATCGGTGGAAGCAAACCTA (G+N19)

Phox2.3.43

gACGGCCCATCAACACACCG (G+N19)

Phox2.3.69

gCGGAGGACTCCACGAAAGG (G+N19)

Gli.2.11

gGTGGTAGTTCAGACTCAGG (G+N19)

Gli.2.81

gTTACGTCATCAGTCGACCC (G+N19)

Control (from Stolfi et al. 2014(Stolfi, Gandhi et al. 2014))

gCTTTGCTACGATCTACATT (G+N19)

Electroporation mixes for perturbation experiments

(per 700 µL of total cuvette volume)

Pax2/5/8.a driving GFP to look at Neck Cell Lineage

80 µg Pax2/5/8.a [-1961 to -1]>GFP

Pax2/5/8.a driving H2B:mCh & CiPhox2 Driving GFP to look at Neck Cell Lineage

20 µg Pax2/5/8.a [-1961 to -1]>H2B::mCh

80 µg CiPhox2 [-2951 to -1]>GFP

Pax2/5/8.a driving mCh & CiPhox2 Driving GFP to look at Neck Cell Lineage

80 µg Pax2/5/8.a [-1961 to -1]>mCh

80 µg CiPhox2 [-2951 to -1]>GFP

CrPhox2 Driving GFP to look at juvenile motor neurons after CRISPR

80 µg U6>F+EsgRNA Control or

40 µg U6>Pax2/5/8.a.2.54

40 µg U6>Pax2/5/8.a.2.75 or

40 µg U6>Phox2.3.43

Development • Supplementary information

40 µg U6>Phox2.3.69

40 µg Sox1/2/3>Cas9:Geminin

80 µg CrPhox2 [-2416 to +15]>GFP

CrPhox2 Driving GFP to look at cell fates after Pax2/5/8.a (tv1) overexpression

80 µg CrPhox2 [-2416 to +15]>GFP

80 µg Pax2/5/8.a [-1961 to -1]>mCh

80 µg Nut [-1155 to -1]>LacZ or

80 µg Nut [-1155 to -1]>Pax2/5/8.a (tv1)

Pax2/5/8.a variant overexpression for bulk RNA sequencing.

80 µg Nut [-1155 to -1]>LacZ or

80 µg Nut [-1155 to -1]>Pax2/5/8.a (tv1)

80 µg Nut [-1155 to -1]>Pax2/5/8.a (tv2)

Vanabin4 gene target validation and CRISPR

80 µg U6>Control F+E sgRNA or

40 µg U6>Pax2/5/8.a.2.54

40 µg U6>Pax2/5/8.a.2.75 or

Development • Supplementary information

80 µg Nut [-1155 to -1]>Pax2/5/8.a (tv1) or

80 µg Nut [-1155 to -1]>LacZ or

40 µg Sox1/2/3>Cas9:Geminin

20 µg Nut [-1155 to -1]>H2B:mCh

Gli expression after Pax2/5/8.a CRISPR

80 µg U6>Control F+E sgRNA or

40 µg U6>Pax2/5/8.a.2.54

40 µg U6>Pax2/5/8.a.2.75 or

40 µg Sox1/2/3>Cas9:Geminin

20 µg Sox1/2/3>H2B:mCh

80 µg Gli [-3339 to +57]>GFP

Neck specific expression of FGF and Ephrin signaling proteins

80 µg Pax2/5/8.a [-1961 to -1]>LacZ or

80 µg Pax2/5/8.a [-1961 to -1]>dnFGR or

80 µg Pax2/5/8.a [-1961 to -1]>dnEph.c or

80 µg Pax2/5/8.a [-1961 to -1]>caMEK

80 µg CrPhox2 [-2416 to +15]>GFP

20 µg Pax2/5/8.a [-1961 to -1]>H2B::mCh

80 µg Pax2/5/8.a [-1961 to -1]>mCh

Neck Specific Expression of BMP receptors

80 µg Pax2/5/8.a [-1961 to -1]>LacZ or

80 µg Pax2/5/8.a [-1961 to -1]>caBMPR or

80 µg Pax2/5/8.a [-1961 to -1]>dnBMPR

20 µg Pax2/5/8.a [-1961 to -1]>H2B::mCh

80 µg Pax2/5/8.a [-1961 to -1]>GFP

Neck morphology after Gli CRISPR

80 µg U6>Control F+E sgRNA or

40 µg U6>Gli.2.11

40 µg U6>Gli.2.81

40 µg Sox1/2/3>Cas9:Geminin

80 µg Sox1/2/3>GFP

80 µg Pax2/5/8.a [-1961 to -1]>mCh

Precocious CMNs in juvenile after Neck-specific manipulations of FGF and Ephrin signaling

80 µg Pax2/5/8.a [-1961 to -1]>LacZ or

80 µg Pax2/5/8.a [-1961 to -1]>dnFGR or

80 µg Pax2/5/8.a [-1961 to -1]>dnEph.c or

80 µg Pax2/5/8.a [-1961 to -1]>caMEK

20 µg Pax2/5/8.a [-1961 to -1]>H2B::mCh

80 µg Pax2/5/8.a [-1961 to -1]>GFP

Neck CMN and NN visualization with GRIK reporter

80 µg Pax2/5/8.a [-1961 to -1]>mSc3 or

Development • Supplementary information

80 µg CrPhox2 [-2416 to +15]>mSc3

80 µg GRIK [intron 1 +bpFOG]>GFP

Visualization of dpERK

80 µg Pax2/5/8.a [-1961 to -1]>LacZ or

80 µg Pax2/5/8.a [-1961 to -1]>dnFGR or

20 µg Pax2/5/8.a [-1961 to -1]>H2B::mCh

GRIK expression after Pax2/5/8.a CRISPR

80 µg U6>Control F+E sgRNA or

40 µg U6>Pax2/5/8.a.2.54 with

40 µg U6>Pax2/5/8.a.2.75

40 µg Sox1/2/3>Cas9:Geminin

20 µg Sox1/2/3>H2B::mCh

80 µg GRIK [intron 1 +bpFOG]>GFP

Development • Supplementary information

Supplemental References

Ando, R., H. Hama, M. Yamamoto-Hino, H. Mizuno and A. Miyawaki (2002). "An optical marker based on the UV-induced green-to-red photoconversion of a fluorescent protein." Proceedings of the National Academy of Sciences **99**(20): 12651-12656.
Brozovic, M., C. Dantec, J. Dardaillon, D. Dauga, E. Faure, M. Gineste, A. Louis, M. Naville, K. R. Nitta and J. Piette (2018). "ANISEED 2017: extending the integrated ascidian database to the exploration and evolutionary comparison of genome-scale datasets." Nucleic acids research **46**(D1): D718-D725.
Cao, C., L. A. Lemaire, W. Wang, P. H. Yoon, Y. A. Choi, L. R. Parsons, J. C. Matese, W. Wang, M. Levine and K. Chen (2019). "Comprehensive single-cell transcriptome lineages of a proto-vertebrate." Nature **571**(7765): 349-354.
Davidson, B., W. Shi, J. Beh, L. Christiaen and M. Levine (2006). "FGF signaling delineates the cardiac progenitor field in the simple chordate, Ciona intestinalis." Genes & development **20**(19): 2728-2738.
Dufour, H. D., Z. Chettouh, C. Deyts, R. De Rosa, C. Goridis, J.-S. Joly and J.-F. Brunet (2006). "Precraniate origin of cranial motoneurons." Proceedings of the National Academy of Sciences **103**(23): 8727-8732.
Gadella Jr, T. W., L. van Weeren, J. Stouthamer, M. A. Hink, A. H. Wolters, B. N. Giepmans, S. Aumonier, J. Dupuy and A. Royant (2023). "mScarlet3: a brilliant and fast-maturing red fluorescent protein." Nature Methods **20**(4): 541-545.
Hirano, M., R. Ando, S. Shimozono, M. Sugiyama, N. Takeda, H. Kurokawa, R. Deguchi, K. Endo, K. Haga and R. Takai-Todaka (2022). "A highly photostable and bright green fluorescent protein." Nature Biotechnology **40**(7): 1132-1142.
Oonuma, K., M. Yamamoto, N. Moritsugu, N. Okawa, M. Mukai, M. Sotani, S. Tsunemi, H. Sugimoto, E. Nakagome and Y. Hasegawa (2021). "Evolution of Developmental Programs for the Midline Structures in Chordates: Insights From Gene Regulation in the Floor Plate and Hypochord Homologues of Ciona Embryos." Frontiers in Cell and Developmental Biology **9**: 704367.
Picco, V., C. Hudson and H. Yasuo (2007). "Ephrin-Eph signalling drives the asymmetric division of notochord/neural precursors in Ciona embryos."
Razy-Krajka, F., B. Gravez, N. Kaplan, C. Racioppi, W. Wang and L. Christiaen (2018). "An FGF-driven feed-forward circuit patterns the cardiopharyngeal mesoderm in space and time." Elife **7**: e29656. Razy-Krajka, F., K. Lam, W. Wang, A. Stolfi, M. Joly, R. Bonneau and L. Christiaen (2014). "Collier/OLF/EBF-dependent transcriptional dynamics control pharyngeal muscle specification from primed cardiopharyngeal progenitors." Developmental cell **29**(3): 263-276.
Rothbächer, U., V. Bertrand, C. Lamy and P. Lemaire (2007). "A combinatorial code of maternal GATA, Ets and β-catenin-TCF transcription factors specifies and patterns the early ascidian ectoderm."
Satou, Y., T. Kawashima, E. Shoguchi, A. Nakayama and N. Satoh (2005). "An integrated database of the ascidian, Ciona intestinalis: towards functional genomics." Zoological science **22**(8): 837-843.
Shimai, K., Y. Kitaura, Y. Tamari and T. Nishikata (2010). "Upstream regulatory sequences required for specific gene expression in the ascidian neural tube." Zoological science **27**(2): 76-83.
Song, M., X. Yuan, C. Racioppi, M. Leslie, N. Stutt, A. Aleksandrova, L. Christiaen, M. D. Wilson and I. C. Scott (2022). "GATA4/5/6 family transcription factors are conserved determinants of cardiac versus pharyngeal mesoderm fate." Science Advances **8**(10): eabg0834.
Stolfi, A., S. Gandhi, F. Salek and L. Christiaen (2014). "Tissue-specific genome editing in Ciona embryos by CRISPR/Cas9." Development **141**(21): 4115-4120.
Stolfi, A., E. K. Lowe, C. Racioppi, F. Ristoratore, C. T. Brown, B. J. Swalla and L. Christiaen (2014). "Divergent mechanisms regulate conserved cardiopharyngeal development and gene expression in distantly related ascidians." elife **3**: e03728.

Stolfi, A., E. Wagner, J. M. Taliaferro, S. Chou and M. Levine (2011). "Neural tube patterning by Ephrin, FGF and Notch signaling relays." Development **138**(24): 5429-5439.
