# Supplementary material for: Specification and survival of post-metamorphic branchiomeric neurons in a non-vertebrate chordate
Source: Development. 2024 Jul 17;151(20):dev202719. doi: 10.1242/dev.202719 (PMC11273300; doi:10.1242/dev.202719)
Supplement: Supplementary file 1 [file develop-151-202719_review_history.pdf]

Specification and survival of post-metamorphic branchiomeric neurons in a non-vertebrate chordate

Eduardo D. Gigante, Katarzyna M. Piekarz, Alexandra Gurgis, Leslie Cohen, Florian Razy-Krajka, Sydney Popsuj, Christopher J. Johnson, Hussan S. Ali, Shruthi Mohana Sundaram and Alberto Stolfi
DOI: 10.1242/dev.202719

Editor: Francois Guillemot

Review timeline
Original submission: 15 August 2023
Editorial decision: 22 September 2023
Resubmission: 22 January 2024
Editorial decision: 18 March 2024
First revision received: 4 June 2024
Accepted: 10 June 2024

Original submission

Original decision letter

MS ID#: DEVELOP/2023/202266

MS TITLE: Specification and survival of post-metamorphic branchiomeric neurons in the hindbrain of a non-vertebrate chordate.

AUTHORS: Eduardo D Gigante, Katarzyna M Piekarz, Alexandra Gurgis, Leslie Cohen, Florian Razy-Krajka, Sydney Popsuj, Hussan S Ali, Shruthi Mohana Sundaram, and Alberto Stolfi

I have now received the reports of three referees on your manuscript and I have reached a decision. The referees' comments are appended below, or you can access them online: please go to BenchPress and click on the 'Manuscripts with Decisions' queue in the Author Area.

As you will see, all the referees are enthusiastic about your work, but they also have significant criticisms and recommend a substantial revision of your manuscript before we can consider publication. If you are able to revise the manuscript along the lines suggested, which may involve further experiments, I will be happy receive a revised version of the manuscript. Your revised paper will be re-reviewed by one or more of the original referees, and acceptance of your manuscript will depend on your addressing satisfactorily the reviewers' major concerns. Please also note that Development will normally permit only one round of major revision. If it would be helpful, you are welcome to contact us to discuss your revision in greater detail. Please send us a point-by-point response indicating your plans for addressing the referees' comments, and we will look over this and provide further guidance.

Please attend to all of the reviewers' comments and ensure that you clearly highlight all changes made in the revised manuscript. Please avoid using 'Tracked changes' in Word files as these are lost in PDF conversion. I should be grateful if you would also provide a point-by-point response detailing how you have dealt with the points raised by the reviewers in the 'Response to Reviewers' box. If you do not agree with any of their criticisms or suggestions please explain clearly why this is so.

© 2024. Published by The Company of Biologists under the terms of the Creative Commons Attribution License (https://creativecommons.org/licenses/by/4.0/).

Reviewer 1

Advance summary and potential significance to field

In this study Gigante and colleagues analyse the GRN regulating the specification of a population of neurons in the 'neck' region of the sea squirt Ciona, a region presumed homologous to (roughly) the hindbrain of vertebrates.
There are two previously identified transcription factors that are expressed here, Pax2/5/8 and Phox2, both of which have had some work done on them previously. Reporter transgenes are used to generate a cell-level description of expression, then CRISPR and overexpression to dissect their function.
Overexpression is analysed using transcriptomics, and further insight is leveraged from re-analysis of published scRNAseq data. Finally, the role of FGF and Ephrin signalling is explored.
In my view there are three important potential outcomes of this work:

i. A robust analysis of TF functions in the specification of these cell types
ii. Analysis of the roles of FGF signalling in balancing differentiation with proliferation
iii. An indication that some of these larval neurons persist through metamorphosis, somewhat contrary to general expectations

The implication of a function for vanadium binding proteins in these neural cells is also a fascinating if preliminary funding. This deserves more work, but this is out of the scope of the current paper.

Comments for the author

In general the experiments are well described, appropriately controlled and the data convincing. Ciona is not that well understood by most biologists and the authors have done a good job of generating high quality, relatively easy-to-interpret figures. I also appreciate the leg work they have put into things like improving gene models, and the full traceability provided for this in the supplementary data. I have one broader point to make about (iii) above, and a couple of smaller things to add.
1. With respect to (iii) above, the only important gap in the paper is that lineage the connection between early cells (in the embryo and larva) and cells post metamorphosis is not fully demonstrated. I do agree that their maintenance is the most parsimonious explanation of what is seen, and that older data (Like Dufour et al 2006) support their interpretation, but as this is a non-canonical finding I expected something more solid. I do though note that the authors have in places been a bit more cautious in their wording about this. I think the manuscript would be improved if the authors either extended this approach a bit further (eg giving more discussion to the caveats and alternate explanations), or provided some more compelling evidence that we were dealing with the same cells in embryos and adults.
2. Pg 7 lines 148-149: perhaps I missed something here but isn't the phenotype a lack of axon? Extrapolating to specification and differentiation seems a bit of an overreach?
3. I appreciate the extensive supplementary data provided, but it could do with a contents page of similar to help navigation.

Reviewer 2

Advance summary and potential significance to field

In this paper, Gigante et al examined how the neck region in an ascidian larva is specified. They first traced neck cells during formation of larva and metamorphosis. Then, they examined Pax258 function and Phox2 function, because these two genes are expressed in the neck. Finally, they found that differential FGF signaling is required for differentiation and survival of neurons in the neck. It is an interesting topic how adult body parts are formed during metamorphosis in ascidians, because it may be related to elucidation of the origin of vertebrates and evolution of chordates. This paper also contributes to our understanding how neural cells are specified in chordate

© 2024. Published by The Company of Biologists under the terms of the Creative Commons Attribution License (https://creativecommons.org/licenses/by/4.0/).

embryos. Therefore, I would like to support publication of this manuscript basically, but several simple experiments and clarification are required.

Comments for the author

Major comments

1) Title: There is no clear consensus that the neck region of ascidian larvae corresponds to the hindbrain of vertebrates. In line 69, two papers are cited to call this region "hindbrain". Other hypotheses should be explained here. For example, one paper, in which the last author of the submitted paper is a co-author, indicate a similarity between the motor ganglion and vertebrate MHB. I, probably as well as many researchers, think that it is difficult to call a particular part of the ascidian CNS a hindbrain counterpart. I think that the authors need to change the title, and rewrite the introductory part more properly.

2) The authors may think that the ascidian development can be divided clearly into two phases; larval and adult lives. But this is not completely true. Some adult structures begin to differentiate before metamorphosis, although differentiation of adult neurons in Ciona larvae might be new. See DOI:10.2108/zsj.21.285 and DOI: 10.1016/j.semcdb.2007.04.005, for example. On the basis of such prior knowledge, I request the authors to rewrite Summary, Introduction, and Discussion.

3) The authors wrote that adult neurons begin to differentiate before metamorphosis (Line 130). This point is one of the most important results in this paper, but I have several concerns: (A) I can see an axon-like structure there, but I cannot see where this structure comes from. (B) How do the authors know that this "neuron" does not function in larvae? (C) How do the authors know that the adult neurons come from A13.242 (Figure 2M)? (D) Is there any evidence that the adult Phox2+/Pax+ cells are the same cells that express Phox2 and Pax in larvae?

4) FGF signaling: (line 233-271): Involvement of FGF signaling is interesting. However, there are no data indicating where FGF signaling acts. Immunostaining with an anti-dpERK antibody is necessary, as many Ciona laboratories do. If the authors' claim is right, dpERK signal will be found in undifferentiated cells only. In addition, to validate the experiments with mutant constructs, dpERK staining will be helpful.

Minor comments

5) "Neck" should be "neck".

6) Line 48: Please cite the amphioxus genome paper whenever Delsuc's paper is cited. Delsuc's paper ignores the general consensus of scientific communities (i.e. although they extensively used the data from the amphioxus genome project, they published their analysis before the genome paper).

7) Line 96: In Figure 2A, GFP, but not mCherry, is used. The text contradicts the figure.

8) Line 98: Ikuta 2007 (and possibly Imai 2009) should be cited here.

9) Line 100 & Figure 2C: GFP signals are vague. Replace the photograph.

10) Line 108: Italicize "Phox2"

11) Line 111 & Figure 2FG: There may be four Phox2+ cells in Figure 2F, but I can see only three cells strongly expressing Phox2 in Figure 2G. H2B::mCh may be too strong.

12) Line 115 & Figure 2G: Phox2 expression expands anteriorly. How many cells? H2B::mCh may be too weak in the expanded cells. DAPI may be helpful.

13) Line 115: The authors probably think that this anterior expansion is expression in A13.247/8. What is the rationale for this interpretation.

14) Line 106 & 117: The authors found variations among individuals. However, the experiments are all done with transient introduction of reporter genes. It is well known that not all cells express reporter genes probably because of mosaic incorporation. Therefore, the authors need to show that the observation truly represents variations or an artifact.

15) All lineage tracing experiments were done with reporter genes. As I wrote in the preceding comment, I strongly suggest the authors to confirm their conclusion with in situ hybridization. In situ hybridization of larvae is a bit difficult, but many laboratories report larval in situ hybridization. Knockout of the cellulose synthase gene may be helpful, as several laboratories did. I do not necessarily suggest to do this at all stage; a few critical stages will be enough.

16) Line 119: Does Imai et al really assume neck cells are quiescent?

17) Figure 2M: This figure suggests CMNs are derived from A13.242 and the neck neuron is from A13.241. Show the rationale for this inference. CMNs express Phox2. I cannot see any evidence for this expression. Is there any possibility that these cells are derived from A12.122/3? If not, why?

© 2024. Published by The Company of Biologists under the terms of the Creative Commons Attribution License (https://creativecommons.org/licenses/by/4.0/).

18) Figure 2I/J: Cell boundaries are not clear. How many cells? How did the authors identify each of CMNs and NN.
19) Figure 2K/L: I want to see a bright field view, because gills are not clear in these photographs.
20) Line 142: Explain the Sox promoter.
21) Figure 2 legend and others: Explain the methods to detect expression for each panel. Double in situ hybridization? In situ hybridization + immunostaining?
22) Supplementary figures do not appear in the numerical order.
23) In figure S1, CRISPR/CAS9 efficiency was examined. Details are necessary in the Method section. It is also a better idea to add one sentence to explain how the authors examined the efficiency in the main text around line 150.
24) While Phox2>GFP is observed in many cells other than the neural tissue in the control shown in Figure 3C, such expression is not seen in Pax2 knockout or Phox2 knockout as well as axons innervating gill slits. Explain why this happened.
25) Line 153: A reference for Nut is required.
26) Line 155 & Figure 3F: The authors say that overexpression of Pax2 did not change the number of gill slit innervating CMN axons. However, from Figure 3F, I have an impression that the number of axons is increased. In addition, there are several axons extending to the lower side. These axons are not counted probably because they do not innervate gill slits. To interpret this experiment precisely, I feel that the authors show more quantitative data about the number of axons (including axons not innervating gill slits) or neurons with Phox2 expression.
27) Line 171: "Motor Ganglion" should be "motor ganglion".
28) Figure 4EFG: Show pictures for the green channel only in addition to the current presentation.
29) Figure 5E: The scRNAseq logFC value for Phox2 is 2.03 in this figure, but it is 1.71 in Table S3. The bulk RNAseq logFC values for Phox2 are 0.15 in Figure 5E and 0.21 and 0.20 in Table S1. I found these discrepancies by chance. Check all values in this figure and the tables again.
30) Line 225: Subcluster 0 represents the neck region, and the authors found there are at least two cell populations in this subcluster, which probably correspond to Phox2+ and Phox2- cells in the neck region. If so, this subcluster will be further sub-divided with an appropriate parameter for clustering, and marker genes for these sub-sub-cluster will reveal differences in transcriptomes of these two populations within the neck region. Otherwise, this part looks to be too shallow.
31) Line 235-240: Involvement of FGF signaling had been examined mainly by Nishida's group in Halocynthia, and by Lemaire's group in Ciona. A FGF gene was first identified by Imai et al (2002) in Ciona. The antagonism between FGF and Ephrin signaling is first described in this animal by Picco et al (2007). It will be a better idea to cite these critical papers here. As the authors may know, there are many additional papers that should be cited.
32) Figure 6A-D: Show pictures for the green channel only in addition to the pictures shown in these panels. Depiction of the expression patterns will be helpful.
33) Line 272-279: Here the authors examined BMP signaling and Hh signaling. Because the authors did not provide evidence for action of CaBMPR and dnBMPR in the neck region, it is impossible to interpret the result shown in Figure S6A-D. On the other hand, in embryos with Gli knockout, anterior axons are not seen even in the control embryo. I am not sure whether no significant effect on neck morphogenesis was observed. This part is not relevant to the main conclusion. So I do not care if the authors remove this part.
34) Line 308: If this is a study using mouse embryos, these genes should be written Phox2 and Pax2/Pax5 (but not PHOX2 or PAX2/5)

Reviewer 3

Advance summary and potential significance to field

While most ascidian developmental biology studies focus on the stereotyped embryonic program, this study addresses an important but understudied question: the origin of the adult nervous system.

Previous work suggested that larval neurones degenerate during metamorphosis and are subsequently replaced with adult ones generated from a pool of undifferentiated larval neural progenitors. The main finding of this work is that adult motoneurones start differentiating during

© 2024. Published by The Company of Biologists under the terms of the Creative Commons Attribution License (https://creativecommons.org/licenses/by/4.0/).

the larval stages, much earlier than previously thought. The larval to adult neuronal transition is thus less abrupt than classically thought (although Horie 2011 already reported that some cholinergic adult neurones may be derived from larval neurones).

Building on this finding and on previous studies, the authors dissect the regulatory network and signalling pathways involved in the specification and the differentiation of these neurones. The identity of the involved transcription factors, Pax2/5/8 and Phox2, conserved midbrain/hindbrain regulators, strengthens the existence of a conserved branchiomeric motoneurone program originating in the common ascidian-vertebrate ancestor or earlier.

Comments for the author

The most important finding of this study is that adult neurones start differentiating during the larval stages. This part of the work is compelling and of high quality. I only have minor comments:
1) Figure 1D states that the sensory vesicle and visceral ganglion do not contribute to the adult nervous system. This seems at odds with Horie et al., 2011, which report that "we conclude that the anterior parts of the ascidian larval CNS, namely the sensory vesicle, posterior brain, neck and visceral ganglion, contribute to the formation of the adult CNS". It should be clearly stated on the figure, not just in the legend, that this statement only applies to larval neurones, not to the larval CNS territories themselves. Also Horie reports (Fig 3b) that some adult posterior cholinergic neurones are derived from larval cholinergic neurons in the visceral ganglion.
2) The authors trace lineages using reporter constructs driving classical fluorescent proteins, rather than by photoconversion as in Horie et al., 2011. How stable are the expressed FPs? Can the authors rule out that new expression domains of the reporters complicate the interpretation of the data?
3) The authors propose that Pax2/5/8+,Phx2+ larval neck derived neurones correspond to previously described cholinergic ciliomotor neurones (Fig 2L). Can the authors provide stronger evidence for this claim, for instance that these neurones are indeed cholinergic?
4) Dechorionation is known to affect the formation of the larval CNS as it perturbs left-right asymmetry. Could this have any effect on the interpretation of the experiments?
The second part of the manuscript describes the regulatory cascade that 1) specifies the neck and 2) controls the balance between adult neuronal differentiation and proliferation. This part is of heterogeneous quality. The CRISPR analysis of the role of Pax2/5/8 and Phox2 is convincing, as is the role of FGF in the control of the balance between proliferation and differentiation. By contrast, the bulk and single-cell RNA-seq analyses of the downstream program are too superficial to bring more to the study than a list of candidate genes for subsequent analysis. These analyses should be either expanded to extract a major message or, better, removed form this manuscript and saved for another functional study focusing on the events downstream of Pax and Phox. The section on the correction of the Phox2 gene model should be shifted to materials and methods.
Taken together, this is a high-quality study combining lineage tracing with functional gain and loss of function experiments to address an important question in the tunicate field. It will benefit from removing the non-necessary RNA-seq parts.

Response to Reviewers

We thank the reviewers and editor for their comments and the opportunity to revise the manuscript. See our replies to each comment below:

Reviewer 1 Comments for the author
The following numbers correspond to the numbers in my previous review.
1) Title: Even if the neck region shares the evolutionary origin with the vertebrate hindbrain, the ascidian neck region cannot be called as the hindbrain. The current title may be very catchy but is misleading and incorrect. A scientifically correct title is more important than a sensational title, I believe.
Following the reviewer’s suggestion we have changed our title to “Specification and survival of post-metamorphic branchiomeric neurons in a non-vertebrate chordate”.

3) Lineage: (A) To substantiate the authors’ claim that the CMNs are already differentiating in larvae, the authors need to show that these cells indeed have neurites. But I cannot see which cells extend the neurites even in the revised version. This point is central to this manuscript. In Figure

© 2024. Published by The Company of Biologists under the terms of the Creative Commons Attribution License (https://creativecommons.org/licenses/by/4.0/).

1H and I, it is not clear if the CMNs extend neurites. It is even possible that these come from NNs. This demonstration is the critical point of this manuscript but not sufficient.
Based on our experience imaging neurons in the Ciona larva using fluorescent reporters, we concluded that the axon projecting posteriorly towards the tail is the only Neck Neuron-derived neurite, while the others are coming from the more anterior delaminated cells, the putative CMNs. We have tried to present additional images that make this clearer, from a wider range of examples of imaged larvae using the contrast between nuclear H2B::mCherry and cytoplasmic Unc-76::GFP to better delineate cells and identify which neurites might be coming from which cells. These data have been included as Supplemental Figure 2.
(C) Contrary to the response letter, no legend for Figure 1L was found.
We apologize for the confusion; the relevant legend (2L) was present in the figure legend of our original submission but was lost in the final revised file that was re-submitted. It has been correctly replaced in the latest resubmission
(D) I had misunderstood the data in my previous review. I am wondering if the other two reviewers may have misunderstood the data similarly. But the problem how the authors traced the CMN lineage stands anyway. GRIK>GFP supports the authors' idea, but it is not conclusive. As Phox>GFP starts to be expressed in CMNs only after metamorphosis, the same may occur for GRIK>GFP. The authors say "clear continuity between GRIK>GFP expression" in their answer to Reviewer1, but it is not clear from Figure 1. Stage 30 in Figure 1I corresponds to 25-27 hpf and Stage 36 corresponds to 45-60 hpf. Namely, there is a big gap between these two stages. This "lineage" problem is another critical point of this manuscript. This should be examined much more carefully. Dufor 2006 assumed that larval Phox+ cells gave rise to adult Phox+ cells. But this manuscript denies this. More concrete evidence is necessary. The way the third reviewer pointed out will be the best option. Live imaging or time-lapse imaging will also resolve this problem.
To address this question, we first attempted to perform live imaging time-lapse experiments in metamorphosing individuals expressing the GRIK>GFP reporter between 22 and 44hpf. Due to the fact that these individuals were not fixed/cleared and thus were more optically opaque, and due to the complex movements of the animal, tissues, and cells during body rotation, we were unable to resolve and track the thin neurites through metamorphosis.
However, we were able to successfully carry out tracking via photoconversion of Kaede, as now shown in the new Figure 3. We photoconverted Kaede driven by the GRIK promoter, which is only active in the delaminated, Phox2-negative cells in the Neck during larval stage. We let these individuals undergo metamorphosis, in parallel with individuals electroporated with the same GRIK>Kaede reporter but NOT subjected to photoconversion. In the non-photoconverted juveniles, we see green Kaede signal but no red signal, indicating the successful lack of photoconversion in the control. In contrast, we see red Kaede signal in the juvenile CNS of individuals who were subjected to photoconversion. These results are consistent with the survival of GRIK+ larval cells through metamorphosis. Unfortunately, we were not able to make yet a functional Unc-76-tagged Kaede that can fill the axons effectively. Kaede forms tetramers, which limits its use in protein fusions. Future work will need to address the problem of tracking the neurites/axons through metamorphosis.
We have now updated the text to incorporate our Kaede photoconversion results, and discuss its implications. We suggest that it is possible that there may be some variability in which larval cells (Phox2+ or GRIK+) give rise to which CMNs. As the CMNs will undoubtedly multiply as the individual grows and additional gill slits, or stigmata, appear, there seems to be the need for a pool of undifferentiated CMN progenitors through later stages of post-metamorphic growth, which could be fulfilled by the Phox2+ cells. It may be that GRIK+ cells seen in the larva are indeed the earliest differentiating CMNs, but that their survival and further differentiation might be dictated by the timing and speed of metamorphosis. It is possible that in some cases in which settlement or feeding might be delayed (like during overwintering), early differentiating CMNs could plausibly regress/senesce, to be replaced later by later-differentiating cells. Eventually, all CMNs appear to express both Phox2 and GRIK reporters, making this technically quite challenging to study given our currently available approaches.

4) In Figure 6E, the authors claims that the anterior three cells are dpERK+ and the posterior one (fourth cell) is dpERK-. But the second cell looks to be dpERK- too. The second one may be in a different focal plane, but if so, show all focal planes necessary for substantiating the authors' claim.
We have now provided more quantitative analysis of the dpERK staining across the neck in both wild-type and dnFGFR conditions, and we see some differences in the 2nd cell relative to the 1st

© 2024. Published by The Company of Biologists under the terms of the Creative Commons Attribution License (https://creativecommons.org/licenses/by/4.0/).

and 3rd cell (hinting at possible mechanisms of further heterogeneity seen in the anterior neck), but staining is still stronger than in the 4th cell. And of course all staining is uniformly negative in the dnFGFR condition. These data have been included as Supplemental Figure 7.
Minor Comments
5) line 292: “neck Neuron” should be “neck neuron”.
All instances have been corrected.

9) If GFP signals are too weak and the authors cannot present evidence, the authors cannot say anything from this picture. Cells are not identifiable in Figure S3, either. Changing the sentence (line 109-110) is necessary. If the authors cite earlier studies and change wording, this part will be persuasive even without updating the data.
We have provided an updated figure with clearer GFP expression. We repeated the experiment using a mouse anti-GFP antibody to detect transient, low-level GFP expression that might be too low or early to detect via direct fluorescence (given the maturation time of GFP). With GFP antibody staining, we observed all four Pax2/5/8+ cells were GFP+ during the mid-tailbud stage. We note that several hours later during the larval phase, Phox2>GFP is no longer discernible to in the four posterior, delaminated Pax2/5/8+ cells, in contrast to the bright expression in the anterior cells. We conclude that the early expression of Phox2 at the mid-tailbud stage is likely transient, low-level expression, which has been observed for other genes and/or reporters in Ciona (e.g. MRF in the B7.5 lineage as described in Tolkin and Christiaen 2016). We conclude that after the mid-tailbud stage, Phox2 is downregulated in the posterior, delaminated cells and GFP expressed by the Phox2 promoter in our reporter plasmid early is not high enough or does not persist long enough for those posterior cells to inherit GFP at easily detected levels. This suggests that the high-level expression of Phox2>GFP in the ependymal neck cells of the larva is due to sustained/upregulated expression. Together, we hypothesize that Phox2 expression in the Neck throughout embryogenesis and metamorphosis is not constant and is highly dynamic. Further studies on the temporal dynamics of Phox2 regulation and function are ongoing our lab. We have updated the figure and text to accommodate these new data.

13) I was not able to find the “alternative interpretation”, which is written in the response letter, in the revised version.
We apologize for not making these alternative interpretations more explicit, we have now clearly stated our alternative interpretations in the manuscript (highlighted).

17) Now GRIK>GFP supports the idea that Phox2+ cells in adults are derived from A12.121, even though it is not conclusive. The explanation written in the response letter should be written in the main text of the manuscript.
Actually, in adults, GRIK and Phox2 reporters are co-expressed, suggesting that even though in the larva the expression domains of GRIK and Phox2 are mutually exclusive, they are merge at some point. This might be tied to possible variability in CMN specification. We have expanded on this point in the discussion.

23) I cannot figure out what percentages of genes in embryos were knocked out from Figure S7. Show them by numbers, but not by graphs only. Are these proportions consistent with proportions of embryos with phenotypes? These should be included in the manuscript.
We have updated the figure (we assume the old Figure S8, now Figure S10) with the numerical efficacy values given by the sequencing service company (Azenta). However, for Phox2 this number is a slight overestimation due to the low frequency (~2.5%) of a small naturally occurring indel in the analyzed region.
The overall efficacies of the sgRNAs are validated by using a ubiquitously-expressing promoter (Eef1a) to drive expression of Cas9. The actual efficacy in the specific lineage analyzed (e.g. the neck) will vary from the overall efficacy based on the strength/timing of the lineage-specific driver used to drive Cas9 for phenotyping experiments, the number of sgRNAs used, the relative amounts of plasmid electroporated. The actual relationship with phenotype will further vary according to haploinsufficiency, the likelihood that a frameshift will occur upon indel repair, and also where in the coding sequence the sgRNAs are targeting.
If we look at the Pax2/5/8.a CRISPR in juveniles, for instance, we see that on average (across 3 replicates), ~78% of juveniles in the negative control condition had visible CMN axons, representing the level of mosaicism we see at this stage. In the CRISPR, we see only an average of ~13% of juveniles had CMNs. This appears to indicate a ~83% reduction in CMN specification. The two

© 2024. Published by The Company of Biologists under the terms of the Creative Commons Attribution License (https://creativecommons.org/licenses/by/4.0/).

Pax2/5/8.a sgRNAs selected to be used in combination had overall efficacies estimated at 37% and 33%. If we simply assume an additive effect, we arrive at 70% combined efficacy (not counting on any synergistic effect). So it is not entirely inconsistent. Thus, although there are still many unknowns, the phenotypes we see are not inconsistent with the efficacies measured and also generally with past CRISPR experiments in Ciona.

26) I cannot find any reason, in the response letter, for saying “In animals overexpressing Pax2/5/8.a, we observed … no change in the presence or number of gill slit-innervating CMN axons”. To the contrary, the answer in the response letter indicates that the authors are not sure for this change. The authors need to revise the text.
We have corrected this. The uncertainty was implicitly but unclearly stated, but we have changed this to “we did not observe any obvious excess of gill slit-innervating Phox2+ axons”, conveying an absence of evidence, not evidence of absence. We have attempted to further clarify this in the text. The lack of clarity stems mostly from the fact that it is difficult to distinguish individual axons in the tracts that form exiting the CNS. However, we did not observe excess innervation of the gill slits. There are always at maximum two gill slits at this stage, so it is possible that the extent of axon targeting/innervation is limited by the target (i.e. gill slit epithelia).

30) It is a shame that no positive action was taken for this comment. The third reviewer also pointed out the same thing. There is no positive message that can be drawn from the scRNAseq data analysis.further
We have moved the scRNAseq analysis to the supplement. We still believe it offers 1) corroboration that key Pax2/5/8.a candidate target genes identified by bulk RNAseq are enriched in wild-type neck cells, and 2) evidence that there is compartmentalization within the neck between Phox2+ (or Phox2-high) and Phox2-negative (or –low) cells, and that other genes follow this pattern as well.

32) The revision is satisfactory. But gene expression patterns for FGF9, and EphrinA.b and Ephrin A.d may be reported previously in Imai et al., 2009.
They were reported and we have cited this paper as well, though our in situs show precise labeling of the motor ganglion cells (with the FGF8 reporter) that was lacking previously.

33) It is a shame that no positive action was taken for this comment, too. Without validation of action of the constructs or knockout, these data are not at all useful. Even if the authors have a policy to publish all data including negative ones, these data should not be included without validation experiments. From these data, we cannot know whether their experimental designs or materials did not work, or whether these signaling pathways were not involved.
We have included sgRNA validation data for Gli, while the ca/dn BMPRs were previously validated (Christiaen et al. 2010) However, we have further modified the text to be even more careful in our conclusions drawn from negative data. We are open to removing these data from the paper, if requested by the editor.

New Comment 1) Although most data are obtained by multiple rounds of experiments, some do not look so (these include data shown in Figure 4D, 7E, (6L?), S1F, S6F). I am not sure that all of these experiments are necessary for the conclusion of this paper. But if the authors think that these data should be included in their paper, showing reproducibility is the responsibility of the authors.
We have duplicated the experiment in Figure 6A-E, as the data are central to the conclusions in this manuscript.

2) The authors added a description about statistical tests in the Method section. They used Fisher's exact tests. I am not completely certain if Fisher's exact tests are appropriate. In most similar studies, individual embryos are not regarded as individual samples, but individual experiments are regarded as individual samples for statistical tests. For me, the latter looks more appropriate, but because I am not a statistician, I may be wrong.

We are aware of the lack of widely accepted standards for statistical analysis of scoring data in the field. We have tried to follow recent examples from respected Ciona labs, as in Vitrinel et al. 2023, which used Fisher’s exact tests. We have yet to receive clear guidance from statisticians or our peers on the most appropriate tests.

© 2024. Published by The Company of Biologists under the terms of the Creative Commons Attribution License (https://creativecommons.org/licenses/by/4.0/).

Resubmission

First decision letter

MS ID#: DEVELOP/2024/202719

MS TITLE: Specification and survival of post-metamorphic branchiomeric neurons in the hindbrain of a non-vertebrate chordate

AUTHORS: Eduardo Gigante, Katarzyna Piekarz, Alexandra Gurgis, Leslie Cohen, Florian Razy-Krajka, Sydney Popsuj, Christopher J Johnson, Hussan S Ali, Shruthi Mohana Sundaram, and Alberto Stolfi

My apologies for the long time it has taken us to obtain the report fromthe referees on your manuscript. I have now received the reports of two of the referees who reviewed the earlier version of your manuscript and I have reached a decision. The referees' comments are appended below, or you can access them online: please go to BenchPress and click on the 'Manuscripts with Decisions' queue in the Author Area.

As you will see, the reviewers are divided, with referee 1 still having significant concerns while referee 2 is much more positive and in favour of publication. After having looked again at the manuscript, I side with referee 2 and would like to publish a revised manuscript in Development, provided that you consider the suggestions and comments of both referees. Please revise your manuscript according to these recommendations when you think this is feasible and consider in particular transferring the scRNA-Seq reanalysis in the supplementary material as previously recommended by both referees. For the other comments, please respond to them in your point-by-point letter and if you do not agree with any of the criticisms or suggestions, explain clearly why this is so.

Reviewer 1

Advance summary and potential significance to field

The topic that this manuscript was addressing was very interesting, and I was looking forward to reading a revised version. But now I am very disappointed because the authors did not address my comments properly. The main conclusion or important message is drawn from the Figure 1 to Figure 3; the adult neurons start to be differentiating in larvae. However, in my opinion, this claim does not have solid evidence at all. I cannot agree with the publication of this manuscript in the present form. However, because what they want to prove from Figures 1 to 3 are important, I hope that another chance will be given to the authors, and that the authors will do more professional jobs for the next time. For revision, it might be a good idea to focus their work on the data from Figures 1 to 3 with more solid evidence, because the rest is not essential for the conclusion, and very preliminary.

Comments for the author

The following numbers correspond to the numbers in my previous review.
1) Title: Even if the neck region shares the evolutionary origin with the vertebrate hindbrain, the ascidian neck region cannot be called as the hindbrain. The current title may be very catchy but is misleading and incorrect. A scientifically correct title is more important than a sensational title, I believe.
3) Lineage: (A) To substantiate the authors’ claim that the CMNs are already differentiating in larvae, the authors need to show that these cells indeed have neurites. But I cannot see which cells extend the neurites even in the revised version. This point is central to this manuscript. In Figure

© 2024. Published by The Company of Biologists under the terms of the Creative Commons Attribution License (https://creativecommons.org/licenses/by/4.0/).

1H and I, it is not clear if the CMNs extend neurites. It is even possible that these come from NNs. This demonstration is the critical point of this manuscript but not sufficient. (B) Satisfactory. (C) Contrary to the response letter, no legend for Figure 1L was found. (D) I had misunderstood the data in my previous review. I am wondering if the other two reviewers may have misunderstood the data similarly. But the problem how the authors traced the CMN lineage stands anyway. GRIK>GFP supports the authors' idea, but it is not conclusive. As Phox>GFP starts to be expressed in CMNs only after metamorphosis, the same may occur for GRIK>GFP. The authors say "clear continuity between GRIK>GFP expression" in their answer to Reviewer1, but it is not clear from Figure 1. Stage 30 in Figure 1I corresponds to 25-27 hpf and Stage 36 corresponds to 45-60 hpf. Namely, there is a big gap between these two stages. This "lineage" problem is another critical point of this manuscript. This should be examined much more carefully. Dufor 2006 assumed that larval Phox+ cells gave rise to adult Phox+ cells. But this manuscript denies this. More concrete evidence is necessary. The way the third reviewer pointed out will be the best option. Live imaging or time-lapse imaging will also resolve this problem.
4) In Figure 6E, the authors claims that the anterior three cells are dpERK+ and the posterior one (fourth cell) is dpERK-. But the second cell looks to be dpERK- too. The second one may be in a different focal plane, but if so, show all focal planes necessary for substantiating the authors' claim.
5) line 292: "neck Neuron" should be "neck neuron".
9) If GFP signals are too weak and the authors cannot present evidence, the authors cannot say anything from this picture. Cells are not identifiable in Figure S3, either. Changing the sentence (line 109-110) is necessary. If the authors cite earlier studies and change wording, this part will be persuasive even without updating the data.
13) I was not able to find the "alternative interpretation", which is written in the response letter, in the revised version.
17) Now GRIK>GFP supports the idea that Phox2+ cells in adults are derived from A12.121, even though it is not conclusive. The explanation written in the response letter should be written in the main text of the manuscript.
23) I cannot figure out what percentages of genes in embryos were knocked out from Figure S7. Show them by numbers, but not by graphs only. Are these proportions consistent with proportions of embryos with phenotypes? These should be included in the manuscript.
26) I cannot find any reason, in the response letter, for saying "In animals overexpressing Pax2/5/8.a, we observed … no change in the presence or number of gill slit-innervating CMN axons". To the contrary, the answer in the response letter indicates that the authors are not sure for this change. The authors need to revise the text.
30) It is a shame that no positive action was taken for this comment. The third reviewer also pointed out the same thing. There is no positive message that can be drawn from the scRNAseq data analysis.
32) The revision is satisfactory. But gene expression patterns for FGF9, and EphrinA.b and Ephrin A.d may be reported previously in Imai et al., 2009.
33) It is a shame that no positive action was taken for this comment, too. Without validation of action of the constructs or knockout, these data are not at all useful. Even if the authors have a policy to publish all data including negative ones, these data should not be included without validation experiments. From these data, we cannot know whether their experimental designs or materials did not work, or whether these signaling pathways were not involved.

New Comment 1) Although most data are obtained by multiple rounds of experiments, some do not look so (these include data shown in Figure 4D, 7E, (6L?), S1F, S6F). I am not sure that all of these experiments are necessary for the conclusion of this paper. But if the authors think that these data should be included in their paper, showing reproducibility is the responsibility of the authors.
2) The authors added a description about statistical tests in the Method section. They used Fisher's exact tests. I am not completely certain if Fisher's exact tests are appropriate. In most similar studies, individual embryos are not regarded as individual samples, but individual experiments are regarded as individual samples for statistical tests. For me, the latter looks more appropriate, but because I am not a statistician, I may be wrong.

Reviewer 2

Advance summary and potential significance to field

© 2024. Published by The Company of Biologists under the terms of the Creative Commons Attribution License (https://creativecommons.org/licenses/by/4.0/).

N/A

Comments for the author

I am happy with the revisions and support publication of this study in Development.

First revision

Author response to reviewers' comments

We thank the reviewers and editor for their comments and the opportunity to revise the manuscript. See our replies to each comment below:

Reviewer 1 Comments for the author
The following numbers correspond to the numbers in my previous review.
1) Title: Even if the neck region shares the evolutionary origin with the vertebrate hindbrain, the ascidian neck region cannot be called as the hindbrain. The current title may be very catchy but is misleading and incorrect. A scientifically correct title is more important than a sensational title, I believe.
Following the reviewer's suggestion we have changed our title to "Specification and survival of post-metamorphic branchiomeric neurons in a non-vertebrate chordate".

3) Lineage: (A) To substantiate the authors' claim that the CMNs are already differentiating in larvae, the authors need to show that these cells indeed have neurites. But I cannot see which cells extend the neurites even in the revised version. This point is central to this manuscript. In Figure 1H and I, it is not clear if the CMNs extend neurites. It is even possible that these come from NNs. This demonstration is the critical point of this manuscript but not sufficient.
Based on our experience imaging neurons in the Ciona larva using fluorescent reporters, we concluded that the axon projecting posteriorly towards the tail is the only Neck Neuron-derived neurite, while the others are coming from the more anterior delaminated cells, the putative CMNs. We have tried to present additional images that make this clearer, from a wider range of examples of imaged larvae using the contrast between nuclear H2B::mCherry and cytoplasmic Unc-76::GFP to better delineate cells and identify which neurites might be coming from which cells. These data have been included as Supplemental Figure 2.
(C) Contrary to the response letter, no legend for Figure 1L was found.
We apologize for the confusion; the relevant legend (2L) was present in the figure legend of our original submission but was lost in the final revised file that was re-submitted. It has been correctly replaced in the latest resubmission
(D) I had misunderstood the data in my previous review. I am wondering if the other two reviewers may have misunderstood the data similarly. But the problem how the authors traced the CMN lineage stands anyway. GRIK>GFP supports the authors' idea, but it is not conclusive. As Phox>GFP starts to be expressed in CMNs only after metamorphosis, the same may occur for GRIK>GFP. The authors say "clear continuity between GRIK>GFP expression" in their answer to Reviewer1, but it is not clear from Figure 1. Stage 30 in Figure 1I corresponds to 25-27 hpf and Stage 36 corresponds to 45-60 hpf. Namely, there is a big gap between these two stages. This "lineage" problem is another critical point of this manuscript. This should be examined much more carefully. Dufor 2006 assumed that larval Phox+ cells gave rise to adult Phox+ cells. But this manuscript denies this. More concrete evidence is necessary. The way the third reviewer pointed out will be the best option. Live imaging or time-lapse imaging will also resolve this problem.
To address this question, we first attempted to perform live imaging time-lapse experiments in metamorphosing individuals expressing the GRIK>GFP reporter between 22 and 44hpf. Due to the fact that these individuals were not fixed/cleared and thus were more optically opaque, and due to the complex movements of the animal, tissues, and cells during body rotation, we were unable to resolve and track the thin neurites through metamorphosis.
However, we were able to successfully carry out tracking via photoconversion of Kaede, as now shown in the new Figure 3. We photoconverted Kaede driven by the GRIK promoter, which is only

© 2024. Published by The Company of Biologists under the terms of the Creative Commons Attribution License (https://creativecommons.org/licenses/by/4.0/).

active in the delaminated, Phox2-negative cells in the Neck during larval stage. We let these individuals undergo metamorphosis, in parallel with individuals electroporated with the same GRIK>Kaede reporter but NOT subjected to photoconversion. In the non-photoconverted juveniles, we see green Kaede signal but no red signal, indicating the successful lack of photoconversion in the control. In contrast, we see red Kaede signal in the juvenile CNS of individuals who were subjected to photoconversion. These results are consistent with the survival of GRIK+ larval cells through metamorphosis. Unfortunately, we were not able to make yet a functional Unc-76-tagged Kaede that can fill the axons effectively. Kaede forms tetramers, which limits its use in protein fusions. Future work will need to address the problem of tracking the neurites/axons through metamorphosis.
We have now updated the text to incorporate our Kaede photoconversion results, and discuss its implications. We suggest that it is possible that there may be some variability in which larval cells (Phox2+ or GRIK+) give rise to which CMNs. As the CMNs will undoubtedly multiply as the individual grows and additional gill slits, or stigmata, appear, there seems to be the need for a pool of undifferentiated CMN progenitors through later stages of post-metamorphic growth, which could be fulfilled by the Phox2+ cells. It may be that GRIK+ cells seen in the larva are indeed the earliest differentiating CMNs, but that their survival and further differentiation might be dictated by the timing and speed of metamorphosis. It is possible that in some cases in which settlement or feeding might be delayed (like during overwintering), early differentiating CMNs could plausibly regress/senesce, to be replaced later by later-differentiating cells. Eventually, all CMNs appear to express both Phox2 and GRIK reporters, making this technically quite challenging to study given our currently available approaches.

4) In Figure 6E, the authors claims that the anterior three cells are dpERK+ and the posterior one (fourth cell) is dpERK-. But the second cell looks to be dpERK- too. The second one may be in a different focal plane, but if so, show all focal planes necessary for substantiating the authors' claim.
We have now provided more quantitative analysis of the dpERK staining across the neck in both wild-type and dnFGFR conditions, and we see some differences in the 2nd cell relative to the 1st and 3rd cell (hinting at possible mechanisms of further heterogeneity seen in the anterior neck), but staining is still stronger than in the 4th cell. And of course all staining is uniformly negative in the dnFGFR condition. These data have been included as Supplemental Figure 7.
Minor Comments
5) line 292: “neck Neuron” should be “neck neuron”.
All instances have been corrected.

9) If GFP signals are too weak and the authors cannot present evidence, the authors cannot say anything from this picture. Cells are not identifiable in Figure S3, either. Changing the sentence (line 109-110) is necessary. If the authors cite earlier studies and change wording, this part will be persuasive even without updating the data.
We have provided an updated figure with clearer GFP expression. We repeated the experiment using a mouse anti-GFP antibody to detect transient, low-level GFP expression that might be too low or early to detect via direct fluorescence (given the maturation time of GFP). With GFP antibody staining, we observed all four Pax2/5/8+ cells were GFP+ during the mid-tailbud stage. We note that several hours later during the larval phase, Phox2>GFP is no longer discernible to in the four posterior, delaminated Pax2/5/8+ cells, in contrast to the bright expression in the anterior cells. We conclude that the early expression of Phox2 at the mid-tailbud stage is likely transient, low-level expression, which has been observed for other genes and/or reporters in Ciona (e.g. MRF in the B7.5 lineage as described in Tolkin and Christiaen 2016). We conclude that after the mid-tailbud stage, Phox2 is downregulated in the posterior, delaminated cells and GFP expressed by the Phox2 promoter in our reporter plasmid early is not high enough or does not persist long enough for those posterior cells to inherit GFP at easily detected levels. This suggests that the high-level expression of Phox2>GFP in the ependymal neck cells of the larva is due to sustained/upregulated expression. Together, we hypothesize that Phox2 expression in the Neck throughout embryogenesis and metamorphosis is not constant and is highly dynamic. Further studies on the temporal dynamics of Phox2 regulation and function are ongoing our lab. We have updated the figure and text to accommodate these new data.

13) I was not able to find the “alternative interpretation”, which is written in the response letter, in the revised version.

© 2024. Published by The Company of Biologists under the terms of the Creative Commons Attribution License (https://creativecommons.org/licenses/by/4.0/).

We apologize for not making these alternative interpretations more explicit, we have now clearly stated our alternative interpretations in the manuscript (highlighted).

17) Now GRIK>GFP supports the idea that Phox2+ cells in adults are derived from A12.121, even though it is not conclusive. The explanation written in the response letter should be written in the main text of the manuscript.
Actually, in adults, GRIK and Phox2 reporters are co-expressed, suggesting that even though in the larva the expression domains of GRIK and Phox2 are mutually exclusive, they are merge at some point. This might be tied to possible variability in CMN specification. We have expanded on this point in the discussion.

23) I cannot figure out what percentages of genes in embryos were knocked out from Figure S7. Show them by numbers, but not by graphs only. Are these proportions consistent with proportions of embryos with phenotypes? These should be included in the manuscript.
We have updated the figure (we assume the old Figure S8, now Figure S10) with the numerical efficacy values given by the sequencing service company (Azenta). However, for Phox2 this number is a slight overestimation due to the low frequency (~2.5%) of a small naturally occurring indel in the analyzed region.
The overall efficacies of the sgRNAs are validated by using a ubiquitously-expressing promoter (Eef1a) to drive expression of Cas9. The actual efficacy in the specific lineage analyzed (e.g. the neck) will vary from the overall efficacy based on the strength/timing of the lineage-specific driver used to drive Cas9 for phenotyping experiments, the number of sgRNAs used, the relative amounts of plasmid electroporated. The actual relationship with phenotype will further vary according to haploinsufficiency, the likelihood that a frameshift will occur upon indel repair, and also where in the coding sequence the sgRNAs are targeting.
If we look at the Pax2/5/8.a CRISPR in juveniles, for instance, we see that on average (across 3 replicates), ~78% of juveniles in the negative control condition had visible CMN axons, representing the level of mosaicism we see at this stage. In the CRISPR, we see only an average of ~13% of juveniles had CMNs. This appears to indicate a ~83% reduction in CMN specification. The two Pax2/5/8.a sgRNAs selected to be used in combination had overall efficacies estimated at 37% and 33%. If we simply assume an additive effect, we arrive at 70% combined efficacy (not counting on any synergistic effect). So it is not entirely inconsistent. Thus, although there are still many unknowns, the phenotypes we see are not inconsistent with the efficacies measured and also generally with past CRISPR experiments in Ciona.

26) I cannot find any reason, in the response letter, for saying “In animals overexpressing Pax2/5/8.a, we observed … no change in the presence or number of gill slit-innervating CMN axons”. To the contrary, the answer in the response letter indicates that the authors are not sure for this change. The authors need to revise the text.
We have corrected this. The uncertainty was implicitly but unclearly stated, but we have changed this to “we did not observe any obvious excess of gill slit-innervating Phox2+ axons”, conveying an absence of evidence, not evidence of absence. We have attempted to further clarify this in the text. The lack of clarity stems mostly from the fact that it is difficult to distinguish individual axons in the tracts that form exiting the CNS. However, we did not observe excess innervation of the gill slits. There are always at maximum two gill slits at this stage, so it is possible that the extent of axon targeting/innervation is limited by the target (i.e. gill slit epithelia).

30) It is a shame that no positive action was taken for this comment. The third reviewer also pointed out the same thing. There is no positive message that can be drawn from the scRNAseq data analysis.further
We have moved the scRNAseq analysis to the supplement. We still believe it offers 1) corroboration that key Pax2/5/8.a candidate target genes identified by bulk RNAseq are enriched in wild-type neck cells, and 2) evidence that there is compartmentalization within the neck between Phox2+ (or Phox2-high) and Phox2-negative (or –low) cells, and that other genes follow this pattern as well.

32) The revision is satisfactory. But gene expression patterns for FGF9, and EphrinA.b and Ephrin A.d may be reported previously in Imai et al., 2009.
They were reported and we have cited this paper as well, though our in situs show precise labeling of the motor ganglion cells (with the FGF8 reporter) that was lacking previously.

© 2024. Published by The Company of Biologists under the terms of the Creative Commons Attribution License (https://creativecommons.org/licenses/by/4.0/).

33) It is a shame that no positive action was taken for this comment, too. Without validation of action of the constructs or knockout, these data are not at all useful. Even if the authors have a policy to publish all data including negative ones, these data should not be included without validation experiments. From these data, we cannot know whether their experimental designs or materials did not work, or whether these signaling pathways were not involved.
We have included sgRNA validation data for Gli, while the ca/dn BMPRs were previously validated (Christiaen et al. 2010) However, we have further modified the text to be even more careful in our conclusions drawn from negative data. We are open to removing these data from the paper, if requested by the editor.

New Comment 1) Although most data are obtained by multiple rounds of experiments, some do not look so (these include data shown in Figure 4D, 7E, (6L?), S1F, S6F). I am not sure that all of these experiments are necessary for the conclusion of this paper. But if the authors think that these data should be included in their paper, showing reproducibility is the responsibility of the authors.
We have duplicated the experiment in Figure 6A-E, as the data are central to the conclusions in this manuscript.

2) The authors added a description about statistical tests in the Method section. They used Fisher's exact tests. I am not completely certain if Fisher's exact tests are appropriate. In most similar studies, individual embryos are not regarded as individual samples, but individual experiments are regarded as individual samples for statistical tests. For me, the latter looks more appropriate, but because I am not a statistician, I may be wrong.

We are aware of the lack of widely accepted standards for statistical analysis of scoring data in the field. We have tried to follow recent examples from respected Ciona labs, as in Vitrinel et al. 2023, which used Fisher's exact tests. We have yet to receive clear guidance from statisticians or our peers on the most appropriate tests.

Second decision letter

MS ID#: DEVELOP/2024/202719

MS TITLE: Specification and survival of post-metamorphic branchiomeric neurons in a non-vertebrate chordate

AUTHORS: Eduardo Gigante, Katarzyna Piekarz, Alexandra Gurgis, Leslie Cohen, Florian Razy-Krajka, Sydney Popsuj, Christopher J Johnson, Hussan S Ali, Shruthi Mohana Sundaram, and Alberto Stolfi

I am delighted to tell you that your manuscript has been accepted for publication in Development, pending our standard ethics checks.

© 2024. Published by The Company of Biologists under the terms of the Creative Commons Attribution License (https://creativecommons.org/licenses/by/4.0/).
